# A systematic mixed studies review on Organizational Participatory Research: towards operational guidance

**DOI:** 10.1186/s12913-018-3775-5

**Published:** 2018-12-22

**Authors:** Paula Louise Bush, Pierre Pluye, Christine Loignon, Vera Granikov, Michael T. Wright, Carol Repchinsky, Jeannie Haggerty, Gillian Bartlett, Sharon Parry, Jean-François Pelletier, Ann C. Macaulay

**Affiliations:** 1 Department of Family Medicine, McGill University, 5858 Côte-des-Neiges, Suite 300, Montréal, Quebec, H3S 1Z1 Canada; 2 Department of Family Medicine, Sherbrooke University, 150 Place Charles Lemoyne suite 200, Longueuil, Quebec, J4K 0A8 Canada; 3 Institute for Social Health, Catholic University of Applied Sciences Berlin, Köpenicker Allee 39-57, 10318 Berlin, Germany; 4 Special projects, Canadian Pharmacists Association, 1785 Alta Vista Drive, Ottawa, ON K1G 3Y6 Canada; 5 West Island YMCA, 230 Brunswick Blvd, Pointe-Claire, Quebec, H9R 5N5 Canada; 6 Mental health research institute, University of Montreal, Montreal, Canada; 7 CIET/Participatory Research at McGill (PRAM), 5858 Cote de Neiges, 3rd floor, Montreal, Montreal, QC H3S 1Z1 Canada

**Keywords:** Participatory research, Organizational participatory research, Organizational change, Practice change, Qualitative synthesis, Mixed studies review, Healthcare organization

## Abstract

**Background:**

Organizational Participatory Research (OPR) seeks organizational learning and/or practice improvement. Previous systematic literature reviews described some OPR processes and outcomes, but the link between these processes and outcomes is unknown. We sought to identify and sequence the key processes of OPR taking place with and within healthcare organizations and the main outcomes to which they contribute, and to define ideal-types of OPR.

**Methods:**

This article reports a participatory systematic mixed studies review with qualitative synthesis A specialized health librarian searched MEDLINE, CINAHL, Embase Classic + Embase, PsycINFO, the Cochrane Library, Social Work Abstracts and Business Source Complete, together with grey literature data bases were searched from inception to November 29, 2012. This search was updated using forward citation tracking up to June 2014. Reporting quality was appraised and unclear articles were excluded. Included studies clearly reported OPR where the main research related decisions were co-constructed among the academic and healthcare organization partners. Included studies were distilled into summaries of their OPR processes and outcomes, which were subsequently analysed using deductive and inductive thematic analysis. All summaries were analysed; that is, data analysis continued beyond saturation.

**Results:**

Eighty-three studies were included from the 8873 records retrieved. Eight key OPR processes were identified. Four follow the phases of research: 1) form a work group and hold meetings, 2) collectively determine research objectives, 3) collectively analyse data, and 4) collectively interpret results and decide how to use them. Four are present throughout OPR: 1) communication, 2) relationships; 3) commitment; 4) collective reflection. These processes contribute to extra benefits at the individual and organizational levels. Four ideal-types of OPR were defined. Basic OPR consists of OPR processes leading to achieving the study objectives. This ideal-type and may be combined with any of the following three ideal-types: OPR resulting in random additional benefits for the individuals or organization involved, OPR spreading to other sectors of the organization and beyond, or OPR leading to subsequent initiatives. These results are illustrated with a novel conceptual model.

**Conclusion:**

The model provides operational guidance to help OPR stakeholders collaboratively address organizational issues and achieve desired outcomes and more.

**Review registration:**

As per PROSPERO inclusion criteria, this review is not registered.

**Electronic supplementary material:**

The online version of this article (10.1186/s12913-018-3775-5) contains supplementary material, which is available to authorized users.

## Background

Collaborative approaches to research involve academic researchers partnering with those who have a stake in the research, but do not necessarily have any formal research training. Stakeholders may include patients, policy makers, communities, organizations or any other individual or group who may benefit from or use the results [1–3]. The premise is that the non-academic stakeholders have insight into problems and their potential solutions [2]. The non-academic stakeholders may participate in identifying the problem and formulating the research questions, selecting the research methods, collecting the data, analysing the data, interpreting the results, and applying and disseminating results. Throughout these stages of the research process, non-academic stakeholders may participate to varying degrees. Typologies of this participation have been put forth illustrating continua from the non-academics participating passively (e.g., providing input, but not actively engaging in decisions) to taking full control of the study [3–6]. Further, participatory research can be distinguished by the three main drivers of the various research approaches, namely knowledge translation, social and environmental justice, and self-determination [1]. Herein, we focus on organizational participatory research (OPR) which is most closely aligned with the knowledge translation driver.

An organization is a “context of action in which relationships of cooperation, exchange, and conflict between actors with divergent interests are being established and managed” [7] and which fluctuates in response to changes in the environment. OPR takes place with and within organizations—in this article, organizations offering healthcare-related services—for the purpose of organizational learning and/or practice improvement [8–11]. This research approach is rooted in the works of Kurt Lewin on action research [12] and of Chris Argyris and Donald Schön on action science [8]. It is a capacity building endeavour where capacity building is a process or an approach used to develop, enhance, or leverage a collection of characteristics (e.g., organization stakeholders’ insider knowledge, academic stakeholders’ research methods expertise) for a purpose (e.g., effecting sustainable organizational change) that is context dependent [13].

Extant reviews of participatory forms of research taking place within healthcare organizations and with their stakeholders (practitioners, staff, management, or service users) report positive outcomes, such as stakeholders gaining confidence, skills, and knowledge; addressing challenges and implementing innovations; improved job and patient satisfaction [3, 14–16]. However, attributing the outcomes to the participatory processes has been difficult. A minimum level of non-academic partner participation needed to guarantee success has not been determined, either [3]. Furthermore, these reviews examined studies that were heterogeneous regarding the type and timing of organization stakeholders’ participation. Finally, some reported difficulty applying detailed frameworks of participation as the reviewed studies were often lacking detail [3, 15]. Therefore, to gain clarity regarding how participatory processes contribute to outcomes, we used a framework of two distinct forms of organization stakeholder participation, at opposite ends of the continuum: (1) providing input when consulted by academics (no research co-governance) for at least three research decisions: (a) identification of the research question(s); (b) determination of the methodology, and/or data collection and/or analysis, and/or interpretation of results; (c) implementation or dissemination of results; or (2) co-constructing at least these three research decisions with academic partners (research co-governance) [9]. Regarding OPR processes, we focussed on any academic-organization collaborative activity throughout all phases of a study. We conceived of an OPR outcome as any consequence of an OPR process.

We used a sequential mixed methods review design where the quantitative synthesis of phase-1 informed the qualitative synthesis of phase-2 [17–20]. Phase-1, reported elsewhere [9], measured the likelihood of an OPR study yielding ‘extra benefits’. We define ‘extra benefits’ as positive outcomesof an OPR study that clearly do not meet the specific participatory research project objective(s) for change (for a complete definition, see PL Bush, P Pluye, C Loignon, V Granikov, MT Wright, J-F Pelletier, G Bartlett-Esquilant, AC Macaulay, J Haggerty, S Parry, et al. [9]). We compared OPR studies that clearly represented either Co-construction OPR (*n* = 83) or consultation OPR (*n* = 24). We found no significant association between the form of participation and the likelihood of at least one extra benefit of OPR. Yet, when the OPR was initiated by the organization (as opposed to the academic stakeholders or the organization and academic stakeholders together), the likelihood of the study resulting in at least one extra benefit was quadrupled (OR = 4.11, CI = 1.21–14.01) [9].

This paper reports phase-2 of the review with the following three objectives: 1) to identify OPR processes and the outcomes to which they contribute (descriptive objective); 2) to understand the sequence of OPR processes and outcomes, in particular extra benefits (analytical objective); 3) to identify ideal-types of OPR process-outcome sequences (explanatory objective). OPR is an inherently chaotic or ‘messy’ [21] endeavour. Identifying characteristics of OPR in the empirical literature and assembling them into ideal types [22] may help to provide some order to our understanding of this messy research approach. The ultimate goal of this mixed studies review is to provide recommendations for OPR practice to help academic and organization research teams achieve their agreed upon objectives and experience extra benefits; thereby, helping to ensure the added value of the participatory approach to their research.

## Methods

A participatory research approach was used to conduct this systematic review which followed a process as outlined in QN Hong, P Pluye, M Bujold and M Wassef [19]. Specifically, academic researchers and health organization stakeholders on our team co-constructed the research questions, the interpretation of results and the resulting publications. Moreover, some academic and organization stakeholders contributed to various other stages of the review including developing the bibliographic data base search strategy, examining raw data, guiding the analysis, and discussing preliminary results. In this second phase of the review, the subset of studies incorporating a co-construction form of health organization stakeholder participation from Phase-1 was included. The rationale for this purposeful sample was that these studies provide a richer description of participatory process and their consequences. As per PROSPERO inclusion criteria, this review is not registered. The Enhancing Transparency in Reporting the Synthesis of Qualitative Research (ENTREQ) framework guided the reporting of the synthesis [23].

### Search methods

Peer reviewed and grey literature databases were searched from inception through 2012 using MEDLINE, CINAHL, Embase Classic + Embase, PsycINFO, the Cochrane Library, Social Work Abstracts, Business Source Complete, ProQuest Dissertations & Theses database, the New York Academy of Medicine – Grey Literature Report, OpenGrey, and Google. The search was updated in 2014 using forward citation tracking. See Additional file 1 for search strategies. Full details regarding search methods are reported in PL Bush, P Pluye, C Loignon, V Granikov, MT Wright, J-F Pelletier, G Bartlett-Esquilant, AC Macaulay, J Haggerty, S Parry, et al. [9].

### Search outcome

The database search identified 13,837 records which were exported to EndNote where duplicates were removed. Forward citation tracking led to an additional 150 records for a total of 8873 unique records. As described elsewhere [9], nine inclusion criteria were iteratively developed with all research team members. Two independent reviewers screened the records for relevance and selected 992 full texts for further assessment. In all, 140 studies met the inclusion criteria. (See PL Bush, P Pluye, C Loignon, V Granikov, MT Wright, J-F Pelletier, G Bartlett-Esquilant, AC Macaulay, J Haggerty, S Parry, et al. [9] for full details and flow chart.)

### Quality appraisal

The quality appraisal focused on identifying studies reporting an explicit link between OPR processes and outcomes; 33 studies were excluded because there was no such statement. Among the 107 studies included in phase-1, 83 represent the co-construction type of participation and were thus selected for phase-2.

### Data extraction

For each included study, the lead author and two research assistants extracted descriptive data, study objectives, and text passages describing OPR processes, OPR outcomes, and OPR processes explicitly linked with OPR outcomes (Additional file 2). For the purposes of this review, OPR processes that are explicitly linked to outcomes are ‘key processes’ of OPR. Moreover, OPR outcomes are not the study results, but rather anything that occurs following an OPR process. For instance, an OPR outcome that is explicitly linked to an OPR process could be a statement such as: “working together on the OPR project helped to develop the synergy of the partnership”. In this example, the OPR process is ‘working together’ and the partnership synergy is the OPR outcome. Thus, we reviewed the authors’ descriptions of the OPR processes they used and the outcomes they said occurred as a result (in the above example, working together *helped to develop* the synergy of the partnership). Consequently, many data were extracted from the methods and discussion sections. For studies described across multiple publications, all papers were used, and data were extracted in order, beginning with the earliest publication. Data were entered into one Excel workbook per study with the descriptive and process-outcome data on separate pages. The process-outcome page had one column for processes and one for outcomes. OPR process and outcome passages were pasted in order, one per row. Text passages that made an explicit link between an OPR process and outcome were pasted on the same row, and the process and outcome cells were merged. The top row was reserved for the study objective. The resulting process-outcome pages of each Excel workbook presented a clear story of the study objective, the OPR processes used, and the OPR outcomes observed. The lead author reviewed all Excel workbooks for accuracy of extracted data and of the categorisation of text excerpts as OPR process and/or outcome.

### Synthesis

The co-authors of this study are academic and organization stakeholders with experience in OPR. As per a participatory research approach, co-authors participated in decisions throughout this review. Regarding this qualitative synthesis, co-authors commented on the Excel documents prepared in the preceding phase. Then, the lead author copied the process-outcome pages of each Excel workbook into MS Word, removed redundancies, and re-arranged the excerpts into OPR summaries with: (1) objective(s), people and place; (2) OPR processes; (3) linked OPR processes and outcomes. This reflexive step was necessary because much of the data were not the product of empirical investigation, but rather the authors’ reflections on their practical experience with OPR. Yet, all included studies were peer reviewed and most were co-authored by a team of investigators. For the most part, the OPR summaries (raw data) are the study authors’ words; although, some excerpts were rewritten to ensure proper grammar and syntax or paraphrased for the sake of parsimony.

To identify processes linked with outcomes and to understand their sequences throughout an OPR (objectives 1 and 2), the lead author used hybrid (inductive/deductive) thematic analysis [24]. She coded all of the OPR summaries (assigned extracts of text to themes) according to OPR process, OPR outcome, and linked OPR process-outcome using qualitative data analysis software (NVivo 10). Coding the data pertaining only to an OPR process or only to an OPR outcome was important to gain an understanding of the data corpus. However, given the specific objectives of this review, the final synthesis is based solely on the linked process-outcome codes. This coding was guided by partnership synergy theory [25, 26] capacity building [27, 28] and benefits of participatory research [29] frameworks (deductive coding). Themes and codes outside of these frameworks were also used where the data suggested them (inductive coding).

In line with traditional guidance for consistency and rigour in qualitative thematic data analysis [30] and its application as ‘qualitative thematic synthesis’ in literature reviews [31], our synthesis is based on an interpretative method and research meetings, where coding processes were shared and discussed. Through iterations of analysis and discussion of preliminary results with team members, the analysis became increasingly inductive. All 83 summaries were coded, rather than stopping the analysis once saturation had been reached. All results reported herein were found in multiple summaries. The lead author aggregated the codes into OPR process-outcome themes and wrote an overall OPR process-outcome story depicting themes that occur at the beginning of an OPR and others that occur during and toward the end of an OPR. Co-authors revised this story commenting, in particular, on its credibility. The lead author delved back into the data and refined the analysis to address co-authors’ comments. The continued interaction among the university and organization co-authors was critical to recognize, discuss and deal with our biases. Additionally, preliminary findings were presented at academic conferences over the course of the analysis process, and discussions with academic researcher and health practitioner delegates provided further perspectives that were taken into account.

To identify ideal types of OPR (objective 3), the lead author examined the process-outcome sequences that occur toward the end of an OPR and proposed types based on ultimate OPR outcomes. These OPR ideal types were verified and refined during a grouping exercise [32]. Two research assistants with expertise in OPR (RS & JH) and two co-authors (PLB & PP) met to read and discuss the 83 summaries in relation to the proposed types. They grouped the summaries according to the dominant OPR type and defined ideal-types [33] of OPR.

## Results

### Characteristics of included studies

In total, 83 studies described across 145 publications, met our inclusion criteria. The complete results of our search and selection processes are published elsewhere [9]. The 83 studies were each reduced to an OPR summary of 450 words, on average (Additional file 3). The coding trees are in Additional file 4. To respond to our first two objectives, below we detail the eight key OPR processes that were identified (objective 1). Four of these occur during subsequent research phases, and four are present throughout an OPR. We then describe and exemplify the extra benefits for the organization stakeholders and the organization as a whole that the OPR processes contribute to (objective 1). These OPR processes and OPR outcomes are presented in our results following the sequence in which they occur in OPR (objective 2). Finally, we present four ideal-types [33] of OPR (objective 3). Each is illustrated with an OPR summary. To lend credibility to our findings and to help readers relate to them and determine their applicability to their contexts, excerpts from the OPR summaries (raw data) are included throughout the results section. When the excerpts are direct quotations from the original publications, they are indicated in italic text.

### Eight key OPR processes

#### Four phases of OPR

We begin by illustrating the four key processes of OPR that describe the sequence of activities that should be carried out to help achieve extra benefits from the OPR approach.

##### Form a working group (WG) and hold meetings

Typically, a WG of university and organization stakeholders is formed to carry out the OPR. Organization stakeholders vary depending on the needs of the project, but a multidisciplinary WG is common (e.g., nurse, physician, manager, social worker, etc.). Many conclude that diverse membership is crucial and can help ensure the relevance and uptake of the OPR. For instance, some assert *“the diverse blend of experts from the [organization] and the academic investigators has created projects and programs that are better suited to and accepted by the community and with greater chance for sustainability than could have been produced by either group independently*” [34].

Regarding administration’s and management’s participation in OPR, L Hamelin Brabant, M Lavoie-Tremblay, C Viens and L Lefrancois [35] write that administrators’ involvement helped to “*instil the motivation needed to implement new practices*.” Others suggest roles management can assume, such as supporting the OPR by reducing red tape, acknowledging progress reports by letter, implementing some recommendations, and encouraging staff to become actively involved in the process.

The reviewed studies suggest that WGs meet regularly, though the frequency of meetings varies (once per week to once every month or two, or less often). Meetings must be facilitated effectively and often an academic partner assumes this role. In one study, WG members “believed the project was well-managed and that this was a factor in its success” [36, 37]. This may suggest the importance of WG meetings being structured and focussed on specific issues or tasks, as described by some author teams.

WG meetings provide opportunities for the interaction, discussion, debate, reflection, and consensus required to enhance the OPR and achieve more nuanced results and products. During meetings, members complete OPR tasks, make decisions, learn from the experience of others, become more familiar with one another, and understand each other more in their various professional contexts, roles, and contributions--ultimately changing and improving practice. Furthermore, the OPR processes contribute to improved professional relationships and collaborations and increased job satisfaction. S Andrews, E Lea, T Haines, J Nitz, B Haralambous, K Moore, K Hill and A Robinson [38] explain that WG members’ “collaborative working challenged the traditional hierarchical relationships between staff and opened up an opportunity to work in partnership, thus building capacity and empowering staff.”

WG members typically value and benefit from the meetings. In one study, WG members “*felt that the [OPR] process was worthwhile. All staff agreed that ‘the meetings were valuable because we could discuss different ideas’. Generally, staff welcomed the opportunity of raising issues and discussing ways that these could be dealt with”* [39].

##### Collectively determine research objectives

The WG begins by determining objectives that all members consider valuable. This process helps members to develop relationships and contributes to the success of the OPR by, for example, helping WG members to “feel great enthusiasm about the project” [40]. Some suggest that establishing project goals quickly *“helps promote unity between the participants working towards this chosen end”* [41]. M Wallis and S Tyson [42] discuss how they determined their OPR objective:

The OPR process began with a number of meetings between members of the team, in which the nature and scope of the problems with practice were explored and critically examined. A multidisciplinary team decided to develop a chemotherapy protocol manual (based on analysis of the data related to timing of the administration protocols and reflection on these results) that would explain all the requirements of each protocol and provide more accurate estimations of the time required to administer the protocol and care for the patient.

While it can be challenging to reconcile differing perspectives and priorities of WG members, addressing organisation stakeholders’ needs can lead to more relevant and longer lasting changes, as underscored by H Waterman, R Harker, H MacDonald, R McLaughlan and C Waterman [43]: “*We would argue that, once the project had reconfigured its interests to those of patients, more detailed and lasting suggestions were implemented.*” Furthermore, R Khresheh and L Barclay [40] write: “s*hared goals guided the researcher and practitioners in their joint work and created commitment for the considerable effort needed for the research to succeed.”*

##### Collectively analyse data

Collective, iterative data analysis provides opportunities for WG members to exchange thoughts, understand each other’s perspectives, and reflect as a group. This helps to further develop relationships. For instance, EM Eisenberg, J Baglia and JE Pynes [44] note that “*working through the narratives with the ER staff gave them a role as research partners and enabled a dialogue that encouraged the ER staff to acknowledge and understand each other’s ways of viewing the world.”* Two studies describe what collective data analysis may entail. B Taylor [45] writes: “*Descriptions of participant observation were analysed individually using a reflective analysis method and collectively by group discussion.”* For their part, A Gregorowski, E Brennan, S Chapman, F Gibson, K Khair, L May and A Lindsay-Waters [46] indicate:

*The nurse consultants met with the research fellow on a regular basis to analyse the data together. In this way, a number of themes were collaboratively identified and divided into subthemes through paired and group work. Once themes were identified, the nurse consultants agreed to work in pairs on the individual themes. Each took a theme to work on as the primary researcher and another to work on as the secondary researcher. The group then came together to organise the themes and subthemes into a framework.*

##### Collectively interpret results and decide how to use them

Discussing and interpreting results during WG meetings can be validating for organization stakeholders when the results confirm their perceptions*.* Additionally, WG members can learn how to use study results to make evidence informed decisions about practice, and their motivation to effect change may increase given the awareness they gain regarding the issues under study. One author team writes: *“by creating a structured and supportive environment for data interpretation, the study reduced their fear. As they became more familiar with the charts and graphs, they began to look at data as a basis for decision making”* [47]. KT Ngwerume and M Themessl-Huber [48] provide another example: “*Discussions about challenges in utilizing this knowledge also made them aware of the difficulties in applying this new knowledge in practice and at the same time provided them with options about how to implement changes*.”

#### Four continuous OPR processes

Our analyses suggest that an additional four key processes are present throughout a successful OPR: communication, relationships development, commitment, and collective reflection. First, effective communication is open, two-way, transparent (e.g., making findings accessible), ongoing, and requires language all OPR team members understand. To exemplify, L Olsen and L Wagner [49] write: “*Using broadly defined terms (e.g., for ‘discharge’ and ‘prevention’) helped participants to find some common ground, despite their different backgrounds and mandates.”*

The quality of the communication within the WG can enhance stakeholder involvement and influence the OPR, help pursue issues to stakeholders’ satisfaction, improve team work and team spirit, and help to bring about change, as described by J Bothe and J Donoghue [50]:

*As a result of the opportunity to communicate openly with others, in addition to the team’s ability to think and discuss their work critically, their practice became more effective, safer for patients and patient centred. These changes were apparent to others, and provided a model of enablement that is now used elsewhere in the organization.*

The meeting facilitator plays an important role by fostering an environment of trust and respect to enhance the sharing of diverse viewpoints. K Galvin, C Andrewes, D Jackson, S Cheesman, T Fudge, R Ferris and I Graham [51] note that sharing views during meetings “*facilitated collaborative working and provided an opportunity to clarify any confusion, air any tensions, and to agree a way forward*.*”* Producing and circulating meeting notes or other documents is one communication method some have used. This can help *“to reflect on discussions and ignite new thoughts and deliberations*” [52], and “*correct any misunderstandings”* [53]. In one study, a public website was used to post information about the OPR:

*Interestingly, even when a member could not attend a session, reading the archive allowed them to feel that they had participated and resume without disruption. There was no evidence that the group regressed throughout the year or lost productivity when new members joined, which was unexpected* [54].

Communication between the WG and the rest of the organization regarding the OPR is important and can increase interdepartmental understanding, help to prevent resistance to change and enhance buy-in. Moreover, reaching out to the whole organization increases the number of voices that are heard and taken into account. H Waterman and J Grabham [55] provide an example:

The action research group was not insular in that it integrated closely with hospital management systems, for example, senior nurse and general management meetings. Some people who attended the action research meetings, also attended these other meetings, and so ideas and actions from one influenced the other. This ‘spreading and enveloping’ of understanding of issues from all perspectives became vital in the acceptance and inauguration of change.

Second, relationships characterised by mutual trust, respect and support, develop over time, and are important to the success of the OPR. AJ Beringer and ME Fletcher [56] write: “*The single most important indicator of full achievement of outcomes was that the work group members developed mutually supportive and trusting relationships between themselves and with the facilitator. Where these relationships did not develop, this impeded achievement.”* Strategies for nurturing relationships include recognizing partners’ contributions and providing positive reinforcement. One author team suggests: “the value of commending the partnership and acknowledging the productivity of the collaboration cannot be underestimated as a means reinvigorating the relationship and sustaining the collaboration” [34, 57].

Third, WG members’ commitment can lead to more active involvement and the sustainability of changes as illustrated by the following:

The Admiral Nurses’ commitment to stay with the project, organizational commitment to embed the framework in their practice development strategy and support from service managers contributed to the success of the development and implementation of the Admiral Nurses’ Competency Framework. The project commissioners are continuing their support for the framework by explicitly linking it with work on standards of care, new job descriptions and, importantly, through the role of the Consultant Admiral Nurse [58].

Fourth, the collective reflection that occurs during WG meetings seems particularly important. Specifically, collectively reflecting on the OPR and about professional practices stimulates personal reflection and objectivity and supports increased confidence, skills, and insights among WG members. This leads to modifications or adjustments to the OPR and the identification of new or additional issues to pursue via OPR.

*The opportunity for the [Falls Action Research Group] members to meet with their colleagues, from within their own facility and those from another [Residential Aged Care Facility], fostered the development of new understandings about their respective workplaces and the conditions that shaped their practice. As such, the [Falls Action Research Group] members became more familiar with the contributions their colleagues, from other occupational groups, made to resident care* [38].

*Participation in the inquiry by health professionals was important as it fostered cooperation among clinicians and shared decision-making at different levels of interaction. (….) Doctors, pharmacists, and nurses had an opportunity to work closely together on the common goal of implementing change to medication management. In the process, team members also gained a deeper understanding of effective and safe prescribing practices* [59].

### Extra benefits

The process-outcome sequences above lead to extra benefits for individual stakeholders and the organization as a whole. Regarding stakeholders, the awareness, understanding, and general knowledge they gain about research, their work, and their colleagues’ work has many benefits. For example, they experience a sense of achievement, their clinical confidence, skills, and care practices improve, and they develop a drive to do research. In one study, “data had a direct impact on staff serving to broaden understanding of patients’ views on equipment and preoperative education, and it helped monitor the changes that were being put into place” [43].

Attitude changes also occur through the OPR process and lead to practice improvements and other benefits.

During the data interpretation activities, the physicians moved from a classical medical orientation of the individual as the unit of analysis to examining disease patterns in the population. The physicians also began to move from an exclusively curative orientation to disease to a more preventive one [47].

These authors discuss that when partners “saw study results in the form of charts and tables, their level of enthusiasm rose markedly, and they began to participate actively in data interpretation, and to understand how the study could be helpful to them in operating the clinical or preventive sectors of their facility.” Others report that the confidence and sense of ownership organization stakeholders gain from the process increase their autonomy, enthusiasm and responsibility for the work, and empowers them to effect change.

The new behaviours, practices, and skills developed through the OPR also contribute to extra benefits. As S Lauri [60] writes:

*In the opinions of the public health nurses, the conscious implementation of the action model also produced beneficial effects on their work as a whole. Most nurses began to pay more attention to the various areas of child development, and to explore the needs of the child more extensively as the basis of guidance. The objectives and programmes, in the opinion of the public health nurses, gave a direction to and a foundation for the guidance and counselling.*

Regarding the organization, OPR that takes place in a part of a health organisation (e.g., hospital ward) can ultimately affect other parts of the organisation, or beyond. Some studies lead to additional OPR or initiatives as the following OPR summary excerpt illustrates.

As a result of the hospital study, the health education staff already plan to focus on alcohol abuse in those communities which seem to have an elevated problem. Tuberculosis has emerged as a second area of outreach program development. The Foundation’s board of directors has used the hospital data in a fundraising effort for a community education and control program. The hospital’s medical director planned to use the data to identify the priority villages for the tuberculosis program [47].

The OPR process may also expose or raise awareness for additional issues the organization may subsequently address. Two excerpts provide examples:

Using the suggestions for change that had been made by patients, carers and healthcare professionals, eight changes to practice were identified and it was agreed who would be responsible for their implementation. The changes are now being re-evaluated providing the opportunity for healthcare professionals within the colorectal unit, and a new group of patients and carers, to comment on the effects and effectiveness of the changes made thus far [61].

After the manual and new forms had been used for some time, the nurses thought that there were still inefficiencies in the system. Consequently, further phases of the action research project were designed to improve the patient appointment booking and staff allocation systems [42].

### Four ideal-types of OPR

The third and final objective of this synthesis was to identify ideal types of OPR. We found that the 83 studies reviewed can be grouped into four such ideal-types, as presented in Table 1. Additional file 3 presents the 83 summaries organised by the four ideal-types. Select OPR summaries illustrating the ideal types are presented in Table 2 as follows. The most basic type consists of OPR processes that contribute to achieving intended outcomes. This is depicted in a study by Lucas et al. [36, 37]. This first OPR ideal type can lead to random benefits unrelated to the study objectives (OPR ideal type 2). The summary of an OPR by Barker & Barker [62] illustrates multiple such benefits. The third ideal type consists of the OPR (or part of it) being replicated elsewhere in the healthcare organization or even outside of it. A study by Boniface et al. [63] illustrates an OPR in one unit spreading county-wide. In the fourth ideal type, OPR processes contribute to achieving intended outcomes and also to generating, or initiating, new activities or new OPR. The OPR summary of a study by P O’Connor, RR Franklin and CH Behrhorst [47] mentions a variety of new activities that were initiated as a result of the OPR. Any combination of these four ideal types is possible. Some studies in our sample illustrate two, three or all four of the ideal types. For example, a combination of ideal types 2 and 4 is represented in the summary of the dissertation by Heyns [64] (see additional file 3, p. 111). The summary of the study by Sorensen and Haugbolle [65] illustrates a combination of ideal types 1, 2, and 3. (see additional file 3, p. 106). The summary of the study by MS Fagermoen, GA Hamilton, B Svendsen and H Hjellup [66] illustrates an OPR representing all 4 ideal types (Table 2).

**Table 1:** Four ideal types of OPR

The Four Ideal Types of OPR: 1, 1 + 2, 1 + 3, and 1 + 4	
1. OPR processes contribute to achieving intended outcomes (basic OPR)	2. …and ‘random sparks’
Successful OPR focuses on a vested interest of organization stakeholders, be it an interpersonal or organizational one. Through ongoing discussion and critical reflection, a Working Group of academic and organization stakeholders reach consensus over time, regarding this focus, together with all other research-related decisions. Working Groups are often multidisciplinary and may include stakeholders from one or multiple organizations with a common interest. Arguments may occur throughout the process, but a structured and supportive environment helps to work through them. Likewise, valuing input, acknowledging and celebrating contributions and outputs, positive attitude, and fostering motivation, teamwork and trust are important for achieving a shared vision for the OPR and reaching objective(s). Whether the technical work of the analyses is completed by the academic stakeholders or the whole Working Group, communicating preliminary results is important to develop the commitment and motivation of the organisation stakeholders. Moreover, research results help organization stakeholders to see how research can be helpful to them for their health care practice and become a basis for decision making. Preliminary results may include such things are charts, graphs, and fieldwork summaries. Notably, care must be taken to use the right language when communicating research results to assuage potential worries about understanding them. Final results are communicated by the WG to the rest of the organisation and beyond, as needed. This helps to engage additional stakeholders in the OPR. Overall, the OPR processes (ongoing discussion, consensus seeking, data analysis and interpretation of results, decision making, and problem solving) leads to the Working Group achieving its OPR objectives.	Throughout an OPR endeavour, Working Group members learn from one another in myriad ways (e.g., research knowledge and skills, organization or professional constraints, professional knowledge and skills, service users’ and other professionals’ experiences) and some take their learning forward and, for example, enrol in graduate studies or use their new talents in other contexts. Organization stakeholders develop a stronger awareness of contextual issues and concerns in their workplace, gaps in their own professional knowledge and skills, and experience changes in their attitudes toward one another and their practice. Communication, team work, and staff morale improve, and staff turnover decreases. Further, professionals experience increased clinical confidence, empowerment, and job satisfaction. All stakeholders’ perspective of collaboration evolves and the OPR partnership and stakeholder relationships come to be viewed as valuable outcomes, and this, even when stakeholders’ relationships are characterised by tension and mistrust at the outset.
	3. …and the replication of intended outcomes
	Processes used in the OPR become new practices that are maintained. For instance, communication means, such as log books and monthly meetings, are taken up by the whole organization and become regular practice. Additionally, practice changes resulting from the OPR (e.g., interventions, education packages for service users, professional continuing education activities) may be taken up by the whole organization and beyond, to a whole health care services territory or country.
	4. … and the initiation of new activities or new OPR
	Ultimately, additional priorities are identified leading to spin off projects or additional OPR. Sometimes, other organizations aware of the OPR, request OPR facilitation help from the academic stakeholders to address a practice change need in their own milieu.

**Table 2:** OPR summaries illustrating each of the four ideal-types of OPR and their combination

Illustration of OPR ideal type 1: Basic OPR
This 10-month project sought to examine the process of change when developing a preparation programme for patients awaiting Total Knee Replacement (TKR) Surgery in an outer London acute NHS hospital. The researcher initiated and facilitated the project which involved ‘back office’ activities of organisation and encouragement. A Project Management Group (PMG) was established consisting of orthopaedic consultants, nursing staff, physiotherapists, occupational therapists, managers and service, users who were patients who had had Total Knee Replacement surgery at the project site, and the university researcher who was a nurse practitioner within the organisation. Nine monthly PMG meetings held between January and October, with the aims of planning and reviewing the action cycles related to the development of the Knee Clinic and information booklet, and reflection on the progress of the project, including the change process. The researcher took notes during PMG meetings which were distributed to PMG members for checking and correction. PMG members were involved in the action cycles to varying degrees. They worked within the meetings to plan, discuss, analyse and refine the test cycles. They decided which test cycles should continue and which should not be pursued. They participated in the test cycles themselves in various roles including data collection, participation in the Knee Clinic, and administrative tasks. Ultimately, the PMG developed into an effective team, demonstrating the behaviours of good communication and adaptability. Some of the PMG members believed the project was well-managed and that this was a factor in its success. Some staff did not have high expectations of the project but nevertheless participated. It appeared that this participation modified their behaviour in that they continued to provide support to the Knee Clinic after the project ended. For the service users the project provided the environment for them to share and use their experiences of TKR surgery with staff and other patients. They helped to shape the direction of the project and changed the environment through the decision to set up a service user group for others to share their experiences of surgery after the project ended.
Illustration of OPR ideal type 2: Sparks
In 1989 a three-year study began in a substance abuse inpatient unit in a large university teaching hospital in the UK, to generate a description of the substance abuse inpatient program, define and prioritize target areas for change, implement and evaluate change efforts, and provide an opportunity for staff participation and input into the change process. The nurse, medical, and unit directors, and other key staff members (e.g., admitting nurse) formed the team bringing nursing, medical, and psychosocial staff members’ perspectives to meetings. Other staff members volunteered to form various subcommittees that developed and implemented changes (e.g., revision of criteria and procedures for monitoring patient progress in treatment, provision of written policies addressing major issues). All action followed a developmental process in which committees circulated drafts for staff feedback, thereby insuring that staff members were informed and invited to participate in all change efforts. The unit director’s role was that of facilitator, providing encouragement, process monitoring, and feedback. The director relied heavily on group facilitation skills to achieve consensus among staff members. However, this consensus seeking did not occur overnight and actually involved several months of discussions. Through the group process, opinions were voiced resulting in all staff members supporting clinically sound changes that were consistent with the unit goals and philosophy. Changes were assessed by surveys and results were provided to the inservice staff to plan and implement adjustments and, then, re-assess. Administrators’ support was readily forthcoming by including some in the action research process and by keeping others informed through the distribution of survey forms and committee and evaluation reports. The use of the action research model employing staff participation increased the effectiveness of this multidisciplinary inpatient unit. Benefits include: a) an observed increase in staff morale, b) improved staff relations (e.g., good-natured teasing and humor compared to sarcasm and blaming readily observed in meetings prior to the project), c) lower staff turnover, d) more open intradisciplinary and interdisciplinary communication (e.g., staff members now openly support each other, ask for assistance from staff members in other disciplines, and collaborate on problem solving), e) new skills (e.g., team problem identification, decision making, cooperation, leadership), and f) staff appear willing to take more risks in making suggestions, confronting issues, and encouraging and supporting others.
Illustration of OPR ideal type 3: Replication
The aim of this study was to embed the theoretical tenets of the Canadian Model of Occupational Performance and its structures in a way that was appropriate to, and would be used by, all staff within an integrated health and social care setting. Initially enthusiasts were called upon to work in the localities and join a short-term steering group. This small group of self-selecting members of the service and the university lecturer, soon grew to include representatives from all geographical areas and services within the trust (*n* = 16–20). It was non-hierarchical and disseminated the notes of its meetings to the whole service through individual ‘champions’ of the model’s implementation. Despite the attempt to include all staff in the action phases of the research (led by their own representative on the action research steering group), the steering group was a large group and, not all the members were present at all the reflection cycles, but they were crucial to the research’s action phases. The steering group recognised their supervisory and influential role but did not want staff to feel the model was being imposed upon them. The group discussed what resources were required to assist staff confidence and keep the momentum going. Given staff criticisms of communication and dissemination of up to date information the steering group recognised the need to engage both occupational therapists and the managers in the different organisations, and that management and senior occupational therapists needed to support and help maintain enthusiasm for the model within their teams. Thus issues, concerns and good practice were brought to the steering group meetings, and over time tools and materials were developed to help staff in the sharing of good work and solutions to issues and concerns. The collaborative way of carrying out the research ensured the workability of the action. For instance, the steering group member who had previously experienced the dilution of the model’s theory by its paperwork being implemented too early influenced the steering group to delay paperwork implementation. At the same time, other staff members were clamouring for its creation. The result following was that staff in their own settings began to create their own paperwork, which was then brought back to the steering group for further reflection and consequent action. The assessment and planning paperwork that has evolved through this process is now almost countywide, applicable to most areas, firmly embedded in the theory of the model. Another example concerns training. Champions in the acute hospitals produced a training package on the model’s theory and use and the steering group became aware that other areas were keen to use this or a similar package and recognised that the training package was a good way of a team working together to strengthen understanding of the model, share how it could be practically adopted in individual clinical areas and address any issues or concerns. The steering group realised this sharing of knowledge would not have happened if the information had not been taken to a group steering the implementation of the model. The steering group was critical in guiding the model’s implementation, in sustaining motivation and energy across the service, and for communicating information across a wide staff group on an ongoing basis. Many heated discussions occurred, and all members of the group found that their thinking about occupational therapy practice developed and changed. The group has continued to have an important role in making decisions and recognising when staff needed re-energising. The process so far has taken 4 years and is ongoing.
Illustration of OPR Ideal-Type 4: Initiation
When this project began, although the local staff were helpful, they did not envision how the study would be useful and they went along with the initial steps of data collection and analysis passively. One of the major tasks in data analysis was the regrouping of individual diagnoses into manageable categories. Through repeated discussions, among groups composed of Guatemalan and Tulane physicians and epidemiologists, consensus was achieved in developing clinically and conceptually meaningful diagnostic groups. After data processing had been completed, a series of two meetings were held in Guatemala for interpretation of the information generated. The Tulane staff had prepared charts and graphs of the results on a large drawing pad. When staff saw the graphs and tables, the level of enthusiasm rose markedly. They began to participate actively in data interpretation, better understand what Tulane staff were doing, what the results would look like, and how the study could be helpful to them in operating the clinical or preventive sectors of their facility. Interpretations of the data were developed primarily by the three hospital staff physicians through group discussions. They frequently argued about the results, but usually they eventually achieved group consensus regarding their interpretations. By creating a structured and supportive environment for data interpretation, the study reduced their fear. As they became more familiar with the charts and graphs, they began to look at data as a basis for decision making. Within several months of the completion of the data analysis, the findings were being used to identify areas of research and to improve health education and outreach programs. Thus, this project provided a learning experience that afforded an opportunity to become familiar with how data can be useful. The second educational outcome of the joint study was the emergence of a stronger awareness of public health problems. During the data interpretation activities, the physicians moved from a classical medical orientation of the individual as the unit of analysis to examining disease patterns in the population. The physicians also began to move from an exclusively curative orientation to disease to a more preventive one. As a result of the hospital study, the health education staff already plan to focus on alcohol abuse in those communities which seem to have an elevated problem. Tuberculosis has emerged as a second area of outreach program development. The Foundation’s board of directors has used the hospital data in a fundraising effort for a community education and control program. The hospital’s medical director planned to use the data to identify the priority villages for the tuberculosis program. The process of interpreting the findings highlighted a need for larger population based epidemiologic studies to examine relationships between sociodemographic characteristics, cultural beliefs and health practices. To assess the impact of community participation in water projects, the extension staff are now collecting baseline data through a “community diagnosis” instrument. The staff are also participating in a second record study. Preliminary discussions about the establishment of an information system which could be used for program monitoring and impact assessment are also underway. In Summary, the authors believe that the study had two major impacts: (1) the hospital physicians developed a stronger data orientation in studying hospital policies and services and (2) they gained an increased awareness of public health issues.
Illustration of a Combination of the Four Ideal-Types
The purpose of this part of the project was to improve patient information before and at admission for trans-urethral resection and to explore the effect of the changes in the information practices as perceived by the patients. The highly supportive head-nurse selected nine enthusiastic nurses judged to have the necessary professional background and interest to work on the project. Two work-groups were formed, each group a mix of experience and expertise. The nurses identified the problem to be solved and were active participants in the process of change as equal partners with the researcher who had the role of facilitator who used a non-threatening, supportive, and accepting mentoring style and gave credit, guided and advised throughout. The researcher was responsible for the agenda and the minutes from project meetings. All met frequently to collectively discuss the work of each group. They developed a welcome brochure the use of which for all patients admitted to the ward is now regular practice, and other brochures that are sent patients when they receive their date for admission, also now regular practice. Indeed, admission of patients by one nurse each day is now a well-established practice with benefits for all patients, not only the trans-urethral resection -patients. Additionally, guidelines were necessary to ensure that all patients got a certain amount of information at discharge. To evaluate the changes, given no adequate instrument was found, the researchers worked with the nurses to develop an instrument, reaching consensus on topic, readability (literacy level), relevance, and ease of use for the patient. Guidelines for administering the data-collection were established and nurse was designated to do this. The hospital financed a course in SPSS for this nurse, who then was able to participate in developing codebooks and to carry out data-entry. The pace of the study was slower than anticipated given a lower than usual admittance rate of trans-urethral resection patients. This affected the implementation that had been planned to coincide with the merger between the project ward and another urology clinic. The issue of ownership was an important concern. By the end of 2000 most nurses on the ward had not been part of the processes the year before. They received information informally by the nurses closely involved in the project and formally by the nursing professor who met with smaller groups of staff to inform and include them in the ongoing processes. Positive outcomes have resulted for patients and staff through the project. The new brochures improved the patient information, and patients valued the nurses’ interaction and approach, and appreciated the correspondence between the information in the brochures and what went on while in the hospital. Re-designing the brochures benefited staff as well. Structuring the admission talk created a clearer, concise and consistent approach for imparting information. Moreover, as the discussions about the discharge talk evolved, the nurses recognised other areas that needed attention. They identified a need for standardisation of the nurses’ talk with patients on admission and subsequently developed guidelines for this event.

## Discussion

The qualitative synthesis of 83 co-construction OPR studies suggested eight key OPR processes, four that follow the sequence of the research endeavour (form a working group and hold meetings, collectively determine research objectives, collectively analyse data, and collectively interpret results and decide how to use them) and four that can be observed throughout an OPR (communication, relationships development, commitment, and collective reflection). Extra benefits resulting from these key OPR processes occur at the individual and organizational levels.

**Fig. 1:**
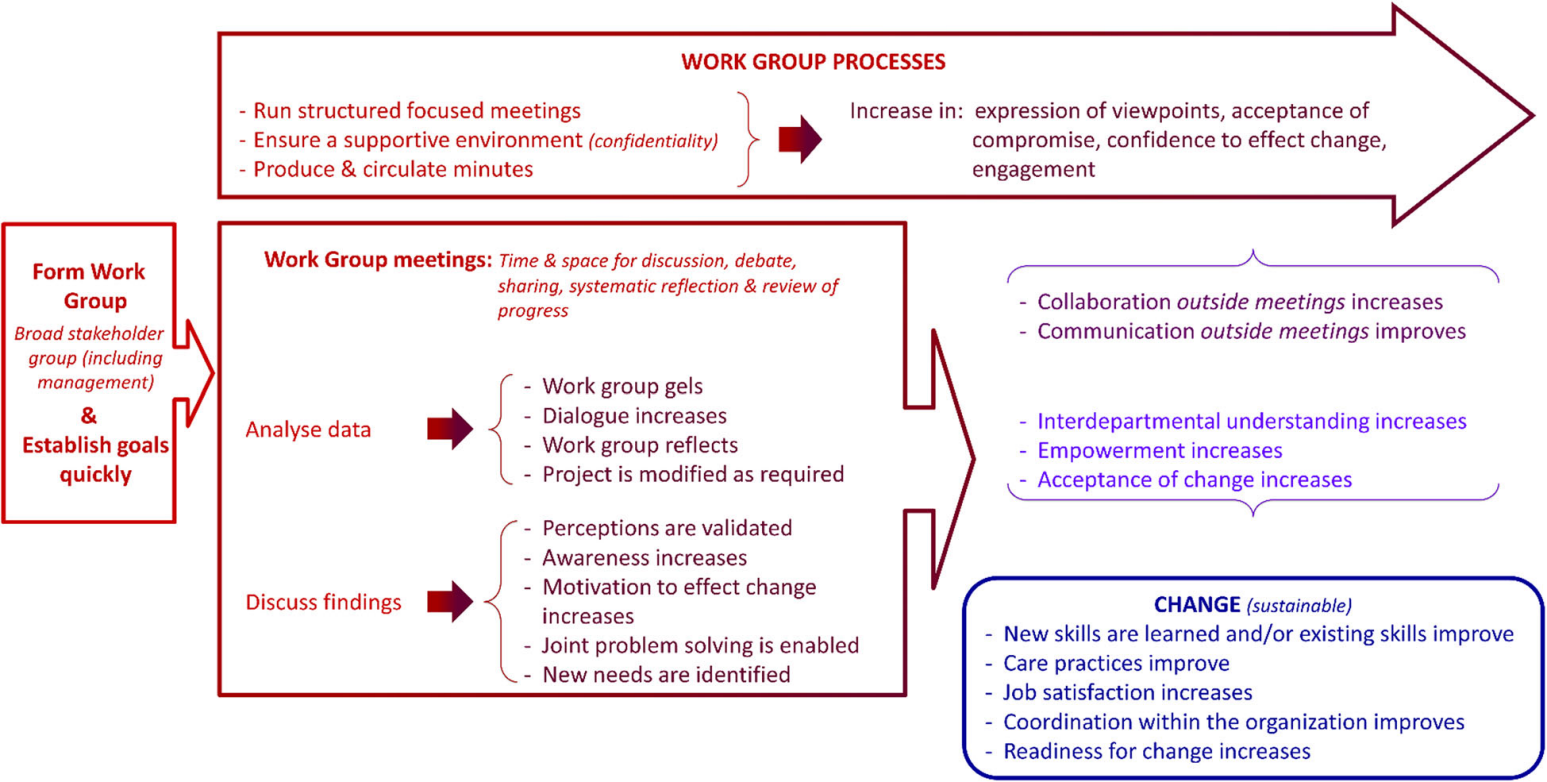
The OPR model – Iterative processes and outcomes of OPR

Together, these key processes and outcomes of OPR can be interpreted through a model for how to conduct an OPR to achieve extra benefits (Fig. 1). This conceptual model suggests that through regular, structured WG meetings stakeholders can voice their varied experiences, ideas for change, fears and other feelings. The WG should assemble a broad variety of stakeholders (practitioners, patients, managers, etc.) and provide a supportive environment with the promise of confidentiality. WG meeting processes include identifying needs and formulating study objectives, collectively analysing data, and discussing results and how to act on them. Circulating meeting summaries between meetings is important for deliberations, to correct misunderstandings, and to help engage WG members who are unable to attend some meetings. During meetings, WG members learn from one another, gain awareness of constraints to addressing the OPR objectives, gain confidence (which in turn increases commitment to continue the research and to take responsibility for decisions and actions) through systematic reflection on the OPR. This is in line with H Waterman, D Tillen, R Dickson and K de Koning [3] who found that sharing ideas is part of learning that takes place through the process, which in turn increases participation in the research. Discussing OPR results within the WG is valuable in that results often validate perceptions and raise awareness. Discussing results also increases WG members’ understanding of how to use research findings to inform changes, enables joint problem solving, increases members’ motivation to make change, and helps them identify additional needs. In their review, G Munten, J Van Den Bogaard, K Cox, H Garretsen and I Bongers [15] identified communication and feedback of results strategies as common. Our work goes a step farther in illustrating the potential consequences of such strategies.

The OPR processes in which the WG engages during meetings contribute to improved communications and coordination both within the WG and between the WG and their healthcare organization, which in turn increases the organization members’ acceptance of change. Ultimately, WG members improve or develop new skills (supports empowerment), and teamwork, mutual understanding and job satisfaction are increased or improved. Readiness for change increases and improved care and sustainable changes ensue. Importantly, the changes pave the way for subsequent changes. While H Waterman, D Tillen, R Dickson and K de Koning [3] cite many of these results as benefits of participation, our synthesis clearly suggests it is the WG meetings that contribute to these outcomes because they provide invaluable time and space for WG members to present, discuss, debate, and reflect on various identified needs. They are also a space to reach consensus or to accept compromise. Similarly, in their review, G Munten, J Van Den Bogaard, K Cox, H Garretsen and I Bongers [15] found that meetings in small or large groups was the strategy most often cited in included studies. However, they found the nature of the interactions during these meetings was not sufficiently described. They underscore the need for authors to provide detailed descriptions to help understand the ‘black box’ of this research approach in order to close the research-practice gap. We have delved into the ‘mess’ [21] of OPR combining the experiential, propositional and practical OPR knowledge of our diverse team. Examining authors’ reports of their OPR activities and consequences (process-outcome linkages) in their OPR studies helped us to begin to unpack this black box.

### Applicability and practical implications of the review findings

Previous reviews, whether about OPR or other types of participatory research, have not described how to conduct this type of research to the same degree, nor have they illustrated how participatory processes contribute to outcomes. In phase-1 of this systematic review [9], we found five types of extra benefits related to a framework of capacity building: leadership, general workforce development, group benefits, broad systemic developments or changes, academic researchers’ capacity. With this second phase of the review, we have been able to go beyond this description of extra benefits to explain how to achieve them. The conceptual model (Fig. 1), can be applied widely to guide the conduct and assessment of OPR. While based on OPR with healthcare organizations, the processes and outcomes illustrated in the model may be applicable to any organization. Since we focussed on OPR processes linked to OPR outcomes, the data analysed had an inherent chronology (narrative causation) [67]: processes *lead to* outcomes. This is visible in the final coding trees which consist of process-outcome sequences, each with inductively derived codes that depict passage of time (additional file 4). It is important to note the iterative nature of the processes and outcomes. In practice, OPR is not as linear as the model suggests. Throughout an OPR project, the process-outcome sequences repeat and overlap, and there are feedback loops between them. However, in keeping with the principle of parsimony, our model provides a simple illustration of the overall flow of theses sequences and explains how to conduct OPR to achieve extra benefits. The parsimonious representation is also in line with the philosophy of OPR to produce useful results. Finally, this simplified conceptualisation is an actionable message, which some would argue is necessary for its uptake and use [68, 69]. The model is thus a significant contribution of our work.

The four ideal types illustrate features of learning organisations such as open systems thinking, building individuals’ capacities, sharing knowledge, and learning collectively [70]. Our synthesis illustrates that while OPR is a means to achieve study objectives (basic OPR), it may also lead to any combination of (a) outcomes unrelated to the study, or ‘random sparks’ as described by H Atlan [71] (OPR ideal type 2), (b) replication of outcomes (OPR ideal-type 3), or (c) initiation of new OPR or activities (OPR ideal type 4) [72]. Replication was also a finding of one OPR review that reported effects beyond the location in 13% of included studies (*n* = 4) [3]. Moreover, this is inline with the capacity building and sustainability literature [73, 74] which suggests change is more likely to endure if it spreads or multiplies. Thus, OPR as we have operationalised it in our model, can be a means for academic and organization stakeholder to co-create lasting change. Our findings are in line with a CBPR review [29] found that community-based participatory research leads to new unanticipated projects and activity, can be conceived of as initiation, as per JC Greene, VJ Caracelli and WF Graham [72]. While previous works provide typologies of non-academic stakeholder participation [3, 5, 14], going forward, it may be relevant to categorise studies as per the four ideal-types of OPR.

### Limitations and strengths and transferability of the evidence

This qualitative synthesis is not entirely based on empirical results of included studies. In many cases, the data were from the discussion section of the reviewed studies and are, thus, the authors’ reflections on their practical experience with OPR. Given that our analysis is therefore a meta-reflection, it is possible that our results underscore the assumptions and beliefs of the authors of the publications included in this review. Future research should test our conceptual model and ideal types of OPR. However, our team consists of eleven OPR practitioners and scholars with varied frames of reference, and the critical input of each throughout our review helped us to challenge our own assumptions about the data and our analyses; thereby, adding to the trustworthiness of our results. Moreover, the large number of studies included in this review, the multiple descriptions of OPR processes-outcome sequences in these studies, and the data saturation lends credibility to our results.

Although predominantly influenced by nursing studies in hospital settings (given this is the most common type of OPR to date), this review is more comprehensive than previous ones which focussed on OPR in UK health settings [3] in nursing [14], on implementation of evidence-based practice in nursing [15] or in adult intensive care units [16]. We included a variety of types of health OPR in diverse types of healthcare organizations. Moreover, this review overcomes issues previously reported regarding attributing organization members’ participation in research to particular outcomes [3, 14, 15] since we selected studies based on a precise definition of OPR and excluded those that did not make an explicit link between OPR processes and outcomes [3, 14, 15].

Similar to all literature reviews, our work is limited by publication bias. As found in previous reviews [15, 16], the challenges of OPR (i.e., negative outcomes), and means to mitigate with them (processes), were rarely reported in the included studies and are, thus, not covered in this article. Our practical experience, however, suggests that OPR can be quite challenging. A primary study to identify challenges OPR stakeholders face and describe how they deal with them would be a valuable contribution to the literature.

## Conclusion

With this review, we have broadened the understanding of OPR and the value of this research approach by identifying and illustrating sequences of OPR process-outcome sequences. Specifically, our results suggest that OPR stakeholders form a WG and hold meetings where they collectively determine the research objectives, analyse the data and interpret the results and decide how to use them. Throughout these research phases, communication, relationships development, commitment, and collective reflection should be maintained. These processes contribute to knowledge, attitude and behaviour changes in the stakeholders and the healthcare organization. Since our analysis is based only on OPR processes that were explicitly linked with OPR outcomes, we assert that these are the key processes to follow when conducting OPR. Moreover, as per the four ideal types of OPR, we submit that when these processes are followed, OPR teams will achieve their objectives, and may also achieve one or more extra benefits in the form of sparks, replication or initiation. Overall, this review provides operational guidance to help OPR stakeholders collaboratively address organizational issues and achieve desired outcomes and more.

## Additional files

Additional file 1: Search strategies. This documents provides the detailed search strategies for each bibliographic data base we searched. (PDF 195 kb)

Additional file 2: Data extraction form. This Excel form used for all 83 studies included in this review. (XLSX 12 kb)

Additional file 3: Eighty-three Organizational Participatory Research Summaries. This document includes the 83 OPR summaries organized according to four ideal-types of OPR. (PDF 873 kb)

Additional file 4: Coding trees. This document includes screenshots of the coding trees with the number of OPR summaries (sources) per code and number of codes per summary (references). (PDF 199 kb)

## Abbreviations

OPR: Organizational Participatory Research
WG: Working group
