# Supplementary material for: A systematic mixed studies review on Organizational Participatory Research: towards operational guidance
Source: BMC Health Serv Res. 2018 Dec 22;18:992. doi: 10.1186/s12913-018-3775-5 (PMC6421946; doi:10.1186/s12913-018-3775-5)
Supplement: Supplementary file 1 — Search strategies. This documents provides the detailed search strategies for each bibliographic data base we searched. (PDF 195 kb) [file 12913_2018_3775_MOESM1_ESM.pdf]

Research Question	Parameters to address		
In this systematic **mixed studies** review, we will synthesize results of studies that use **participatory research** aiming to **change practice** with and within **health organizations** offering healthcare, health promotion, and health education services and products, such as (a) healthcare delivery workplaces, e.g., hospital or primary care clinic, (b) community health facilities, e.g., fitness centre, (c) health professional organizations, e.g., College of Family Physicians of Canada, (d) public health agencies, e.g., Health Canada, (e) health non-governmental organizations, e.g., Doctors of the World, and (f) disease and patient groups, e.g., the Heart and Stroke Foundation.	**Medicine**<br>_x_ Cochrane (CSDR, CENTRAL, etc)<br>_x_ MEDLINE on PubMed and/or OvidSP<br>___ Global Health<br>_x_ EMBASE (biomedical)<br>_x_ CINAHL (allied health, e.g., nursing)<br>___ African Index Medicus (AIM)<br>___ LILACS (South American/Caribbean)<br>___ POPLine (Reproductive health)	**Psychology**<br>_x__ PsycINFO<br><br>**Geography (Human)**<br>___ PAIS International<br>___ GEOBASE (part of Scopus)<br><br>**Economics**<br>___ Econlit<br>___ PAIS International	**Social Sciences**<br>___ Science Citation Index (part of Web of Science)<br>___ Social Sciences Citation Index (part of Web of Science)<br>___ IBSS (International Bibliography of the Social Sciences)<br>_x__ Social Work Abstracts on Ovid SP<br>___ Sociological Abstracts<br>___ Campbell Collaboration<br><br>**Business**<br>_x_ Business Source Complete on EBSCO
	**Grey Literature [websites, conference proceedings, etc.]**<br>___IGO Search<br>___OECD iLibrary	_x_ English & French<br>___ All languages<br>___ Years:	**Multidisciplinary**<br>___ Web of Knowledge (includes Web of Science)<br>_x_Scopus (includes Embase)<br>_x_Google Scholar

Database: MEDLINE on PubMed

	Concept: Action research	Concept: Organizational setting (facilities)	Concept: Organizational involvement / improvement
Thesaurus Terms / Subheadings	community based participatory research[MeSH Terms]<br>(community institutional relation[MeSH Terms] AND (research[Title] OR research[MeSH Terms])	residential facilities[MeSH Terms]<br>rehabilitation centers[MeSH Terms]<br>physicians offices[MeSH Terms]<br>pharmacies[MeSH Terms]<br>nurseries[MeSH Terms]<br>hospitals[MeSH Terms]<br>hospital units[MeSH Terms]<br>fitness centers[MeSH Terms]<br>dental facilities[MeSH Terms]<br>birthing centers[MeSH Terms]<br>ambulatory care facilities[MeSH Terms]<br>academic medical centers[MeSH Terms]<br>health facilities[MeSH:noexp]<br>organizations[Mesh:noexp]<br>organizations, Nonprofit[Mesh:noexp]	organizational innovation[MeSH Terms]
Textwords	"action research" [All Fields]<br>"participatory research" [All Fields]<br>"participative research" [All Fields]<br>"collaborative inquiry" [All Fields]<br>"collaborative action" [All Fields]<br>"collaborative partnership" [All Fields]<br>"collaborative partnerships" [All Fields]<br>"participatory rural appraisal" [All Fields]<br>"participatory appraisal" [All Fields]<br>"emancipatory research" [All Fields]<br>"social reconnaissance" [All Fields]<br>"empowerment evaluation" [All Fields]<br>((research[Title]) OR design[Title]) AND participatory[Title]<br>(action[Title]) AND research[Title]<br>CBPR [All Fields]	organisation[Title/Abstract]<br>organisations[Title/Abstract]<br>organisational[Title/Abstract]<br>organization[Title/Abstract]<br>organizations[Title/Abstract]<br>organizational[Title/Abstract]<br>institution[Title/Abstract]<br>institutions[Title/Abstract]<br>institutional[Title/Abstract]<br>hospital[Title/Abstract]<br>hospitals[Title/Abstract]<br>facility[Title/Abstract]<br>facilities[Title/Abstract]<br>unit[Title/Abstract]<br>units[Title/Abstract]<br>ward[Title/Abstract]	((evaluation[Title/Abstract]) OR consultation[Title/Abstract]) AND process[Title/Abstract]<br>((management[Title/Abstract]) OR implementation[Title/Abstract]) AND change[Title/Abstract]<br>((management[Title/Abstract]) OR transformative[Title/Abstract]) AND research[Title/Abstract]<br>"community of practice"[Title/Abstract]<br>"organizational learning"[Title/Abstract]<br>"organisational learning"[Title/Abstract]<br>"transformational change"[Title/Abstract]

	Concept: Action research	Concept: Organizational setting (facilities)	Concept: Organizational involvement / improvement
	"community based participatory research" [All Fields]<br>"community based action research" [All Fields]<br>"participatory evaluation" [All Fields]<br>"community driven research" [All Fields]<br>"action science" [All Fields]<br>"cooperative inquiry" [All Fields]<br>"appreciative inquiry" [All Fields]<br>"democratic evaluation" [All Fields]<br>"recherche action" [All Fields]	wards[Title/Abstract]<br>clinic[Title/Abstract]<br>clinics[Title/Abstract]<br>pharmacy[Title/Abstract]<br>pharmacies[Title/Abstract]<br>center[Title/Abstract]<br>centers[Title/Abstract]<br>centre[Title/Abstract]<br>centres[Title/Abstract]<br>team[Title/Abstract]<br>teams[Title/Abstract]<br>agency[Title/Abstract]<br>agencies[Title/Abstract]<br>association[Title/Abstract]<br>associations[Title/Abstract]<br>“care trust”[Title/Abstract])<br>“care trusts”[Title/Abstract]<br>“healthcare trust”[Title/Abstract]<br>“healthcare trusts”[Title/Abstract]<br>“service trust”[Title/Abstract]<br>“service trusts”[Title/Abstract]<br>“services trust”[Title/Abstract]<br>“services trusts”[Title/Abstract]<br>“hospital trust”[Title/Abstract]<br>“hospital trusts”[Title/Abstract]	

Database: EMBASE

	Concept: Action research	Concept: Organizational setting (facilities)	Concept: Organizational involvement / improvement
Thesaurus Terms / Subheadings	exp action research/	hospital/<br>community hospital/<br>general hospital/<br>geriatric hospital/<br>exp mental hospital/<br>non profit hospital/<br>pediatric hospital/<br>private hospital/<br>public hospital/<br>exp teaching hospital/<br>health care facility/<br>assisted living facility/<br>cancer center/<br>community mental health center/<br>dental facility/<br>health center/<br>mental health center/<br>nursing home/<br>pain clinic/<br>pharmacy/<br>rehabilitation center/<br>residential home/<br>health care organization/<br>non profit organization/	change management/<br>organization/<br>organizational development/<br>organizational efficiency/<br>exp planning/

Textwords	"action research"<br>"participatory research"<br>"participative research"<br>"collaborative inquiry"<br>“collaborative action”<br>“collaborative partnership?”<br>"participatory rural appraisal"<br>"participatory appraisal"<br>"emancipatory research"<br>"social reconnaissance"<br>"empowerment evaluation"<br>(participatory[title] AND (research[title] OR design[title]))<br>(action[title] AND research[title])<br>“dialectical research”<br>“conscienti#ing research”<br>“participatory learning research”<br>“CBPR”<br>“community-based participatory research”<br>“community-based action research”<br>“participatory evaluation”<br>“participative evaluation”<br>“community-driven research”<br>“community-driven action research”<br>“action science”<br>“community-partnered action research”<br>“cooperative inquiry”<br>“dialectical inquiry”<br>“appreciative inquiry”<br>“decoloni#ing methodologies”<br>“democratic evaluation”<br>“recherche participative”<br>“recherche-action”	organi#ation$.ti,ab.<br>institution$.ti,ab.<br>(hospital or hospitals).ti,ab.<br>(facility or facilities).ti,ab.<br>(unit or units).ti,ab.<br>(ward or wards).ti,ab.<br>(clinic or clinics).ti,ab.<br>(pharmacy or pharmacies).ti,ab.<br>(center or centers).ti,ab.<br>(centre or centres).ti,ab.<br>(team or teams).ti,ab.<br>(agency or agencies).ti,ab.<br>(association or associations).ti,ab.<br>(care trust or care trusts).ti,ab.<br>(healthcare trust or healthcare trusts).ti,ab.<br>(service? trust or service? trusts).ti,ab.<br>(hospital trust or hospital trusts).ti,ab.	((evaluation or consultation or apprais$) and process).ti,ab.<br>((management or implementation) and change).ti,ab.<br>((management or transformative) and research).ti,ab.<br>(community ADJ1 practice).ti,ab.<br>(organi#ational learning).ti,ab.<br>transformational change.ti,ab.

	“recherche collaborative”		

Database: CINAHL

	Concept: Action Research	Concept: Organizational Setting	Concept:
Thesaurus Terms / Subheadings	MH "Action Research"<br><br>(MH” Community-Institutional Relations”<br>AND (TI research OR MH “Research”))	(MH "Emergency Service+")<br>(MH "Midwifery Service+")<br>(MH "Nursing Service")<br>(MH "Obstetric Service")<br>(MH "Occupational Therapy Service")<br>(MH "Outpatient Service")<br>(MH "Pain Clinics")<br>(MH "Pharmacy Service+")<br>(MH "Physical Therapy Service")<br>(MH "Psychiatric Service")<br>(MH "Health Facilities")<br>(MH "Academic Medical Centers")<br>(MH "Alternative Health Facilities")<br>(MH "Ambulatory Care Facilities+")<br>(MH "Community Health Centers")<br>(MH "Dental Facilities+")<br>(MH "Fitness Centers")<br>(MH "Hospital Units+")<br>(MH "Hospitals+")<br>(MH "Practitioner's Office")<br>(MH "Rehabilitation Centers+")<br>(MH "Residential Facilities+")<br>(MH "Rural Health Centers")<br>(MH "Organizations")<br>(MH "Organizations, Nonprofit")<br>(MH "Professional Organizations")	(MH "Organizational Efficiency+")<br>(MH "Organizational Development")<br>(MH "Program Evaluation")
Textwords	"Action research"<br>"participatory research"	organi?ation*<br>institution*	("process" AND ("Evaluation" OR "consultation" OR "apprais*"))

	Concept: Action Research	Concept: Organizational Setting	Concept:
	"participative research"<br>"collaborative inquiry"<br>"collaborative action"<br>"collaborative partnership*"<br>"participatory rural appraisal"<br>"participatory appraisal"<br>"emancipatory research"<br>"social reconnaissance"<br>"empowerment evaluation"<br>TI participatory AND (TI research OR TI design)<br>(TI action AND TI research)<br>"dialectical research"<br>"conscienti?ing research"<br>"participatory learning research"<br>"CBPR"<br>"community-based participatory research"<br>"community-based action research"<br>"participatory evaluation"<br>"participative evaluation"<br>"community-driven research"<br>"community-driven action research"<br>"action science"<br>"community-partnered action research"<br>"cooperative inquiry"<br>"dialectical inquiry"<br>"appreciative inquiry"<br>"decoloni?ing methodologies"<br>"democratic evaluation"<br>"recherche participative"<br>"recherche action"	hospital<br>hospitals<br>facility<br>facilities<br>unit<br>units<br>ward<br>wards<br>clinic<br>clinics<br>pharmacy<br>pharmacies<br>Center<br>Centers<br>Centre<br>Centres<br>team<br>teams<br>agency<br>agencies<br>association<br>associations<br>"care trust”<br>“care trusts"<br>"healthcare trust”<br>“healthcare trusts"<br>"service* trust”<br>“service* trusts"<br>"hospital trust”<br>“hospital trusts"	("change" AND ("management" OR "implementation"))<br>(("management" OR "transformative") AND "research")<br>"community of practice"<br>"organi?ational learning"<br>"transformational change"

	Concept: Action Research	Concept: Organizational Setting	Concept:
	"recherche collaborative"		

Database: PsycINFO

	Concept: Action research	Concept: Organizational setting (facilities)	Concept: Organizational involvement / improvement
Thesaurus Terms / Subheadings	exp Action Research/	treatment facilities/<br>exp clinics/<br>exp community mental health centers/<br>exp hospitals/<br>exp nursing homes/<br>exp residential care institutions/<br>organizations/<br>nonprofit organizations/<br>professional organizations/	exp organizational learning/<br>exp "communities of practice"/<br>exp organizational behavior/<br>exp organizational change/<br>exp organizational characteristics/<br>exp organizational development/<br>exp organizational effectiveness/
Textwords	"action research"<br>"participatory research"<br>"participative research"<br>"collaborative inquiry"<br>“collaborative action”<br>“collaborative partnership?”<br>"participatory rural appraisal"<br>"participatory appraisal"<br>"emancipatory research"<br>"social reconnaissance"<br>"empowerment evaluation"<br>((research[title] OR design[title]) AND participatory[title])<br>(action[title] AND research[title])<br>“dialectical research”<br>“conscienti#ing research”<br>“participatory learning research”	organi#ation$.ti,ab.<br>institution$.ti,ab.<br>(hospital or hospitals).ti,ab.<br>(facility or facilities).ti,ab.<br>(unit or units).ti,ab.<br>(ward or wards).ti,ab.<br>(clinic or clinics).ti,ab.<br>(pharmacy or pharmacies).ti,ab.<br>(center or centers).ti,ab.<br>(centre or centres).ti,ab.<br>(team or teams).ti,ab.<br>(agency or agencies).ti,ab.<br>(association or associations).ti,ab.<br>(care trust or care trusts).ti,ab.<br>(healthcare trust or healthcare trusts).ti,ab.<br>(service? trust or service? trusts).ti,ab.<br>(hospital trust or hospital trusts).ti,ab.	(process[title] AND (evaluation[title] OR consultation[title] OR apprais*[title]))<br>.ti,ab.<br>(change[title] AND (management[title] OR implementation[title]).ti,ab.<br>((management[title] OR transformative[title]) AND research[title]).ti,ab.<br>(community ADJ1 practice).ti,ab.<br>(organi#ational learning).ti,ab.<br>transformational change.ti,ab.

	Concept: Action research	Concept: Organizational setting (facilities)	Concept: Organizational involvement / improvement
	"CBPR"<br>"community-based participatory research"<br>"community-based action research"<br>"participatory evaluation"<br>"participative evaluation"<br>"community-driven research"<br>"community-driven action research"<br>"action science"<br>"community-partnered action research"<br>"cooperative inquiry"<br>"dialectical inquiry"<br>"appreciative inquiry"<br>"decoloni#ing methodologies"<br>"democratic evaluation"<br>"recherche participative"<br>"recherché-action"<br>"recherche collaborative"		

Database: Social Work Abstracts

	Concept: Action research	Concept: Organizational setting (facilities)	Concept: Organizational involvement / improvement
Thesaurus Terms / Subheadings	action research.sh.<br>participatory research.sh.<br>collaborative research.sh.	clinics.sh.<br>hospitals.sh.<br>health centers.sh.<br>health organizations.sh.<br>health care organizations.sh.	
Textwords	"action research"<br>"participatory research"<br>"participative research"<br>"collaborative inquiry"<br>“collaborative action”<br>“collaborative partnership?”<br>"participatory rural appraisal"<br>"participatory appraisal"<br>"emancipatory research"<br>"social reconnaissance"<br>"empowerment evaluation"<br>((research[title] OR design[title]) AND participatory[title])<br>(action[title] AND research[title])<br>“dialectical research”<br>“conscienti#ing research”<br>“participatory learning research”<br>“CBPR”<br>“community-based participatory research”<br>“community-based action research”<br>“participatory evaluation”<br>“participative evaluation”<br>“community-driven research”<br>“community-driven action research”<br>“action science”		

	Concept: Action research	Concept: Organizational setting (facilities)	Concept: Organizational involvement / improvement
	“community-partnered action research”<br>“cooperative inquiry”<br>“dialectical inquiry”<br>“appreciative inquiry”<br>“decoloni#ing methodologies”<br>“democratic evaluation”<br>“recherche participative”<br>“recherché-action”<br>“recherche collaborative”		

Database: Business Source Complete

	Concept: Action research	Concept: Organizational setting (facilities)	Concept: Organizational involvement / improvement
Thesaurus Terms / Subheadings		(DE "HOSPITALS")<br>(DE "CLINICS")<br>(DE "HEALTH facilities"<br>DE "HEALTH maintenance organizations"<br>DE "MEDICAL centers"<br>DE "HEALTH professionals' associations"	DE "ORGANIZATIONAL change" OR DE "ORGANIZATIONAL effectiveness"
Textwords	"Action research"<br>"participatory research"<br>"participative research"<br>"collaborative inquiry"<br>"collaborative action"<br>"collaborative partnership*"<br>"participatory rural appraisal"<br>"participatory appraisal"<br>"emancipatory research"	“health organi?ation*”<br>“healthcare organi?ation*”<br>“health institution*”<br>“healthcare institution”<br>hospital<br>hospitals<br>health facility<br>health facilities<br>health unit	("process" AND ("Evaluation" OR "consultation" OR "apprais*"))<br>("change" AND ("management" OR "implementation"))<br>(("management" OR "transformative") AND "research")<br>"community of practice"<br>"organi?ational learning"<br>"transformational change"

	Concept: Action research	Concept: Organizational setting (facilities)	Concept: Organizational involvement / improvement
	"social reconnaissance"<br>"empowerment evaluation"<br>TI participatory AND (TI research OR TI design)<br>(TI action AND TI research) OR<br>"dialectical research"<br>"conscienti?ing research"<br>"participatory learning research"<br>"CBPR"<br>"community-based participatory research"<br>"community-based action research"<br>"participatory evaluation"<br>"participative evaluation"<br>"community-driven research"<br>"community-driven action research"<br>"action science"<br>"community-partnered action research"<br>"cooperative inquiry"<br>"dialectical inquiry"<br>"appreciative inquiry"<br>"decoloni?ing methodologies"<br>"democratic evaluation"<br>"recherche participative"<br>"recherche action"<br>"recherche collaborative"	health units<br>clinic<br>clinics<br>pharmacy<br>pharmacies<br>health Center<br>health Centers<br>health Centre<br>health Centres<br>health agency<br>health agencies<br>health association<br>health associations<br>"healthcare trust”<br>“healthcare trusts"<br>"health service* trust”<br>“health service* trusts"<br>"hospital trust”<br>“hospital trusts"	

Database: Cochrane library search strategy

Search Name:	Full set 20121129
Date Run:	29/11/12 21:12:55.655

ID	Search	Hits
#1	MeSH descriptor: [Community-Based Participatory Research] explode all trees	53
#2	MeSH descriptor: [Community-Institutional Relations] explode all trees	155
#3	MeSH descriptor: [Research] explode all trees	11020
#4	research:ti (Word variations have been searched)	5950
#5	#2 and (#3 or #4)	33
#6	action research:ti,ab,kw	127
#7	participatory research:ti,ab,kw	100
#8	participative research:ti,ab,kw	1
#9	collaborative inquiry:ti,ab,kw	1
#10	collaborative action:ti,ab,kw	1
#11	(collaborative next partnership*):ti,ab,kw	10
#12	participatory rural appraisal:ti,ab,kw	0
#13	participatory appraisal:ti,ab,kw	0
#14	emancipatory research:ti,ab,kw	0
#15	social reconnaissance:ti,ab,kw	0
#16	empowerment evaluation:ti,ab,kw	2
#17	(research:ti or design:ti) and participatory:ti	35
#18	action:ti and research:ti	42
#19	CBPR:ti,ab,kw	21
#20	community based participatory research:ti,ab,kw	87
#21	community based action research:ti,ab,kw	1

#22	participatory evaluation:ti,ab,kw	0
#23	community driven research:ti,ab,kw	0
#24	action science:ti,ab,kw	0
#25	cooperative inquiry:ti,ab,kw	0
#26	appreciative inquiry:ti,ab,kw	3
#27	democratic evaluation:ti,ab,kw	0
#28	(decolono?ing next methodologies):ti,ab,kw	0
#29	(conscienti?ing next research):ti,ab,kw	0
#30	recherche action:ti,ab,kw	2
#31	recherche participative:ti,ab,kw	0
#32	recherche collaborative:ti,ab,kw	0
#33	#1 or #5 or #6 or #7 or #8 or #9 or #10 or #11 or #12 or #13 or #14 or #15 or #16 or #17 or #18 or #19 or #20 or #21 or #22 or #23 or #24 or #25 or #26 or #27 or #28 or #29 or #30 or #31 or #32	292
#34	MeSH descriptor: [Residential Facilities] explode all trees	1125
#35	MeSH descriptor: [Rehabilitation Centers] explode all trees	505
#36	MeSH descriptor: [Physicians' Offices] explode all trees	30
#37	MeSH descriptor: [Pharmacies] explode all trees	59
#38	MeSH descriptor: [Nurseries] explode all trees	37
#39	MeSH descriptor: [Hospitals] explode all trees	2420
#40	MeSH descriptor: [Hospital Units] explode all trees	2554
#41	MeSH descriptor: [Fitness Centers] explode all trees	19
#42	MeSH descriptor: [Dental Facilities] explode all trees	54
#43	MeSH descriptor: [Birthing Centers] explode all trees	19
#44	MeSH descriptor: [Ambulatory Care Facilities] explode all trees	1430
#45	MeSH descriptor: [Academic Medical Centers] explode all trees	1109
#46	MeSH descriptor: [Health Facilities] this term only	34
#47	MeSH descriptor: [Organizations] this term only	8
#48	MeSH descriptor: [Organizations, Nonprofit] this term only	11

#49	organi?ation*:ti,ab	4946
#50	institution*:ti,ab	5489
#51	hospital:ti,ab	36342
#52	facility:ti,ab	2918
#53	unit:ti,ab	19013
#54	ward:ti,ab	2493
#55	clinic:ti,ab	12735
#56	pharmacy:ti,ab	968
#57	center:ti,ab	29530
#58	team:ti,ab	3073
#59	agency:ti,ab	947
#60	association:ti,ab	18689
#61	(care next trust*):ti,ab	52
#62	(healthcare next trust*):ti,ab	6
#63	(service* next trust*):ti,ab	13
#64	(hospital next trust*):ti,ab	24
#65	#34 or #35 or #36 or #37 or #38 or #39 or #40 or #41 or #42 or #43 or #44 or #45 or #46 or #47 or #48 or #49 or #50 or #51 or #52 or #53 or #54 or #55 or #56 or #57 or #58 or #59 or #60 or #61 or #62 or #63 or #64	114273
#66	MeSH descriptor: [Organizational Innovation] this term only	68
#67	(evaluation:ti,ab or consultation:ti,ab) and process:ti,ab	2298
#68	(management:ti,ab or implementation:ti,ab) and change:ti,ab	5781
#69	(management:ti,ab or transformative:ti,ab) and research:ti,ab	2868
#70	community or practice:ti,ab	0
#71	(organi?ational next learning):ti,ab	1
#72	transformational change:ti,ab	0
#73	#66 or #67 or #68 or #69 or #70 or #71 or #72	10096
#74	#33 and (#65 or #73) (Word variations have been searched)	133

Database: PubMed search strategy

Search	Add to builder	Query	Items found
#103	Add	Search **(#58) AND #100** Filters: **French; English**	3336
#102	Add	Search **(#58) AND #100** Filters: **French**	33
#101	Add	Search **(#58) AND #100**	3414
#100	Add	Search **(#91) OR #99**	2753193
#99	Add	Search **((((((#92) OR #93) OR #94) OR #95) OR #96) OR #97) OR #98**	132155
#98	Add	Search **"transformational change"[tiab]**	109
#97	Add	Search **"organizational learning"[tiab] OR "organisational learning"[tiab]**	235
#96	Add	Search **"community of practice"[tiab]**	149
#95	Add	Search **((management[tiab] OR transformative[tiab]) AND research[tiab])**	46386
#94	Add	Search **((management[tiab] OR implementation[tiab]) AND change[tiab])**	31467
#93	Add	Search **((evaluation[tiab] OR consultation[tiab]) AND process[tiab])**	39594
#92	Add	Search **organizational innovation[MeSH Terms]**	21165
#91	Add	Search **((((((((((((((((((((((((((((#59) OR #60) OR #61) OR #62) OR #63) OR #64) OR #65) OR #66) OR #67) OR #68) OR #69) OR #70) OR #71) OR #72) OR #73) OR #75) OR #76) OR #77) OR #78) OR #79) OR #80) OR #81) OR #82) OR #83) OR #84) OR #85) OR #86) OR #87) OR #88) OR #89) OR #90**	2672925
#90	Add	Search **"hospital trust"[tiab] OR "hospital trusts"[tiab]**	449
#89	Add	Search **"service trust"[tiab] OR "service trusts"[tiab] OR "services trust"[tiab] OR "services trusts"[tiab]**	236
#88	Add	Search **"healthcare trust"[tiab] OR "healthcare trusts"[tiab]**	47

#87	Add	Search **"care trust"[tiab] OR "care trusts"[tiab]**	1165
#86	Add	Search **association[tiab] OR associations[tiab]**	690052
#85	Add	Search **agency[tiab] OR agencies[tiab]**	46983
#84	Add	Search **team[tiab] OR teams[tiab]**	78047
#83	Add	Search **center[tiab] OR centers[tiab] OR centre[tiab] OR centres[tiab]**	436102
#82	Add	Search **pharmacy[tiab] OR pharmacies[tiab]**	26218
#81	Add	Search **clinic[tiab] OR clinics[tiab]**	181866
#80	Add	Search **ward[tiab] OR wards[tiab]**	35988
#79	Add	Search **unit[tiab] OR units[tiab]**	399873
#78	Add	Search **facility[tiab] OR facilities[tiab]**	84909
#77	Add	Search **hospital[tiab] OR hospitals[tiab]**	665883
#76	Add	Search **institution[tiab] OR institutions[tiab] OR institutional[tiab]**	136739
#75	Add	Search **organisation[tiab] OR organisations[tiab] OR organisational[tiab] OR organization[tiab] OR organizations[tiab] OR organizational[tiab]**	253800
#73	Add	Search **organizations,Nonprofit[MeSH:noexp]**	2574
#72	Add	Search **organizations[MeSH:noexp]**	6080
#71	Add	Search **health facilities[MeSH:noexp]**	10797
#70	Add	Search **academic medical centers[MeSH Terms]**	65009
#69	Add	Search **ambulatory care facilities[MeSH Terms]**	40857
#68	Add	Search **birthing centers[MeSH Terms]**	486
#67	Add	Search **dental facilities[MeSH Terms]**	7564
#66	Add	Search **fitness centers[MeSH Terms]**	298
#65	Add	Search **hospital units[MeSH Terms]**	70715
#64	Add	Search **hospitals[MeSH Terms]**	187603
#63	Add	Search **nurseries[MeSH Terms]**	1943
#62	Add	Search **pharmacies[MeSH Terms]**	3571
#61	Add	Search **physician offices[MeSH Terms]**	1404
#60	Add	Search **rehabilitation centers[MeSH Terms]**	11199
#59	Add	Search **residential facilities[MeSH Terms]**	40661

#58	Add	Search (((((((((((((((((((((((((#32) OR #33) OR #34) OR #35) OR #36) OR #37) OR #38) OR #39) OR #40) OR #41) OR #42) OR #43) OR #44) OR #45) OR #46) OR #47) OR #48) OR #49) OR #50) OR #51) OR #52) OR #53) OR #54) OR #55) OR #56) OR #57	7346
#57	Add	Search **"recherche action"[tw]**	1
#56	Add	Search **"democratic evaluation"[tw]**	4
#55	Add	Search **"appreciative inquiry"[tw]**	85
#54	Add	Search **"cooperative inquiry"[tw]**	24
#53	Add	Search **"action science"[tw]**	14
#52	Add	Search **"community driven research"[tw]**	14
#51	Add	Search **"participatory evaluation"[tw]**	87
#50	Add	Search **"community based action research"[tw]**	14
#49	Add	Search **"community based participatory research"[tw]**	1732
#48	Add	Search **CBPR[tw]**	427
#47	Add	Search **(action[Title] AND research[Title])**	2167
#46	Add	Search **(participatory[Title] AND (research[Title] OR design[Title]))**	741
#45	Add	Search **"empowerment evaluation"[tw]**	20
#44	Add	Search **"social reconnaissance"[tw]**	2
#43	Add	Search **"emancipatory research"[tw]**	8
#42	Add	Search **"participatory appraisal"[tw]**	29
#41	Add	Search **"participatory rural appraisal"[tw]**	60
#40	Add	Search **"collaborative partnerships"[tw]**	159
#39	Add	Search **"collaborative partnership"[tw]**	185
#38	Add	Search **"collaborative action"[tw]**	116
#37	Add	Search **"collaborative inquiry"[tw]**	26
#36	Add	Search **"participative research"[tw]**	15
#35	Add	Search **"participatory research"[tw]**	2135
#34	Add	Search **"action research"[tw]**	2112
#33	Add	Search **(community institutional relations[MeSH Terms] AND (research[Title] OR research[MeSH Terms]))**	1120

#32	Add	Search **community based participatory research[MeSH Terms]**	1242

Grey literature search strategy

We attempted to develop a structured approach to searching grey literature that could be replicated in the future. We decided not to conduct hand searching.

Grey literature sources (database, organization, website)	Date searched	URL	Search terms	Number of all hits	Number of relevant documents out of all scanned	Notes / Observations
Google (advanced)	2013-03-14		allintitle: ("action research" OR "participatory research" OR " community based action research") (hospital OR "care facility" OR clinic OR "health trust" OR ward OR pharmacy OR nurses OR physician OR therapist) Notes: no language limits, ranked by relevance	531	Two raters scanned and reached consensus on 50 hits (9.4% of all hits, not blind rating). 11 documents included based on eligibility criteria.	Out of the scanned 50 hits, 23 were duplicates with documents already included in our sample from searching in bibliographic databases. The search was limited to title only in order to have a more manageable sample. Therefore, most of the retrieved hits were journal articles or theses.
The New York academy of Medicine, Grey Literature Report	2013-03-14	http://www.greylit.org/	"participatory research" Search field: full text, Search all reports,	8	Two raters scanned and reached consensus on all 8 hits (not blind). All 8 were excluded.	The retrieved hits dealt with community-based participatory research.

OpenGrey	2013-03-14	http://www.opengrey.eu/	("action research" OR "participatory research" OR " community based action research") AND (hospital OR "care facility" OR clinic OR "health trust" OR ward OR pharmacy OR nurses OR physician OR therapist)	4	Two rates scanned and reached consensus on all 4 hits. Two were included.	Judgement was made based on title and provided keywords, as abstract or full text were not available.
OpenGrey	2013-03-14	http://www.opengrey.eu/	"action research" discipline:(05T - Health services, health administration, community care services)	33	Two rates scanned and reached consensus on all 33 hits. 10 were included.	Same as above
ProQuest Dissertations & Theses: UK & Ireland: Health & Medicine, ProQuest Dissertations & Theses Full Text: Health & Medicine	2013-03-15	http://search.proquest.com/health	su.Exact("action research" OR "participatory research") Manuscript type: Doctoral dissertations, Master's theses Language: English, French	95		The core research group decided to limit the search for theses and dissertation to subject headings only (not searching with keywords).

Process for identification of grey literature: The two raters (PB and VG) scanned all hits (titles and abstracts when available) simultaneously / in real time, mostly since the content (such as grey literature found through Google is constantly changing). Through discussion (not blind process), they reached agreement. They used pre-established eligibility criteria for identification of potentially relevant hits. Since these two raters have been coding (independently) to identify (nearly 9000 research abstracts) and select (125 full text articles) potentially relevant studies using established eligibility criteria, they have a high degree of agreement and coherence. In scanning grey literature resources, they looked for evidence of organizational change in projects where health organizations were involved in participatory research.
