# Supplementary material for: A systematic mixed studies review on Organizational Participatory Research: towards operational guidance
Source: BMC Health Serv Res. 2018 Dec 22;18:992. doi: 10.1186/s12913-018-3775-5 (PMC6421946; doi:10.1186/s12913-018-3775-5)
Supplement: Supplementary file 3 — Eighty-three Organizational Participatory Research Summaries. This document includes the 83 OPR summaries organized according to four ideal-types of OPR. (PDF 873 kb) [file 12913_2018_3775_MOESM3_ESM.pdf]

Eighty-three Organizational Participatory Research Summaries Organized According to Four ideal-types

[*TYPE 1: BASIC OPR ....2*]

[*TYPE 2- RANDOM SPARKS.. 71*]

[*TYPE 3 : REPLICATION .... 93*]

[*TYPE 4: INITIATION ....108*]

[*ALL 4 TYPES OF OPR ....116*]

Type 1: Basic OPR

ANDREWS, S., MCINERNEY, F., & ROBINSON, A. (2009) 5

ARCIDIACONO, C., VELLEMAN, R., & PROCENTESE, F. (2010) 6

ATWAL, A., & CALDWELL, K. (2002) 7

BELLMAN, L., & CORRIGAN, P. (2010) 8

BLOMQVIST, K., THEANDER, E., MOWIDE, I., & LARSSON, V. (2010) 9

BREDA, K. L., ANDERSON, M. A., HANSEN, L., HAYES, D., PILLION, C., & LYON, P. (1997) 10

BRIDGES, J., MEYER, J., & SPILSBURY, K. (2000) 11

BRYANT, W., VACHER, G., BERESFORD, P., & MCKAY, E. (2010) 12

CASHMAN, S. B., FLANAGAN, P., SILVA, M. A., & CANDIB, L. M. (2012) 14

CHEN, J. C., GOETZ, M. B., FELD, J. E., TAYLOR, A., ANAYA, H., BURGESS, J.,ET AL. (2011) 15

COSTA, VALCARENGHI, R. V., DEVOS, TAROUCO, DE ALMEIDA, S., & EGUES. (2010) 16

CRAIG, D., SELLER, M., DONOGHUE, J., & MITTEN-LEWIS, S. (2004) 17

CRILLY, T., & PLANT, M. (2007) 18

DANIELS, L., & LINNANE, J. (2001) 19

DEGERHAMMAR, M., & WADE, B. (1991) 20

DEWING, J., & TRAYNOR, V. (2005) 21

DOBRANSKY-FASISKA, D., NOWALK, M. P., CRUZ, M., MCMURRAY, M. L., CASTILLO, E., BEGLEY, A. E., ET AL. BROWN, C. (2012) 23

EISENBERG, E. M., BAGLIA, J., & PYNES, J. E. (2006) 25

EVANS, P. H., GREAVES, C., WINDER, R., FEARN-SMITH, J., & CAMPBELL, J. L. (2007) ... 26

FINLEY, G., FORGERON, P., & ARNAOUT, M. (2008) 27

GERRISH, K., CLAYTON, J., NOLAN, M., PARKER, K., & MORGAN, L. (1999) 29

GREGORY, S., POLAND, F., SPALDING, N. J., SARGEN, K., MCCULLOCH, J., & VICARY, P. (2011) 30

HAMELIN BRABANT, L., LAVOIE-TREMBLAY, M., VIENS, C., & LEFRANCOIS, L. (2007) 31

HARRIS, L. E. (1998) 33

JONES-BAUCKE, D. L. (1997) 35

JOYNER, K., & MASH, B. (2012) 36

KHUNGERN, J., KRAIRIKSH, M., TASSANIYOM, N., & SRITANYARAT, W. (2006) 37

LAURI, S. (1981) 38

LAUSTEN, G. (2007) 39

LINDEMAN, M., SMITH, R., VRANTSIDIS, F., & GOUGH, J. (2002) 40

LUCAS, B., COX, C., PERRY, L., & BRIDGES, J. (2013) 42

MALUS, M., SHULHA, M., GRANIKOV, V., JOHNSON-LAFLEUR, J., D'SOUZA, V., KNOT, M., ET AL. (2011) 43

MASH, R., LEVITT, N. S., VAN VUUREN, U., & MARTEL, R. (2008) 44

MASON, R. A. (2003) 46

MCKELLAR, L. V., PINCOMBE, J. I., & HENDERSON, A. M. (2006) 47

MILLS, J., & FITZGERALD, M. (2008) 48

MIRZA, M., GOSSETT, A., CHAN, N. K., BURFORD, L., & HAMMEL, J. (2008) 50

MITCHELL, E. A., CONLON, A. M., ARMSTRONG, M., & RYAN, A. A. (2005) 51

MUNN-GIDDINGS, C., HART, C., & RAMON, S. (2005) 52

NGWERUME, K. T., & THEMESSL-HUBER, M. (2010) 53

OLSEN, L., & WAGNER, L. (2000) 54

ORFORD, J., TEMPLETON, L., COPELLO, A., VELLEMAN, R., IBANGA, A., & BINNIE, C. (2009) 55

OTTMANN, G., LARAGY, C., ALLEN, J., & FELDMAN, P. (2011) 56

SPALDING, N. J. (2004) 58

SENESAC, P. M. (2004) 60

SIEBENS, K., MILJOEN, H., GEEST, S. D., DREW, B., & VRINTS, C. (2012) 61

SMITH, S., DEWAR, B., PULLIN, S., & TOCHER, R. (2010) 62

SMITH, S. E. (1995) 63

TOLSON, D., IRENE, S., BOOTH, J., KELLY, T. B., & JAMES, L. (2006) 64

WALLIS, M., & TYSON, S. (2003) 66

WATERMAN, H., & GRABHAM, J. (1995) 67

WILLIAMS, A., SELWYN, P. A., MCCORKLE, R., MOLDE, S., LIBERTI, L., & KATZ, D. L. (2005) 69

Andrews, S., McInerney, F., & Robinson, A. (2009). Realizing a palliative approach in dementia care: Strategies to facilitate aged care staff engagement in evidence-based practice. International Psychogeriatrics, 21*(SUPPL. 1), S64-S68.*

With an academic researcher who acted as a collaborator/facilitator, staff members from a residential dementia special care unit (SCU) formed an action research group (ARG) and undertook an investigation into their practice to explore how they could develop strategies to support a palliative approach to care provision. The ARG met over a sustained period of time to discuss and reflect on their practice. Each meeting was recorded and transcribed. Transcripts were developed into case notes, which provided a first level analysis of the discussions. The notes were returned to the group prior to each successive meeting, in order to facilitate critical reflection on key emerging issues and provide a marker of progress in the ARG's efforts to facilitate practice change. The meetings provided ARG members with an opportunity to explore a range of ideas which they considered important to developing their practices to support a palliative approach to care.

The ARG engaged in a preliminary investigation where they shared information and understandings about their current practices. Findings validated the group's perceptions that family caregivers had limited knowledge about dementia, with little understanding of the biomedical basis of the condition or the relationship between dementia and deaths. Staff members reflected critically on the interview data and on their own passivity in accepting the status quo. Through their critical dialogues, the group agreed to take action to address family members' information needs and, in doing so, to engage proactively with evidence which would assist them to achieve this change in practice. They developed an information package which was distributed to family caregivers of all residents on the unit. This initiative was evaluated by the university partners and returning these findings to the ARG provoked members to share accounts of their observations, which included family members presenting to staff as "less anxious" and "less distressed" by resident behaviors, being more "accepting" of specialized care practices on the unit and being more confident to engage in discussion about their relatives' condition or care needs.

For members of the ARG, their experiences of successfully effecting change on the SCU cannot be overstated – the entire group shared in the knowledge that they had made a real, tangible difference to the care of family members. This was an important transformation from their initial position, where the group had been unable to envisage a situation other than the status quo. Indeed, in the months following the completion of the action, ARG members remained inspired by what they had achieved.

Arcidiacono, C., Velleman, R., & Procentese, F. (2010). A synergy between action-research and a mixed methods design for improving services and treatment for family members of heavy alcohol and drug users.* Journal of Community & Applied Social Psychology, 20*(2), 95-109. doi:https://doi.org/10.1002/casp.1015

Together with university researchers in Italy, physicians, nurses, psychologists, and other professionals working for the local Addiction Services sought to collaborate on the themes of coping for families, substance misuse, and treatment. This led to a productive inter-institutional synergy in which a number of organizations and institutions from Universities and the health and social care sectors in Northern, Central and Southern Italy came together. As a participatory research project, care was taken to listen to the voice of family members, use a new procedural approach to deal with their problems, get a large number of different professionals and organizations involved in a rigorous and wide-ranging debate, and propose a different model of service-providing as a final organizational task.

Peer-group expert discussion, argument and consensus development was a focal point of the research. For example, through regular research group meetings they clarified and standardized procedures and evaluated results of interventions. This meant investing time and resources that were not initially planned on arranging joint meetings, in order to validate and assess together the data gathered and discuss the difficulties encountered in implementing protocols. Nonetheless it was precisely this activity which fostered a systematic reflection by the researchers and practitioners, drawing on their different interpretations of the social situation and the quality and relevance of the results of the research. For instance, they achieved a comprehensive picture of the data gathered in light of the specific experiences and backgrounds of the researchers and practitioners, and their fruitful discussions of preliminary research reports sometimes led them to re-read the interview reports together, paying special attention the different viewpoints within the research team. Moreover, focusing on the contribution of the various professionals was helpful in exploring and elaborating the reciprocal prejudices and stereotypes among university staff and health and social care practitioners from many different backgrounds. Researchers were able to learn from the experiences of the organization members, and they in turn were able to learn from the researchers. The interaction of the multi/ inter-disciplinary partners is one of the main strengths of this project.

Atwal, A., & Caldwell, K. (2002). Do multidisciplinary integrated care pathways improve interprofessional collaboration?* Scandinavian Journal of Caring Sciences, 16*(4), 360-367.

Physicians, nurses, and occupational and physiotherapists sought to enhance interprofessional working by implementing an interprofessional discharge model in an acute orthopedic hospital ward in England. Due to the hierarchical and bureaucratic culture of English health and social services, it was necessary to create a steering group of managers who held the power to make decisions affecting the organization, and a distinct action research group of practitioners. The action research group debated, and accepted or rejected, many of the ideas and issues raised by the steering committee. The university partner documented the change process and provided the group and the committee with feedback, such that the change process could be understood, structured, modified, or accelerated. The study brought about change by involving practitioners in the change process, regardless of their status. This was achieved by investing time in communication strategies, such as providing space for professionals to voice their fears, experiences, and opinions regarding the change, circulating drafts of modified forms for feedback, and attending group and /or individual meetings to ascertain a wide range of opinions. Although the action research study did not improve interprofessional working, it did change clinical practice for the better in that it encouraged practitioners to think about their interprofessional practice and ways to improve collaboration and patient care.

Bellman, L., & Corrigan, P. (2010). Using action research to develop a thoracic support nurse role to enhance quality of care. Nursing times, 106(22), 18-21.

A research team of 8 nurses including the senior research and development nurse met monthly over a period of 9 months to develop a thoracic specialist nurse (TSN) role. Specifically, they set out to: explore the process of change with the TSN and colleagues; highlight the drivers and barriers to developing the role; explore problems in TSN role development; and evaluate the impact of the TSN role. The team co-wrote and submitted the internet research information system form for ethical approval; designed patient and staff consent forms and participant information forms; developed the research project timeline; acquired advanced literature search skills; developed a thoracic specialist nurse daily activity sheet; created an online survey tool; facilitated audiotaped focus groups; designed a staff questionnaire and patient survey tool; and presented the study at an international nursing research conference. Analyses revealed that the nurse co-researchers experienced a rollercoaster of emotions, felt that they had done the work themselves and learned a lot, and considered that process is as important as outcome. The nurse co-researchers tried and succeeded in making research come alive in everyday practice for patient benefit, and patients and staff recognised how the new role greatly improved thoracic nursing care. However, there were many personal and professional challenges to address including TSN role conflict, trust wide demands on the CNS, and reducing the support she could offer TSNs.

Blomqvist, K., Theander, E., Mowide, I., & Larsson, V. (2010). What happens when you involve patients as experts? a participatory action research project at a renal failure unit. Nursing Inquiry, 17(4), 317-323.

This PAR study aimed to explore what improvements patients with chronic renal failure asked for at a specialist clinic, and to use their expertise as a tool to develop patient-centred care. The project was carried out by a core group (2 nurses from the renal failure, a researcher working in a PAR unit at the nearby university, and the head of the development unit at the hospital) and an outer research group (4 patients with chronic renal failure). A focus group showed that the expert patients were satisfied with most of the care but revealed four areas that needed to be developed. The core group met to analyse, reflect on, and plan changes based on focus group meetings together with the expert patients. The meetings in the core group were open, and knowledge, ideas and opinions were exchanged freely.

After almost 2 years of planning and working together within the core and outer groups, and by adapting the project to a continuously changing everyday reality, experience indicates that had the project been designed as a traditional research project (i.e., planned in detail from the very beginning) sustainable change in practice would not have eventuated. The study indicated that although none of the 4 development areas were totally new to the members in the core group, their understanding differed somewhat from that of the expert patients. With respect to the timing of the receipt of information about the progress of the illness, for example, the expert patients helped the core group members see and differentiate information that should be provided in the early and late phases of the condition, respectively; and, members of the core group noticed a shift in their own way of thinking about patients, and in the actions of the expert patients. Also, running the project on a long-term and low intensity basis, and involving people who were deeply committed, felt responsible, and had a mandate to make changes were deemed necessary for the project.

Breda, K. L., Anderson, M. A., Hansen, L., Hayes, D., Pillion, C., & Lyon, P. (1997). Enhanced nursing autonomy through participatory action research.* Nursing outlook, 45*(2), 76-81. doi:10.1016/S0029-6554(97)90084-1

Following a conscious-raising retreat, in a small, private psychiatric hospital in rural New England a group of 12 nurses representing both staff and supervisory levels from all in-house client care units was formed to share ideas about clinical practice, and ultimately, collaboratively explore ways to increase autonomy of nurses in the workplace. Due to fluctuation in weekly meeting attendance, a core group of 6 nurses completed the study, with the other 6 nurses keeping up-to date with the project. The group was a vehicle that allowed hospital nurses to learn from each other, to share experiences, and to produce knowledge that was useful to them. The open group study format allowed consciousness-raising among members and resulted in an increased awareness of their position as nurses in the hospital. Within 2 months, the group had uncovered and remedied gaps in their knowledge base, had made substantial progress in discovering areas of strength and expertise, and begun exploring new avenues of care. They also began to question some of their practices previously taken for granted, and became increasingly assertive with physicians, therapists, and administrators, considering themselves active, rather than passive, agents in client care. They learned to value their own knowledge and experiences and came to see that part of the responsibility of psychiatric mental health nurses is to act as a liberating role model and mentors for clients and staff.

Positive changes in the hospital milieu as a result of the new interventions by the study group's nurses are significant and they remain active in the hospital as central figures in the education and consciousness-raising of clients and staff. Overall, the study group nurses' development of a critical consciousness and refusal to be treated and to treat others as objects of oppression produced significant movement toward nursing autonomy. Their active participation in the same system that had previously limited their growth and self-determination allowed them not only to question oppressive practices but also to begin to transform them. The group realised that having confidence and pride in their knowledge and skills was an important precursor to both autonomy and empowerment.

Bridges, J., Meyer, J., & Spilsbury, K. (2000). Organisation of care for older people in A&E. Emergency Nurse, 8*(3), 22-26. doi:10.7748/en2000.06.8.3.22.c1323*

In 1996, service managers at Barts and The London NHS Trust (a large inner London Trust in England that provides acute in-patient hospital care at the Royal London Hospital for adult non-surgical patients) had introduced a new role to the workforce, that of interprofessional care co-ordinator (IPCC). This study sought to explore the impact of the development of the new role of IPCC on interprofessional team working in the acute medical in-patient setting. The development work within the study took place within three main action research cycles: 'Communicating about the IPCC role', 'Exploring issues of accountability' and 'Improving interprofessional working'. In each cycle, the lead investigator worked alongside hospital staff to negotiate appropriate research methods informed by practice and emerging findings, and to use emerging study findings to prompt new reflections and initiate practice changes. She adopted a facilitative style, using her interpersonal skills to enable others to share their experiences, views and ideas. Staff used study findings to reflect on the IPCC role and the context in which it operated and to devise plans of action to make improvements in practice. An example of this is where study findings reflected that interprofessional communication was significantly impaired as a result of the way the doctors organized their input to the wards. As a result, the clinical director took the lead in an initiative that resulted in medical staff across the service changing their input to ward-based medical teams. During the final phases of data collection, service managers were asked to reflect on the reasons why some planned change had not taken place. They identified that the pressing nature of other organizational priorities meant that there was no capacity for making certain changes.

It is possible that the changes may have happened anyway in the absence of the action research study, although feedback from participants suggested that this would not have been the case. Moreover, the early involvement of senior personnel such as the service's clinical director and operations managers was crucial.

Bryant, W., Vacher, G., Beresford, P., & McKay, E. (2010). The modernisation of mental health day services: Participatory action research exploring social networking. Mental Health Review Journal, 15*(3)*, *11-21*.

This research, designed to explore service user perspectives on social networking in mental health day services. An initial focus group study generated recommendations for service development and indicated that service users and staff wished to be involved in the process. A forum was set up in 2003 to take forward the recommendations, incorporating the modernisation agenda in 2004. Open meetings were held for two hours every six weeks in the social lounge of one of the three local resource centres. At least ten (and usually more than twenty) service users and staff attended every meeting. Interpretations of the modernisation policy were discussed, leading to the development of the research questions, aims and design. Forum members then became involved in promoting the research and recruiting others. In 2007, a forum meeting was held to reflect on the research and on shaping the final analysis. Translating opinions and ideas into action required a more intense focus, so two action days, focused on social networking, were held to reach a wider audience. Both were planned by working groups from the forum, including service users. Locating the discussions within the research project meant that staff were not forced to defend the modernisation and could learn service users' fears about day services.

The first research strand investigated how service users used a social lounge in one of the resource centres for social networking. Five service users, the first author and a staff member were involved in the research group, which met weekly for eight sessions to conduct a photovoice study. A working group of service users resulted from this strand to advise on the design of a safe, social space at the resource centre. The second strand was refined from an initiative by service users at the first social networks action day. They collated details of places, recommended during the action day, into a directory, which was distributed to all local day services. The third strand emerged directly from the forum's interest in the user-led self-help groups, which were being promoted as an alternative way of social networking. One approach to dissemination was a poster summarising the findings, copies of which were displayed in the local resource centres and inpatient units. The posters provided a resource for service users and staff to discuss social networking and social inclusion. This approach to dissemination also indicated to the forum and local services that collaborative research could rapidly generate useful findings.

The ongoing forum, two action days and three strands enabled new understandings to emerge and develop, and the varied approaches to engaging service users enabled different voices to be heard. Service users in this research were surprised to discover their expertise on day services. While It was challenging for staff, who had multiple and complex roles, to shift focus from individual journeys to service design, evaluation and development, their involvement was critical to ensuring the continued relevance, feasibility and accessibility of the research and its outcomes. The inclusive approach meant that it was possible to acknowledge their critical perspectives. Precedence was given to service user perspectives, but the inclusion of staff secured access for many who would otherwise have struggled to be involved. Equally, the value placed by staff on the research outcomes meant that the findings were rapidly accessible to a wider local audience. Using different approaches engaged people successfully. Ownership was promoted by placing value on people's actions.

Cashman, S. B., Flanagan, P., Silva, M. A., & Candib, L. M. (2012). Partnering for health: collaborative leadership between a community health center and the YWCA central Massachusetts*. Journal of public health management and practice : JPHMP, 18*(3), 279-287. doi:10.1097/PHH.0b013e3182294fe7

Given their shared broad health improvement and social justice goals, YWCA health promotion staff, medical school faculty, and a physician and pharmacist from a Community Health Center (CHC), formed a partnership to allow low-income CHC patients to use the YWCA for physical activity at no charge. The YWCA viewed the partnership as successful and CHC partners were particularly pleased that the partnership made it possible for them to encourage patients to access a physical activity facility, rather than to simply advise them to engage in physical activity. Challenges were encountered (e.g., integrating a large number of inexperienced users into the YWCA's usual operation) and partners felt more in-depth planning and detailed and explicit principles for their partnership could have helped. Ultimately, partners felt their regular meetings (once every 1-2 months) were crucial to their success; indeed, the core group developed a cohesive identity. Also important were partners' commitment, strong leadership, and effective communication (e.g., care and attention paid to partners' needs and ensuring all parties understood the reasons for decisions). Notably, the partnership developed from a strong, multiyear, and ongoing connection between the health center's physician leader and YWCA administrators and staff; existing relationships may have facilitated the development of this particular partnership.

Chen, J. C., Goetz, M. B., Feld, J. E., Taylor, A., Anaya, H., Burgess, J.,et al. (2011). A provider participatory implementation model for HIV testing in an ED. **American Journal of Emergency Medicine, 29*(4), 418-426*.** *DOI:***10.1016/j.ajem.2009.11.016**

Members of the Division of Infectious Diseases, researchers in the VHA HIV/Hepatitis Quality Enhancement Research Initiative center, and clinical champions and administrators from the full-time ED attending physicians and nurses met regularly before and during the implementation of ED-based HIV screening. The testing protocol was modified based on nursing feedback about the heavy burden on triage staff and lack of control over fitting HIV screening into workflow. Data collection methods evolved iteratively out of the collaborative research process, as the initially conceived data collection sheet methods were felt to compose an overly burdensome research apparatus as perceived by the ED staff community.

The impact of using a CBPR approach to development and implementation of ED-based HIV screening is mixed. As no HIV-positive patients were identified, there exists a question of whether the CBPR approach somehow produced a bias that selected for a very low risk population. A CBPR approach may also have led to the unintended outcome of handicapping the potential of this ED-based HIV screening implementation by giving too much power to the ED staff to limit the overall numbers tested per day. Although there was no clear majority among our ED staff in terms of preferred HIV testing models, the plurality preferred the current spare capacity model generated out of the CBPR process

Costa, Valcarenghi, R. V., Devos, Tarouco, de Almeida, S., & Egues. (2010). Development of a medical record for residents in a long-stay institution for the elderly. Acta Paulista de Enfermagem, 23(6), 725-731. doi:10.1590/S0103-21002010000600002

Following a visit to the Home for the Aged in Rio Grande do Sul, Brazil, the Conselho Regional de Enfermagem – REN/RS (RegionalNursing Council) asked the GEP-GERON group (professors and students of the nursing college undergraduate and graduate programs), to contribute to improving the care the institution provided to the elderly. The need for records to be implemented emerged. A meeting with the administrators of the Home was held and a commitment to elaborate a record model was made, as long as the whole institution team helped the group in this challenge. A work group was formed and developed the research objective: to elaborate residents' records for the residents of the Home.

In five monthly meetings reflections/discussions on the research objectives and specific themes took place. Between meetings, records materials were shared via email. A record model of improved assessment scales, adapted to the patients and professionals resulted. This record model was tested on one patient and in the last meeting, the results were presented, as well as pertinent modifications made to it. During this final meeting, the work group also assessed its process and celebrated, demonstrating satisfaction after the collective work had been concluded. Fifteen days after the work group fifth meeting, the investigated Home for the Aged resident record model was concluded.

Positive aspects of the study included work group commitment; university partners' organising the meetings and providing printed copies of the material to discuss; access to computers to facilitate the work; and provision of coffee and water during meetings. Negative aspects included participation (among the 16 participants, only 10 attended all meetings; one of the participants was only present in two meetings) and the fact that work group members' contributions had to be truncated in the final report, not doing justice to the richness of their reflections/ discussions/testimonies. Meetings and collective reflections/discussions were interesting and helped the involved individuals better know each other and reach the expected results: the construction of a product, in this case, a record model elaboration and implementation at a specific Home for the Aged.

Craig, D., Seller, M., Donoghue, J., & Mitten-Lewis, S. (2004). Improving nurse management of patients with diabetes using an action research approach.* Contemporary Nurse: A Journal for the Australian Nursing Profession, 17*(1-2), 71-79. doi:10.5172/conu.17.1-2.71

This study sought to motivate nurses to upgrade their standard of care of dependent patients with diabetes (Type 1 and type 2) on the Acute Care Nursing Professional Unit in an Australian hospital. The research group, composed of about 25 staff from 2 wards, met regularly on over 20 occasions throughout the research period. The clinical nurse consultant (CNC) and diabetes educator (DE) undertook two observational audits on blood glucose level (BGL) monitoring practice activities at 16-month intervals. The results of the first audit were tabled in a research group meeting and a decision was made to develop a new blood glucose chart that allowed the actual times of BGL measurement and patients' meals to be recorded accurately. Guidelines were provided to facilitate the use of the new form. The CNC and DE sought the opinions of nurses working on the wards who decided not to join the research group and discussed the use of the new chart with them. After 6 months, the research group decided to implement a 'ward communication book'. Importance of accurate documentation was discussed at practice meetings and the CNC and DE assisted junior nurses to develop confidence in changing their practice.

The second audit served to inform the research group of any changes in practice. Eighty percent of the 42 ward nursing staff changed their practice and endorsed the process as helping develop practice. The use of action research process enabled collaboration among ward nurses, the CNC, the DE and staff from the unit to change care for patients' benefit. Involvement in the development and ongoing use of a revised blood glucose monitoring chart (now introduced throughout the hospital) resulted in a behavioural change that improved the coordination of BGL assessment and patients' meals.

The eighteen-month time frame of the project was longer than anticipated, but perhaps due to nurses rotating shifts, and a mix of part time and casual staff. It was impossible for nurses on the 2 wards to meet at once, thus, multiple meetings were held to discuss the same issues and develop consensus. Also, some nurses openly resisted involvement in anything identified as 'research'. It also took some time to allay fears about modifying a chart that had existed unchanged for years (some felt their standard of work was being challenged). The ward communication book was helpful for involving those who could not attend meetings (e.g., those on night shifts), and progress updates were provided to patients and medical and allied health professional staff. A report was also published in the annual nursing monograph for the hospital. Having undertaken this project, the next practice change on these wards will be easier because a new attitude to practice formulated from evidence has evolved.

Crilly, T., & Plant, M. (2007). Reforming emergency care: Primary care trust power in action research. Health Services Management Research, 20(1), 37-47. doi:10.1258/095148407779614954

A Primary Care Trust used its position as lead commissioner in a health economy to search for efficiency gains and to improve the patient journey through accident and emergency (A&E) services in a hard-pressed acute hospital. Four project groups were formed to undertake the study. Each group included representation from primary care general practitioners, hospital consultants, hospital and community nurses and hospital managers, as well as commissioning agents from Social Services and the two main Primary Care Trusts. The project team also included a researcher who was active across all groups to steer the process of collaborative enquiry and develop data specifications and analytical frameworks. They developed three lines of enquiry: (i) the patient journey; (ii) measures of performance; and (iii) qualitative factors.

Although A&E was the primary focus, it became apparent during the fieldwork that, to address the views of staff delivering the service in A&E, it was necessary to widen the line of enquiry into the nature of capacity within the whole local health economy. To state it more plainly, the overwhelming feeling among staff was that their experience of working in a beleaguered department was explained entirely by bed capacity problems, which lay with the rest of the hospital. In order to engage with staff and bend the culture to one that was reflective and capable of embracing change, it was necessary to follow through and investigate their hypothesis.

The methodology sustained a high level of energy and engagement throughout the short intensive period of collaborative enquiry. It generated a rich set of qualitative and quantitative information that was illuminating and afforded the potential for extensive redesign of processes. Two years later, the study hospital is able to claim success in achieving its main target of reducing delays in the system, measured through elimination of the 4 h wait in A&E and elimination of overnight sleepers.

Daniels, L., & Linnane, J. (2001). Developing a framework for primary palliative care services. British journal of community nursing, 6(11), 592-600. doi:10.12968/bjcn.2001.6.11.9451

A collaborative study was undertaken between an academic unit and primary care practice in England to evaluate current care provision and explore methods of developing services. The first phase of the study can be considered as a period of team building and value clarification by primary care team members (GP, nurses, health visitor, social worker, and practice counsellor). This was facilitated by the researcher during a series of 9 meetings which involved practitioners reflecting critically on their role and the delivery of palliative care services, clarification of personal, professional and team values regarding palliative care delivery, and the assessment of practice problems and the identification of goals and solutions.

A number of practice initiatives were developed by the primary care team and the researcher to change current palliative care service delivery and were implemented in practice over a period of 6 months. These initiatives were formally evaluated using the views of patients/carer and professionals and audit data. Results from questionnaires and interviews with professionals were used to develop a staff handbook outlining individual roles along with referral and contact information. Evaluation of changes indicated improved multiprofessional contact with patients, fewer patients reporting unmet needs.

The regular team meetings promoted open dialogue, and enhanced multidisciplinary communication and team working and understanding of each other's' roles. Team working was considered to reduce duplication of visits to patients and repetition of assessment procedures and so reduce inappropriate referrals. The use of local specialist services was also considered by the team to promote collaborative relationships. The study enabled a framework for multidisciplinary palliative care practice to be devised and facilitated the continuation of a multidisciplinary approach to the delivery of primary care services which is currently being integrated into the development of primary care group working. It also enabled practitioners to develop research skills and fully participate in data collection techniques as a means of assessing practice problems, value clarification and goal setting. A major strength of action research as an approach to this project is that practice evolved in response to changing needs and within the control of the practitioners.

Degerhammar, M., & Wade, B. (1991). The introduction of a new system of care delivery into a surgical ward in Sweden.* International Journal of Nursing Studies, 28*(4)*, 325-336. *doi:https://doi.org/10.1016/0020-7489(91)90059-C

In 1986 the Care Research Unit, Lund and Kristianstad, received a request from the head nurse of a 30-bed surgical ward. Help was needed to plan and implement a change in the organization of the ward. It was claimed that the current task-centred mode of organization had led to low morale among registered nurses who felt that they were unable to give adequate care. The Research Unit acceded to the request, a committee (comprising the head of the hospital, the director of nursing services, a trade union representative, and a researcher) was formed to: improve the quality of nursing care; introduce a patient-centred mode of care delivery; and monitor the effects of change on job satisfaction and on staff behaviour. Over several months, members of the group studied the literature on primary nursing and made visits to other hospitals in which systems of individualized care had already been introduced. In March of the following year interviews were held with the entire nursing staff on the target ward and a baseline observation study was carried out. The findings were fed back to the committee. In January 1988, the committee agreed to the introduction of a modified form of primary nursing known in Sweden as "pair care".

A staff training programme consisting of lectures and group discussions on topics such as ethics, communication skills, interview techniques and care planning began during the same month and continued throughout the development programme. The researcher held regular meetings with staff to discuss any problems that arose. Initially, meetings focussed on practical matters (e.g., a system of colour coding to aid identification and the allocation of responsibility for domestic matters). Once the initial practical problems related to the change in organization were overcome, the nurses became more creative, seeking ways in which they could further improve the care that they gave. Nurses became concerned with reducing time spent on routines and with more complex issues arising from their increased contact with patients (e.g., they became more concerned with their patients' environment and with their own need for support when dealing with terminally ill patients and their relatives). Meetings with small groups were organized to provide support and counselling for staff, and after the head nurse and researcher attended a course to study the role of 'Head nurse', they gradually changed the role to one of supporter/educator and facilitator for staff with whom the head nurse discussed educational needs, job satisfaction and special interests.

By December 1989 the new system appeared to be self-sustaining, the project was terminated, and the researcher withdrew. Registered nurses went from spending less than a quarter of their time with patients to spending 60% of their time giving direct patient care. The complexity of the issues addressed by the change agent/researcher, the head nurse and staff involved in this study increased with the duration of the project.

Dewing, J., & Traynor, V. (2005). Admiral nursing competency project: Practice development and action research.* Journal of Clinical Nursing, 14*(6), 695-703. doi: 10.1111/j.1365-2702.2005.01158.x

The aims of the project were to work collaboratively with the practitioners to review critically their current ways of working and develop a competency framework that reflects the needs of the Admiral Nurse Service. The project activities were monitored and reviewed by the project commissioners and an advisory group made up of a range of stakeholders. The five phases of the action research project were agreed in consultation with the nurses and the Advisory Group.

Admiral Nurses agreed on a list of eight core competencies that adequately represented the work and the researchers drafted, with input from the nurses, the competency framework and the accompanying guidance documentation. The framework was piloted and findings were incorporated into a final version. All data were shared with the nurses first as primary stakeholders. Agreement with them as to whom else and how data were shared was agreed throughout the project. We used small prearranged meetings with the Admiral Nurses to share and promote a wider understanding of the analysis process and the findings. During these meetings we first enabled the nurses to describe their involvement, feelings and learning and then shared a summary of the analysis with the Admiral Nurses and provided an opportunity to discuss how the themes were developed. Each meeting was also used by the Admiral Nurses as a forum with which to discuss, with one another and the project researchers, whether they were satisfied with their current ways of working. In the last phase of the project, the researchers facilitated the project commissioners to develop plans that would ensure implementation of the competency framework. Through meetings, they provided opportunities for a range of stakeholders to share an overview of the final version of the competency framework, collect concerns and issues for how the framework could be integrated into practice, and discuss unresolved issues from the pilot. Importantly, findings from this phase of the project have been taken forward by the Consultant Admiral Nurse and an implementation group.

Over the duration of the project, the sense of research being done to them rather than with them remained a significant issue for some Admiral Nurses. Involvement of the nurses enabled the researchers to critically reflect on their own skills. At times some Admiral Nurses were unable to deal effectively with their discomfort. They criticized the approach the project researchers adopted to discuss the findings with the Admiral Nurses. What was represented within the themes was not what some of the Admiral Nurses expected to be generated. Thus, there was a mismatch between the Admiral Nurses as a whole and what the project researchers reported finding in practice. To work through such challenging phases on the project, researchers needed to providing a safe supportive climate and make use of their practice development skills (specifically, facilitation and transformational leadership). The feelings of discomfort then need to be worked through and learning established.

The Admiral Nurses' commitment to stay with the project, organizational commitment to embed the framework in their practice development strategy and support from service managers contributed to the success of the development and implementation of the Admiral Nurses' Competency Framework. The project commissioners are continuing their support for the framework by explicitly linking it with

work on standards of care, new job descriptions and, importantly, through the role of the Consultant Admiral Nurse.

Dobransky-Fasiska, D., Nowalk, M. P., Cruz, M., McMurray, M. L., Castillo, E., Begley, A. E., et al. Brown, C. (2012). A community-academic partnership develops a more responsive model to providing depression care to disadvantaged adults in the US. The International journal of social psychiatry, 58(3), 295-305. doi:10.1177/0020764010396406

Researchers and leaders of community organizations that serve the poor formed the Research Network Development Core (RNDC) to create new research projects to pursue mutual concerns stemming from mental health disparities. Initially, some of the community partners (CP) entered this partnership with reluctance due to past experiences of unmet commitments from academic institutions (e.g. promises not met, commitments for funding not kept). Trust had to be gained at the organizational leadership level before access to the agencies' clients was granted. Patience and perseverance were required. The partnership evolved through ongoing discussions and iterative review of ideas that eventually led to consensus at monthly meetings. The initial efforts involved a series of meetings with each potential partner to determine their interest in working with us on mental health issues and more importantly, for the researchers to gain a greater understanding about the context in which they work with older adults who might be suffering from depression. Iterative work took place during monthly meetings that were instrumental in promoting trust between the CPs and the RNDC, strengthening members' commitment to the collaboration, and strengthening the partnership through members' regular attendance. The partnership decided upon the major topics to be assessed, developed data collection tools, and determined actions based on results. The needs assessment introduced the group to a systematic method of collecting information about each organization, and its process and results were early benefits. Agencies used their individual reports to inform board members and in strategic planning, annual reporting, and funding proposals. Internally, leaders became aware of staff issues, concerns, and needs to help direct the organization's growth.

Differences of opinion were discussed openly, and decisions were made through consensus. Initially, the academic and community partners came to the table with very different ideas and expectations of the purpose of the partnership; finding common ground was challenging, but the diversity within the partnership provided an opportunity to develop a unique synergy to better serve economically disadvantaged adults. One of the most difficult challenges for CPs was the distinguishable gap between research and practice ("We do not know academia, and academics do not know the day-to-day practice" "We speak different languages"). The languages issue was resolved over time by interacting.

The value of commending the partnership and acknowledging the productivity of the collaboration cannot be underestimated as a means reinvigorating the relationship and sustaining the collaboration. An essential part of developing and maintaining enduring relationships with our CPs was to clarify goals and parameters that would facilitate the development of a constructive working framework. The mechanisms of discussing issues were perceived by CPs as opportunities to pursue issues to everyone's satisfaction. CPs also felt that the academics were curious about them which helped them to develop trust in the academics. The participatory research approach has kept partners engaged and has sustained their interest in additional research. The diverse blend of experts from the community and the academic investigators has created projects and programs that are better suited to and accepted

by the community and with greater chance for sustainability than could have been produced by either group independently.

Eisenberg, E. M., Baglia, J., & Pynes, J. E. (2006). Transforming emergency medicine through narrative: qualitative action research at a community hospital. Health communication, 19(3), 197-208. doi: 10.1207/s15327027hc1903_2

The goals of the study were to gain a better understanding of the challenges facing emergency departments in a local context and to use these insights to help this particular department develop more effective responses. The research team of academics and ER managers attended two department meetings, described the proposed study, and answered employee questions. They conducted structured observations and produced detailed field notes. Once the 6 months of observations were complete, the research team met to review field notes and identify cultural themes and produced a six-page single-spaced narrative reflective of the ER culture at CHC. Next, the ER director assembled an ad hoc group representing the various levels of status and positions in the ER to consider the narrative. During a 2 hr meeting, the narrative was read aloud and discussed. Employees suggested multiple revisions to the text. Two weeks later, a revised narrative was presented to the hospital's executive team in a similar fashion, emphasizing common themes and ensuring employee confidentiality and prompting further dialogue about its implications for action. A summary cultural narrative was presented to both ER management and senior hospital administration. Our observations were used by a newly formed interdisciplinary organizational development team seeking to create baseline measures and begin a number of targeted interventions. Some of the changes that occurred were a direct result of this study, and others reflected both our findings and ongoing efforts on the part of the director, the physicians group, and the nurse educator. The most significant changes in practice were: Hiring of an additional patient advocate and greeters to direct patients upon arrival to the ER; More timely physician communication with patients brought back to the treatment area; New approach to room stocking in the morning; Patient meal tickets made available during long delays; Improved triage through continuing education; Meetings with pharmacy and phlebotomy departments to clarify expectations and improve support.

In all, we were pleased with how our composite findings were used to transform the nature of the ER at CHC. Moreover, it appeared to us that this narrative provided new insights to ER employees that were useful in addressing persistent organizational problems. Many, if not most, of the dynamics identified in this study would not likely be visible from an outsider's perspective, using traditional social science methods. Given our decision to collaborate with insiders on our research focus and to observe the work directly over time from the perspective of an informed outsider not associated with a particular professional perspective resulted in a narrative that yielded fresh insights that were richly detailed and a better reflection of the emotional lived experience of both patients and employees. Working through the narratives with the ER staff gave them a role as research partners and enabled a dialogue that encouraged the ER staff to acknowledge and understand each other's ways of viewing the world and to include this awareness in their co-authorship of a new narrative.

Evans, P. H., Greaves, C., Winder, R., Fearn-Smith, J., & Campbell, J. L. (2007). Development of an educational 'toolkit' for health professionals and their patients with prediabetes: The WAKEUP study (Ways of Addressing Knowledge Education and Understanding in Pre-diabetes). Diabetic Medicine, 24(7), 770-777. doi: 10.1111/j.1464-5491.2007.02130.x

The WAKEUP study sought to identify key messages and to develop an educational toolkit to address the information needs of primary care health professionals and their patients with regard to the management of pre-diabetes. Health professionals and our service-user reference group were involved in a 'colearning' role (joint decision making with the research team). Discussions with an expert reference group and within the research team informed the development of topic guides for the focus group interviews and also generated ideas about key messages for patients with pre-diabetes.

In the feedback on the first draft of the toolkit a number of patients felt that the risk/fear messages needed to be conveyed more strongly. In response to this and focus group data from the Needs Assessment phase, a clear explanation of the risks was given alongside 'Good News' messages, such as the advantages of being 'caught early' and being able to do something about it. The inclusion of these positive messages was appreciated.

After the toolkit had been used with three patients in each practice, the data were analysed and the research team generated a summary, circulated it, and then presented to the health professionals and service users in action workshops, where ideas for change were reviewed and discussed.

Towards the end of the study, the health professionals from both practices reported being much clearer about the nature of prediabetes and the associated risks and placed more importance on acting systematically as a team to address the problem. They also reported that they had evolved clearer strategies for managing prediabetes within the pragmatic constraints of their everyday workload. The toolkit was found to be acceptable to both patients and practitioners and, when used in consultations, the key messages seemed to transfer well to patients. The practices involved in the study also made appropriate changes to their systems for managing prediabetes. The high level of engagement of patients and service users in the development of the WAKEUP toolkit was felt to be instrumental in improving its relevance and acceptability. The engagement of practitioners was similarly crucial.

Finley, G., Forgeron, P., & Arnaout, M. (2008). Action research: Developing a pediatric cancer pain program in Jordan. Journal of Pain and Symptom Management, 35(4), 447-454. doi: 10.1016/j.jpainsymman.2007.05.006

This capacity building program was aimed at developing, implementing, and evaluating a pediatric pain management program at KHCC, a dedicated cancer treatment facility that provides care unrestricted by the patient's ability to pay in Jordan. Canadian investigators undertook three 10-day visits to Jordan. During the first visit, the research plan was discussed at the initial meeting with the key participant group (4 physicians, 2 nurses, & 1 pharmacist), including the action research methodology and the planned interviews and focus groups. Key participants voiced their perceptions of organizational barriers and of staff and parent attitudes. Two physicians and a nurse clinician from the key participant group were identified as the appropriate staff members to form a pediatric pain management team and to act as champions. The other members of the key participant group would work with the team, providing support and advocacy for the necessary changes. The key participants reviewed the preliminary prevalence and interview data and had used these to develop policies for assessment, treatment, and documentation specifically to fit the available resources. Policy approval and establishment of an outpatient pain clinic were to take place before the next visit from the Canadian team members. Further staff education and formal pain management training of the local expert physician were planned. As suggested by the key participants, a positive endorsement from the hospital's Director General (the senior administrative officer) played a crucial role in ensuring institutional support. The visiting researchers met with senior administrators and department heads during each visit to keep them apprised of progress and include them in the process. Details of service structure and implementation were left to the key participant group and investigators.

During the second visit, the team refined and implemented the previously developed policies, based on staff and key participant feedback. The implementation schedule was changed and formal education plans for staff nurses were delayed due to the need to train a new pediatric nurse clinician. However, teaching sessions for attending physicians and residents took place, family education materials were created, and a pediatric pain management curriculum for health professionals was begun.

The primary focus during the final site visit was to plan continued capacity building among all staff and within the pediatric pain management team. Funds were secured to ensure that the pain management physician and nurse clinician would be able to attend an international pain conference. The need for an additional pain management physician was identified and recruitment activities are underway.

The broad impact of the program has been confirmed by trainees from other Middle Eastern countries, who stated that they would now have the knowledge and skills to help children in pain when they returned to their home hospitals. In this research program, the data informed the process of change. The staff focus groups and interviews illustrated the knowledge deficits and strengths of the staff members and also provided an opportunity to discuss clinician experience and to identify the preferred sequence of events (policy, mentorship, teaching). In addition, the information obtained allowed the key participant group to frame the information and actions in a manner consistent with

the culture of the organization. As of December 2006, the Pediatric Pain Service is actively caring for patients at KHCC.

Gerrish, K., Clayton, J., Nolan, M., Parker, K., & Morgan, L. (1999). Promoting evidence-based practice: managing change in the assessment of pressure damage risk. **Journal of Nursing Management, *7(6), 355-362. doi:10.1046/j.1365-2834.1999.00135.x***

The overall aim of the study was to facilitate the development of evidence-based practice, in the area of pressure damage, within a large acute teaching hospital. While the research team (3 nurse researchers) had identified the nature of the problem, addressing it required that the nurse managers (research partners) were committed to taking the work forward within individual directorates. The first step was for the research team to present the results of the study to the nurse managers from each directorate in order for them to decide on ways to disseminate the findings more widely and discuss their implications for nursing across the organization. Following meetings between the researcher and Directorate staff, a decision was made to form evidence-based practice groups for each of the three clinical areas, comprising nurse managers, audit facilitators, the researcher, the skin care specialist nurse and practitioners. Based on their observations of aspects that needed to be addressed, the groups worked towards more integrated intra- and postoperative care for patients and carried out focused literature reviews specific to their area of practice which could then inform protocol development. The audit of nursing records undertaken was limited in that it had included only a small number of patient records as well as an audit of assessment of pressure damage practice to provide a more accurate picture of current practice. Nurses from the clinical area, the skin care specialist and audit facilitators were involved in data collection using criteria derived from their literature search. The results of the audits were used alongside the literature to develop evidence-based protocols for the assessment of risk and prevention of pressure damage. Each directorate began to record risk and collect prevalence and incidence data to meet its own needs, to reflect resources available for the task, and forward the results to the skin care specialist nurse for trust-wide collation.

By focussing on a topic of relevance and feeding back specific findings to each directorate it was hoped that the managers would be committed to addressing the implications arising from the study with their directorates. However, not all directorates responded enthusiastically and where they have demonstrated the least interest little progress has been made. Moreover, as the study progressed, the research team became increasingly aware of the implications arising from the essentially top down approach taken. The problem assessment of pressure damage risk had been identified as an issue of concern by managers and researchers, not the nurses. This highlighted the need to engender ownership among nurses of the implications of the findings for patient care. The team sought to achieve this by communicating the findings in user-friendly format to the different stakeholders and providing the opportunity to discuss the implications of the study with groups of nurses who were keen to address the issues in their clinical areas. This enabled interested *nurses to buy in to the study and to address the issues raised by the study.*

Gregory, S., Poland, F., Spalding, N. J., Sargen, K., McCulloch, J., & Vicary, P. (2011). Multidimensional collaboration: Reflections on action research in a clinical context. **Educational Action Research, 19*(3), 363-378*. *doi:* *https://doi.org/10.1080/09650792.2011.600627***

This study, named PREPARE (Preoperative Education for Colorectal Surgery Patients and their Relatives) aimed to continuously evaluate and then make improvements to the preoperative education that is provided for nearly 500 patients who have colorectal surgery within the Trust each year. The team meets monthly and has worked together closely since the first day; planning the research, bidding for funding and actively involved in ongoing decision- making.

During the 'planning' stage of each action research cycle, the research team invites all of the health professionals working within the colorectal unit at the study site to attend a lunchtime meeting to discuss the potential changes to the preoperative education that the participants have identified and collaboratively decide which ones should be implemented. The meeting also provides an opportunity to highlight good practice and provide positive feedback from the participants. The sharing of this first decision-making process between the researchers and the healthcare professionals who provide the preoperative education presented several challenges. It was a stage when ownership of the action needed to pass from the research team to those actively involved in a practice that would be changed as a result of the evaluations

Using the suggestions for change that had been made by patients, carers and healthcare professionals, eight changes to practice were identified and it was agreed who would be responsible for their implementation. The changes are now being re-evaluated providing the opportunity for healthcare professionals within the colorectal unit, and a new group of patients and carers, to comment on the effects and effectiveness of the changes made thus far.

Maintaining collaborations demanded dealing with specific challenges of negotiating, inclusivity, comprehension, brokerage, and problem-solving. Time and resources continually needed to be negotiated as the requirements for time to be allocated to the research needed to be fitted into different organisations' pre-existing routines and priorities. Collaborations helped multidimensional networking and communication, both in finding ways to engage a wide range of people in the research and also to take multiple perspectives into account in formulating research priorities. However, the terms of collaboration – for instance the specific membership of the research team, which included senior clinical staff – may have impacted on ownership by staff groups who we may have assumed had been included in the process. However, it is clear that the diversity within the research partnership has helped enhance the range of people we have been able to reach and include.

Hamelin Brabant, L., Lavoie-Tremblay, M., Viens, C., & Lefrancois, L. (2007). Engaging health care workers in improving their work environment. **Journal of Nursing Management, 15*(3), 313-320. doi:* 10.1111/j.1365-2834.2007.00678.x**

This study sought to create an optimum work environment in a unit of care and one unit of services in a Montreal hospital. The steering committee (head nurse and head of service, the project co-ordinator from human resources, and representatives from among the health care workers) met seven times between October 2001 and April 2002 to identify work constraints, avenues for solutions to the organization of work, action plans, and evaluation tools. They collected data on constraints from employees anonymously and identified avenues for solutions and carried out action plans for these solutions over the course of the 2002–03 year. For each action plan, one member was responsible for liaising between employees and the steering committee, charged with forming a sub-team with some of the health care workers, and assumed joint leadership for the action plan together with the head of the unit.

Discussions highlighted that within the context of restructuring in the 1990s, budgetary constraints drastically changed the work environment leading to increased tension within work teams and job insecurity. The unit initiated a process for reorganization of work because we saw it as a springboard for modifying work relations and dealing with internal frustrations. From the start, involvement in the project was voluntary. Establishing opportunities for discussion facilitated the sharing of fears, feelings and new ways of doing things, and the steering committee's driving force and effort to depict a positive image of the future contributed to people's involvement. However, some were indifferent to the change and ignored it, continuing to work as though nothing was going on, and others were open about their disapproval manifesting their resistance throughout the change process.

Two particularly significant factors seemed to have contributed to the success of the approach to the reorganization of care and work. First, support from the management team: administration's commitment and involvement in the project, the fact administration explicitly stated why they wanted to proceed with restructuring, and projects that were aimed at increasing employee satisfaction, and head nurses' initiative, ability to work with people, active listening skills and desire for change helped instil the motivation needed to implement new practices. Second, the participatory approach: a unanimous agreement that administration, managers, employees and unions all need to be involved in implementing and maintaining change, and the emphasis on recognizing employee participation that contributed to the effective implementation of the project and increased employee satisfaction.

Two-way communication and recognition of employees' ability to make decisions were additional determinants for success. The preferred approach was to ask employees to move from expressing constraints to constructive problem-solving. By organizing meetings and frequent discussions at all levels, and introducing a journal in which employees could write their comments, the administration put into place the necessary tools for sharing information. All interviewees considered transparent communication within the institution to be essential, as it allowed for efficient coordination of tasks. These words speak for themselves: It is essential to present the project, not to impose it, to involve employees, also respect their choice to not become involved, value those who believe in the project, those who have ideas; follow up, make everything that we're doing visible, not play hide-and-seek;

talk about the positives and negatives, communicate the approach properly, from the outset not in hindsight.

Harris, L. E. (1998). 'Getting closer to a good thing?' Possibilities and limitations for empowerment in nursing: an Australian action research case study. (Ph.D.), University of New South Wales (Australia).

The purpose of this research was to work collaboratively with a group of registered and enrolled clinical nurses, at a large metropolitan hospital to identify the sources of their work satisfaction and the stressors they encountered, to work with them to devise strategies to reduce these stressors and this increase their work satisfaction. The nurse unit manager and nurses agreed to engage in the study. They met fortnightly with the researcher to determine the focus, and plan implementation and evaluation. Notably, much meeting time was taken up discussing how unsettled people were feeling because of the coming organisational changes

The researcher found that working together and deciding to DO something to achieve better working conditions was less problematic than encouraging participants to engage in reflection and assessment. Challenging participants appropriately and effectively to look at what they were doing, how they were doing this and in what ways their practise might be helping to perpetuate the conditions they found unsatisfactory was more difficult. A further handicap was that as an outsider the researcher had to spend considerable time and energy in selling the idea of collaborative research to nurses who were already overburdened. Other barriers to collaboration were time constraints and difficulties arranging meeting times.

As the project gained acceptance, communication between staff members improved (e.g., when differences occurred, some participants were more likely to tackle contentious issues with colleagues directly and immediately rather than not speaking out but carrying grudges), the ward climate became more open, there was an increase in innovative suggestions for improving patient care, they were more likely to let administration know when they had insufficient staff to run the ward safely, they were less prepared to accept the status quo, and the nurses were more supportive of each other. The outsider status of the researcher was an advantage as nurses perceived she could offer a different and wider perspective on nursing and their situation.

The researcher sent progress reports (previously checked and agreed to by the nursing staff) to the nursing administration and met regularly with the assistant director of nursing and the clinical nurse consultant to keep them abreast of the research. The senior nursing administration of the hospital had been supportive of the study in a practical sense by-passing red tape and granting provisional access, acknowledging progress reports by letter, although belatedly, and apparently implementing some of the recommendations in the reports. On the other hand, administration, as personified in the assistant director of nursing, was both accessible and helpful to the researcher and indirectly to the nurse participants in the project. There were difficulties disseminating project information to other nurses within the hospital, perhaps because the assistant director of nursing and clinical nurse consultant were no longer in position, meaning the new person responsible for organising research presentations knew little about the project. Following the project the researcher was approached by a staff member for advice on initiating an action research project. This research was carried out by hospital staff members with some consultation with this researcher as well as another.

Jones-Baucke, D. L. (1997). A qualitative study of the implementation of a system to increase nurses' use of standardized nursing languages. (Ph.D.), University of Washington.

Nurse participants in the intensive group attended nine 60-90-minute meetings where they learned about the problem understudy and worked to understand barriers to the use of SNL (standardized nursing languages) and theories relevant to constructing solutions and to examine the prototype model nurses had previously developed

During system installation and testing, the intervention developed by the intensive group was installed on the unit and some initial tests were performed by the investigator prior to having the nurses trial the system during real patient care situations. Next, the intensive group was trained to both increase their knowledge of SNLs chosen by them as relevant to their practice, as well as how to use the system to capture CNL. During this phase, the group was also taught how to access SNL definitions in texts placed on the unit. During the installation and testing phase, the intensive group used the input devices chosen during the first two phases of the project, to capture the SNL terms they deemed appropriate for their patient cares situations. Two barriers to use of SNLs were studied during this phase. The first, the nurses' ability to develop a schema of SNL terms relevant to their practice was assessed and second, the robustness of the SN terms chosen as relevant to practice in this setting were assessed. A debriefing session was held with each intensive group nurse after the first and third day of using the system during which nurses were interviewed by the investigator. Staff nurses realised that the barriers to SNL use spanned multiple categories all of which would have to be addressed by any intervention directed at increasing nurses' use of SNLs.

Nurses now practice and provide their services in a managed care environment. There are three power structures that make the difficult decisions required of managed care: healthcare purchaser, policy makers and the administrators of the HCOs.

This study has demonstrated that cogenerative learning, can be successfully used to formulate, develop, and implement information systems.

Joyner, K., & Mash, B. (2012). A comprehensive model for intimate partner violence in South African primary care: action research. BMC Health Serv Res, 12(1), 399. doi:10.1186/1472-6963-12-399

This study aimed to implement, evaluate and adapt a published protocol for the screening and management of IPV and to recommend a model of care that could be taken to scale in our underdeveloped South African primary health care system. A co-operative inquiry group implemented a published South African protocol for the screening and management of women experiencing IPV. The underlying assumption was that participants in the inquiry group would create new knowledge from their concrete experience; by observing and reflecting thereupon; by forming abstract concepts and generalisations; and by testing the implications of these concepts in new situations. The co-operative inquiry group consisted of five people, although participation varied according to availability. The first author engaged fully with the implementation of the protocol. Facilitation of the group process was primarily the responsibility of the second author, who did not engage with the implementation of the protocol. Four members, the study nurses, were engaged with implementing the protocol. Each co-researcher kept field notes to record key experiences, thoughts, emotions and reactions. Five co-operative inquiry group meetings were held over a period of 14 months for collective reflection and planning. The first author circulated a summary after each meeting.

The first author reviewed all transcripts and field notes and conducted a qualitative content analysis. A final meeting six months later provided an opportunity to reach consensus on the proposed model. Thereafter a consensus of the group's learning was circulated for validation. Feedback resulted in a modified protocol relying on a selective The inquiry group categorised the different components of the protocol into four broad areas: clinical, social, psychological and legal. The model that emerged was that the clinical component could be implemented by primary care providers, while the others required an IPV champion.

In retrospect, we were still too contaminated by an empirical-analytical mindset to fully innovate, experiment and implement change as part of the process. This dynamic was compounded by the fact that membership of the co-operative inquiry group was not consistent. Further, the group ownership of the inquiry process had to be held in tension with the first author's requirements for a doctoral study. The development of reflectivity also varied between co-researchers.

Khungern, J., Krairiksh, M., Tassaniyom, N., & Sritanyarat, W. (2006). Hospital quality improvement: a case study of a general hospital under the Ministry of Public Health. Thai Journal of Nursing Research, 10*(3), 191-200.*

In a general hospital under the Ministry of Public Health, study was conducted to find the meaning and practice of QI [quality improvement] perceived by staff, study the factors influencing the QI program, and to find feasible and appropriate methods for implementing QI in the general hospital. After building rapport for nine months, the researcher and the key staff conducted a situational analysis of the current QI in the hospital and presented it for reflection and evaluation with relevant staff. This activity made them self-organized into four working teams-documentation team, quality support team, intensive care unit team and administrators - to solve their problems through the process of PAR. The researcher worked with four self-organized teams in analyzing their situation of work by collecting data various sources of qualitative data and using it for reflecting in the groups and to plan for implementing to solve their problems and change some styles of working together.

The group decided to start from a paradigm shift in working that will enhance skills of system thinking, building a shared vision to see the future of the hospital and awareness of working together for QI. This type of activity made change by looking for natural leaders from every department in the hospital to work together. They required continuous capacity development to become change agents and facilitators who were willing to spread ideas to the whole organization. As facilitators, they opened up channels of communication by organizing forums new knowledge and concluding methods of problem solving and a shift from the result, paradigm shift of thoughts among the staff happened at every level, changed their perspective that quality improvement is the responsibility of only some staff to become the idea that it was a collective duty of all staff.

The paradigm shift changed the organizational culture at four levels: (1) Individual-Staff began working on QI in teams; (2) a new process for QI, which was based on participation to set vision and mission and translate them into an action plan, was begun. Indicators for monitoring and evaluation systems were created as well as working standards suitable for the hospital context to meet patients' needs; (3) a new group of change agents was developed to mobilize the feasible quality improvement of healthcare services continuously; and (4) the researcher learned the process of qualitative research, developed her own capacity to be a facilitator and mediator, and became more self-aware.

From working together, all participants discovered ways to develop an alternative quality improvement program appropriated.

Lauri, S. (1981). The public health nurse as a guide in infant child-care and education. Journal of Advanced Nursing, 6(4), 297-303.

The aim of the study was to develop the guidance of child- care and education that is provided by public health nurses at health centres for the parents of children aged 1-2.

The action model was evaluated in terms of documentation done by the public health nurses and in terms of their opinions. An analysis of the documentation indicated that the entries made in the components of the action model—needs, objectives, implementation, assessment — gained balance in the course of the experiment. In the opinions of the public health nurses, the conscious implementation of the action model also produced beneficial effects on their work as a whole. Most nurses began to pay more attention to the various areas of child development, and to explore the needs of the child more extensively as the basis of guidance. The objectives and programmes, in the opinion of the public health nurses, gave a direction to and a foundation for the guidance and counselling.

It was possible to improve the documentation and the action model of the public health nurses by means of action research. The results will probably lead to further development work.

The results indicated that the public health nurses regarded the methods which had been planned for this study as being applicable in their work. Moreover, an analysis of the methods proved that their properties were, in part, quite good. The use of the methods in the public health nurse's action model also improved in the course of the experiment. A wider use of the methods in the public health nurse's work will require further studies.

Laustsen, G. (2007). Promoting ecological behavior in nurses through action research. Communicating Nursing Research, 40, _552-552. doi:_10.1016/S0001-2092(07)60146-X

The study sought to: Involve nurses in research using Community Based Action Research (CBAR); to Pursue a CBAR project addressing nursing behaviors with negative environmental consequences. The unit's nurse manager and hospital's nursing research coordinator were supportive of the nurses' participation in the research process. The researcher served in the role of facilitator and co-participant. The facilitator and participants (the Green Team) collectively identified and decided on the specific community issue to be addressed in the research project. Red bag waste management and minimization became the issue and focus of the research project. The participants of the research team developed an action plan. Implementation of the plan's activities was then initiated by members of the research team. Specifically, using an action research design, the nurses investigated, evaluated, and promoted a change in the hospital's infectious waste policy. Commitment was demonstrated through the participant's willingness to attend meetings, follow-through on assigned tasks, and complete requested documentation.

Changes occurred at the individual, group, and organizational levels. Many of the individual participants reported changes in their awareness of the problems of nurse-generated environmental concerns, such as excessive red bag waste. Agents for these changes included discussions with the research facilitator, readings o f A ORN standards and journal articles, and personal investigations into the generation and disposal of hospital wastes. There appears to have been a diffusion of some of the changes experienced by the Green Team individuals and group to the organizational level. At the time of this writing, one of the Infection Control administrators assured that a new policy regarding infectious waste in the OR will be implemented in April 2005. Also, an Infection Control administrator found a changed culture in the OR. A ripple effect has been noted in the hospital in which the Green Team's emerging environmental awareness is expanding and influencing areas outside the OR/PACU. The hospital has recently announced a more focused effort on waste minimization by incorporating this topic into their strategic plan.

Positive aspects of participation in the CBAR as identified by the nurses were: A feeling of teamwork; Working towards a self-selected goal; A sense of empowerment through involvement in research; Recognition of the value of participant's knowledge and experiences; Development of team and individual knowledge and awareness opened their eyes to a new awareness and initiated or renewed a commitment to environmentalism promoted by their participation; revision of goals and redirection of the research activities, in the face of barriers.

The limitations or problems with participating that nurses perceived were: Frustration with slow pace of project; Difficulties with communication among team members; Difficulties with meeting scheduling and participation; Influence of stakeholders outside the OR/PACU nurse team.

The research team continues to pursue avenues of action towards the project's goal of OR/PA CU red bag waste minimization.

Lindeman, M., Smith, R., Vrantsidis, F., & Gough, J. (2002). Action research in aged care: a model for practice change and development. Geriaction, 20(1), 10-14.

The Well for Life project aimed to enhance the social and physical health and well-being of residents of aged care settings by empowering the staff of facilities to make change. The project had a particular focus on nutrition and physical activity. Participants identified practice improvement needs and a research question. Groups of staff in five settings meeting regularly over a three-month period. minutes were kept of each meeting and provided to every group member. Planned actions were followed through between meetings and reflected upon (and modified if necessary) at subsequent meetings where further actions were also developed. Group meetings provided an opportunity to reflect on actions and outcomes and to identify other possible solutions if necessary. The groups were facilitated by a project worker, independent of the residential setting, whose role included facilitation and group dynamic support, as well as practical support such as preparation of minutes, organizing meetings, arranging for expert input, and communicating with management. Staff communication processes were a major focus and included: development of a range of opportunities for staff discussions/ meetings, including scheduling changes, and an approach to educating staff to get to know the abilities and possibilities for individual residents.

Over the course of the project the group identified a number of issues relating to their research question that required further consideration, some of which they recognized were long term challenges. Identification of some of these issues prompted the development of strategies for change, however, it was also recognized that some barriers were unlikely to be overcome, at least in the short term. These barriers were raised with management at each facility. Indeed, the support or endorsement of management within the facility was critical to the success of implementing change.

Whilst knowledge and skill gaps were identified amongst staff, the provision of information and resources alone was often not enough to facilitate practice change. Staff training was commonly identified by participating staff as a key strategy for making improvements to the practice of resident care. In the action research groups, it was found that opportunities for staff discussion and problem-solving activities produced positive outcomes without necessarily the need for formal education programmes. Where staff education strategies were used or planned by staff, a broad range of strategies were considered including use of staff newsletters, workplace learning activities, strategic use of staff availability during change of shifts, creation of formal and informal opportunities for staff discussions, or time limited action-oriented activities.

It was important for work groups to accept that any member of staff may have suggestions and solutions to improve practice. All staff came to realize they had an influence on resident nutrition through their role in meals provision and the meals environment and all staff were able to contribute ideas to improve the overall practice of resident care at the facility. Providing staff with the opportunity to be 'listened to' helped to break down barriers and misconceptions about the skills and abilities of staff at different levels or backgrounds

Through the process of working together as a group, staff developed a commitment to working more as a team and were able to reflect on past practises that had discouraged this previously. Staff were able to reflect on their current practices, identify areas that could be improved, identify where there

were specific gaps in knowledge or skill and implement practice change. Feedback collected from group members indicated that they felt that the action research process was worthwhile. All staff agreed that 'the meetings were valuable because we could discuss different ideas'. Generally, staff welcomed the opportunity of raising issues and discussing ways that these could be dealt with. The possibility of holding regular staff meetings to allow for the same level of discussion and sharing of ideas as a result of their experience in this project was strongly recommended by staff.

Lucas, B., Cox, C., Perry, L., & Bridges, J. (2013). Changing clinical team practices in preparation of patients for Total Knee Replacement: Using Social Cognitive Theory to examine outcomes of an action research study. **International Journal of Orthopaedic and Trauma Nursing, 17*(3), 140-150. doi: 10.1016/j.ijotn.2012.10.002***

This 10-month project sought to examine the process of change when developing a preparation programme for patients awaiting Total Knee Replacement (TKR) Surgery in an outer London acute NHS hospital. The researcher initiated and facilitated the project which involved 'back office' activities of organisation and encouragement. A Project Management Group (PMG) was established consisting of orthopaedic consultants, nursing staff, physiotherapists, occupational therapists, managers and service, users who were patients who had had Total Knee Replacement surgery at the project site, and the university researcher who was a nurse practitioner within the organisation. Nine monthly PMG meetings held between January and October, with the aims of planning and reviewing the action cycles related to the development of the Knee Clinic and information booklet, and reflection on the progress of the project, including the change process. The researcher took notes during PMG meetings which were distributed to PMG members for checking and correction.

PMG members were involved in the action cycles to varying degrees. They worked within the meetings to plan, discuss, analyse and refine the test cycles. They decided which test cycles should continue and which should not be pursued. They participated in the test cycles themselves in various roles including data collection, participation in the Knee Clinic, and administrative tasks. Ultimately, the PMG developed into an effective team, demonstrating the behaviours of good communication and adaptability.

Some of the PMG members believed the project was well-managed and that this was a factor in its success. Some staff did not have high expectations of the project but nevertheless participated. It appeared that this participation modified their behaviour in that they continued to provide support to the Knee Clinic after the project ended. For the service users the project provided the environment for them to share and use their experiences of TKR surgery with staff and other patients. They helped to shape the direction of the project and changed the environment through the decision to set up a service user group for others to share their experiences of surgery after the project ended.

Malus, M., Shulha, M., Granikov, V., Johnson-Lafleur, J., d'Souza, V., Knot, M., et al. (2011). A participatory approach to understanding and measuring patient satisfaction in a primary care teaching setting. Progress in community health partnerships: research, education, and action, 5(4), 417-424.

The objectives of this project were to use a participatory research team of patients staff and researchers to (1) adapt an existing patient satisfaction questionnaire to the specific cultural and organizational elements of a primary care teaching clinic, (2) administer the revised questionnaire and use the findings as a tool for organizational improvement, with the ultimate goal of increasing patient satisfaction, and (3) ensure that all decision making involved patients and staff to empower them in the process of organizational change.

The active participation of everyone on the interdisciplinary team in questionnaire development and interpretation of results demonstrated the intention of the clinic's management to listen and to take seriously what the patients and staff had to say. Involving clinic administrators on the committee also helped them to understand the concerns and needs of patients and staff. Although the committee was created after the initial decision to study patient satisfaction and adapt a specific survey, it is important to note that committee members who joined were in complete agreement that this was a critical question for the clinic and were interested and committed to the process of adapting the questionnaire to best reflect the needs and expectations of the clinic's patients. Designing the questionnaire with an interdisciplinary committee was very enriching, but also a very time-consuming process. For example, reaching consensus on the exact wording of every question took a considerable amount of time.

Patient and staff members of the committee are co-authors on this paper and have started and will continue to be involved with the dissemination of both their experiences as committee members and the results of this project as participants at local and national conferences. Another iteration of the survey will be given in the next 6 to 8 months at the clinic to gauge the impact of the interventions. Before the follow-up, we plan to address issues surrounding response format, and test-retest reliability. However, in line with the spirit of this participatory research, the interdisciplinary committee will continue to be the key body in this continuing process.

Mash, R., Levitt, N. S., Van Vuuren, U., & Martel, R. (2008). Improving the annual review of diabetic patients in primary care: An appreciative inquiry in the Cape Town District Health Services. South African Family Practice, 50*(5), 50-50d. DOI:* https://doi.org/10.1080/20786204.2008.10873764

A co-operative inquiry group (CIG) sought to create and implement more effective practice teams of doctors and nurses in a Community Health Centre (CHC). The CIG consisted of the provincial and municipal facility managers, the senior family physician, a principal medical officer, the primary care nurse trainer and an administrative clerk. The group met over a period of nine months and was co-facilitated by two outsiders, a senior lecturer in nursing and a professor of family medicine. The involvement of the two outsiders had the effect of introducing new ideas and catalysing the process of planning, action, reflection and learning as well as amplifying feedback within the group. The CIG monitored the changes using observation and documentation of change in progress markers and success of strategies. Three central attributes of the CIG were openness to new information; attention to ambiguities; and allowing information to flow between and be generated equally by people who occupied different places in the CHC hierarchy. The planning and change process was partly dependent on the senior family physician and facility manager developing a good working relationship based on a shared vision. They were open and accessible to the health centre staff. It was important that once feedback was received and problems identified that management was responsive and proactive.

Successful change was dependent on creating a number of different formal and informal opportunities for interaction between staff members. These opportunities included the cooperative inquiry group itself, the monthly practice team meetings, a weekly family medicine meeting for the doctors, a weekly meeting of the nurses and the usual weekly staff meeting. These meetings allowed the CIG to dialogue with the rest of the staff members, with an ongoing exchange of information and ideas, both before and after the implementation of the practice teams. The nature of the communication and type of interaction was also important. People engaged with each other in a manner that was respectful, appreciative, built trust and included social bonding. Doctors and nurses often embarked on real relationships for the first time. The meetings also enabled practice team members to coordinate their roles and responsibilities and make these explicit. They gained a better understanding of each other and came to see themselves as a team more than separate professionals. The interactions built a shared vision and clear goals within the teams. Less effective team struggled due to the lack of these same qualities. Moreover, practice teams were vulnerable to turnover of health professionals: a certain amount of continuity in membership was required for people to build trust and adapt to each other's personalities and preferred working styles.

The CIG reflected on its own role in the change process and recognised that its style of interaction: (1) Was more reflective than the usual pragmatic action-oriented management meetings; (2) Constantly realigned people with the purpose of creating effective practice teams; (3) Enabled personal growth and change; (4) Fostered a more democratic and inclusive communication style between diverse professions and different parts of the hierarchy; (5) Created a group that crossed institutional boundaries – between municipality and province; (6) Built trust and shared understanding between key role players in the health centre; (7) Modelled a different facilitation style that could be transferred to meetings in the health centre.

Participation in the CIG enabled a wider variety of interpretations to be considered and contradictions were not immediately resolved by choosing one over the other. Participation of the members of the CHC in the envisioning, monitoring and adjustment of the evolving teams also fostered a sense of trust and ownership. The final form of the practice teams emerged from the flow and use of information in the CIG and CHC. The form and structure of the teams has continued to evolve as the health centre remains aligned to its shared core values such as continuity, efficiency and quality of care.

Mason, R. A. (2003). Action research: a hospital responds to domestic violence. Healthcare management forum / Canadian College of Health Service Executives = Forum gestion des soins de sante / College canadien des directeurs de services de sante, 16(3), 18-22. doi:10.1016/S0840-4704(10)60227-X

In 1996, a small working group initiated an organization-wide process to sensitize a hospital community to the relationship between violence and women's health. Researchers presented a literature review on domestic violence to the working group leading the group to look to their own hospital's practices regarding woman abuse and question if practices, as described in the literature, were in place. Accordingly, an "environmental scan" was undertaken. Interviews from Informants across the hospital made it clear that there was little recognition of violence or abuse as a health issue, no screening or any other interventions in place, no guiding protocols or policies and little education or training available. The report of these omissions surprised group members and they decided to modify their original objective and focus, instead, on improving practices in their hospital.

They developed "Philosophical Principles on Woman Abuse" and presented them, for feedback, to Senior Management and other committees within the hospital. The working group now had some legitimacy in the organization, paving the way for subsequent steps. Eight months later, the "Abuse by Intimate Partner and Former Intimate Partner" policy was approved for the organization.

One year after initiating the educational initiative, in-service training will have reached almost 1,000 of the hospital's 6,000 employees. Many program areas are developing protocols for providing care to victims of abuse with the help of working group members. The organizational culture itself is slowly being changed, and the group is moving closer to achieving its initial objective of a training institute for healthcare providers.

The action-research process of repeated cycles of action, observation and reflection created for the initiative both a framework and momentum for its activities. Combining action research with the principles of community development ensured that it was front-line staff who contributed the necessary expert knowledge and provided the impetus for meaningful action. Widespread support for the initiative resulted from the use of inclusive processes, including extensive consultation, multiple presentations to different staff groups, and broad-based front-line participation.

McKellar, L. V., Pincombe, J. I., & Henderson, A. M. (2006). Insights from Australian parents into educational experiences in the early postnatal period. Midwifery, 22(4), 356-364. doi:10.1016/j.midw.2005.09.004

This study sought to address some of the challenges facing contemporary clinical midwifery practice. Through the process of four Action Research Group (ARG) discussions a number of actions to enhance the preparation of parents for parenthood were proposed including resources designed (by midwives and parents through an action research process) to be used by midwives in hospital to support education and facilitate individual discussion with parents.

The ARG midwives demonstrated a sense of ownership that empowered them to embrace change in practice and enthusiastically promoted the actions in practice. They reported that they had effectively used the actions in practice and believed the actions were beneficial to midwifery practice. Further they agreed that the process of being involved in the ARG was invaluable and provided a means of support and both personal and professional development. The postnatal midwives, for their part, were less positive about the actions and identified a need to be more involved in the process of change in order to benefit more fully from the strategies implemented. Indeed, the ARG midwives had been involved in generating and refining the actions and understood the rationale underpinning the innovations. Unfortunately, as a small group within the midwives working on the ward, the ARG midwives were unable to successfully communicate their understanding or enthusiasm to enough of their colleagues.

The other midwives midwives commented on the difficulties experienced implementing the strategies in practice, particularly without the previous unit head, whose support had positively influenced their experience and motivation with regards to change. (The priorities of the new unit head did not include this study which resulted in some implementation difficulties and extended the time of the research.) They believed that it was important for the unit head to be involved and committed to the process of change. Also, it was acknowledged that the staff appeared negative and at times hostile towards new ideas. Many of the midwives on the postnatal ward clearly did not feel any sense of ownership of the actions implemented in the study. Rather, they saw them as an imposition. It is likely that the training provided for the ward midwives regarding implementing the actions was ineffective because the midwives had not recognised the potential benefit of the actions for their practice.

Mills, J., & Fitzgerald, M. (2008). Renegotiating roles as part of developing collaborative practice: Australian nurses in general practice and cervical screening. J Multidiscip Healthc, 1, *35-43*.

The research aimed to develop a new model of practice nurse service delivery within a multidisciplinary team. Six female registered nurses in general practice were originally recruited to undertake a pilot course of cervical screening education and training in 2005. These nurses were invited to join an action research group, which convened six reflective group meetings, one evening per month, over a six-month time. Each meeting lasted between two to three hours, with participants and the research team sharing a meal at the beginning of the meeting. The first author supported the action research group through: organising meetings, facilitating democratic processes, stimulating critical reflection, collecting data, and summarizing findings for the group. She was also responsible for the drafting of reports for the collective.

The group adopted a relaxed structure to the meetings. A catch-up session accompanied by food was used to break the ice at the beginning of each meeting. When the more formal work began, a summary of the last meeting was given and people were asked to provide an update of what had been happening. In the early sessions, the research team used creative means to stimulate participants' reflections by making posters or scrap booking. This was dropped as the issues for discussion became clearer and conversations flowed easily. A password-protected blog site was also set up and used intermittently by the group as a means of connection between meetings.

As a result of asking the question 'what else is going on here?' participants identified gender as a major influence on GP's patterns of referral for cervical screening. The participants felt disempowered in the face of trying to negotiate directly with female GPs who would not refer clients to them and showed little interest in understanding the potential change to their role. Practice managers (who had a different power relation with the female GP who was creating a barrier to change than the participants) were identified as potential champions, playing a key role in promoting open communication and new models of practice. Participants spoke of practice managers having a role as both a translator and mediator between nurses and other practice staff, and the general practitioners for whom they work.

Some of the characteristics participants identified which made them feel part of a team were: open communication, referral pathways, nurse led clinics, recognition of knowledge and skills, flexibility of working hours and opportunities for continuing professional development. Moreover, the process of 'putting yourself in someone else's shoes' was very useful for participants to start feeling less angry and disenfranchised and more proactive about finding potential solutions. The reflective process used in this action research group supported some members' levels of confidence and skills in negotiating a change in their role as cervical screeners. A final outcome from the action research group process was the formulation of a set of questions for general practice teams to ask themselves that aim to improve communication within multidisciplinary teams in order to promote interdisciplinary collaboration part of which is a clarification of team members' roles and responsibilities. The learning of the action research processes of critique and dialogue are an important outcome that will sustain the clinicians when the group disbands.

Mirza, M., Gossett, A., Chan, N. K., Burford, L., & Hammel, J. (2008). Community reintegration for people with psychiatric disabilities: challenging systemic barriers to service provision and public policy through participatory action research. Disability & Society, 23(4), 323-336. doi:10.1080/09687590802038829

Community reintegration (CR) services support people with disabilities who are currently in nursing homes to move back into the community. Seeking a better understanding of how to serve people with psychiatric disabilities in the CR program the program directors at the Centers for Independent Living (CIL) met with the researchers to discuss a research partnership. Both parties developed the project's aims: (1) to gain a better understanding of the experiences of people with psychiatric disabilities within the CR program; (2) to explore better ways of supporting them during the move into the community and during long-term community living and participation. Together, both parties decided to use a focus group to begin the data collection process. In collaboration with the community partners they developed two main purposes for the group: (1) to explore how people with psychiatric disabilities experience community integration and participation and the issues they face; (2) to discuss how CILs can better support people with psychiatric disabilities.

The content of the focus group was determined by reviewing previous literature on psychiatric disability and community reintegration, as well as through conversations with the community partners. The researchers invited one of the community partners to co-lead the group. Researchers analysed focus group transcriptions for themes and used these themes to guide subsequent actions. Results revealed a need for increased communication between various organizations that provide services for people with psychiatric disabilities in the community. In order to initiate a dialogue between the various entities involved in the provision and use of psychiatric disability services a community resource meeting was planned. It was agreed that opening up lines of communication between the various stakeholders could be initiated through the creation of an email listserv, where individuals could post information on advocacy efforts and other local and state-wide initiatives that they were undertaking to promote community services for people with psychiatric disabilities. This listserv is currently active and continues to be a forum where participants communicate and share ideas on an ongoing basis. Other steps recommended for developing a seamless service system included the creation of a clearinghouse of information and a toll-free number for service providers and consumers. While these steps have not come to fruition as yet, it is important to report that they are under consideration and being collaboratively worked upon. Throughout this project two prominent CILs, which have traditionally focused on collaborating with people with physical disabilities, are now forging links with the psychiatric disability community. An important outcome of this project is that the collaborating CILs will utilize the information gleaned from this project to actively modify their programming to better accommodate people with psychiatric disabilities. This project has laid the foundations for future bridge building between people with psychiatric disabilities and the broader disability community, particularly in the area of promoting community integration.

Mitchell, E. A., Conlon, A. M., Armstrong, M., & Ryan, A. A. (2005). Towards rehabilitative handling in caring for patients following stroke: A participatory action research project. Journal of Clinical Nursing, 14(3 A), 3-12. DOI:10.1111/j.1365-2702.2005.01143.x

The major objectives of this project were to (1) facilitate nurses in sharing their insights into moving and handling patients following stroke; (2) enable nurses to identify facilitators of safer moving and handling practice; and (3) empower nurses in collaboration with physiotherapists to direct changes in practice.

Three focus group meetings with participating staff (physiotherapists, occupational therapist, Registered nurses, enrolled nurses and nursing auxiliaries) in groups of four to five took place in February 2001. During these meetings, staff were facilitated in sharing their insights in moving and handling patients following stroke. All participants shared reflections on moving and handling individual patients. They also discussed their views of moving and handling risk assessment, handling guidance, and their knowledge of the particular needs of patients following stroke. The themes generated were recorded during each meeting and summarized in collaboration with staff, thereby giving them the opportunity to discuss the interpretation of the discussion and to correct any misunderstandings. Finally the data from all three focus group meetings were summarised together.

From the outset of this project, openness and collaboration were used to support staff and ensure that they were involved in directing and evaluating any proposed changes. The involvement of both nurses and physiotherapists from the outset of the project enhanced collaboration quickly. Staff were empowered through their active participation in the project.

The small-scale nature of the project created the conditions which ultimately led to significant changes in moving and handling practice. Clearly, participatory action research is a significant vehicle for delivering professional development in moving and handling practice.

Munn-Giddings, C., Hart, C., & Ramon, S. (2005). A participatory approach to the promotion of well-being in the workplace: Lessons from empirical research.* International Review of Psychiatry, 17*(5), 409-417. doi:https://doi.org/10.1080/09540260500238546

To develop collaboratively a strategy promoting well-being in the workplace which was context specific and sensitive to the workforce needs, staff were recruited to contribute to five participatory workshops at fortnightly intervals, each lasting two and a half hours. These workshops were used as building blocks to generate the data and suggestions that formed the final strategy document presented to senior managers. The strategy document contained staff 's perspectives on the core issues causing or aggravating mental distress at work and suggestions for short, medium and long-term actions to be carried out. These action areas also identified a process by which issues could be resolved and by whom.

Clearly the director saw the report as an unjustified indictment of the management input into the area of promoting well-being in the organization. In many ways it was an indictment, as the group was repeatedly highlighting the difficulties in communicating with management and what it perceived as lack of readiness to be more responsive. However, the strategies proposed moved away from blaming management to doable policies and practices. Ironically it was the facilitating team who on more than one occasion reminded the group of the positive steps undertaken by management. Reluctance by frontline practitioners and middle management to be in the same group was not overcome for the NHS trust.

The participants reported to an independent evaluator that they have found the process enjoyable and useful, though some have had reservations concerning specific aspects of the process (e.g., role play, which some found infantilizing). They felt able to actively participate in the discussions and the exercises; were listened to and their proposals were given the serious consideration deserved by the group and the facilitators. Despite challenges, the PAR process was felt to be an enabling one in which different types of expertise both within the AR team and the participating organizations were harnessed and utilized to develop a well-being strategy for both organizations. It helped staff to express and reveal issues around a problem and to generate realistic solutions.

The negotiations of both access to the participating organizations and the implementations of the proposed strategies were explicitly left to the facilitating team, and ultimately the team leader. With hindsight, this was to be regretted, as the negotiations focused on implementation this would have benefited from a much stronger sense of ownership by the participants.

Ngwerume, K. T., & Themessl-Huber, M. (2010). Using action research to develop a research aware community pharmacy team. Action Research, 8*(4), 387-406*.

The action research process reported here provides an account of Hull and East Riding Pharmacy Research Network and the pharmacy's actions and outcomes in fostering research awareness of medicine counter assistants (MCA), building research capacity and capability, enhancing MCAs confidence in their work when responding to minor ailment symptoms, and helping MCAs to provide customer-focused information using an evidence-based framework. They started by reflecting on their own practice and in doing so examined the reliability of the evidence base they used to give advice to customers regarding the sale of medicines. A need for more information was identified. Following a literature-searching workshop, co-researchers searched electronic databases for information relevant to their everyday work. They also learned to review papers, to interpret research outcomes, and to make the findings relative to their workplace. Evidence-based portfolios that could be shared with other pharmacies were built for various specific conditions and summaries of literature evidence found by the co-researchers on the active ingredients in different conditions were prepared by an author and made available to the co-researchers alongside original papers and other relevant materials.

The co-researchers had initial worries regarding researching academic databases, looking for published evidence, and changing their practices, but were able to learn new, and build on existing skills, collate a wealth of information on the products they were using on a daily basis, and gain a different perspective on their work routines. Through developing a picture of their current practice, the co-researchers became inquisitive about how they responded to customers' presentation of symptoms and the thought processes that took place when they made a recommendation. Prior to this project, the MCAs counter recommendations had been based on pharmacist preferences, personal experience, habit, training and manufacturer recommendations. Following the project, they included a consideration of the evidence base on the effectiveness of products.

The co-researchers, demonstrated a high level of commitment, shaped the development of the project, and gave periods of their free time to attend literature searching exercises and formal discussion sessions. They experienced increased self-awareness, thus confidence. They felt empowered by having gained the skills to acquire in-depth knowledge of over-the-counter drugs, and consequently, gained a sense of achievement. Discussions about challenges in utilizing this knowledge also made them aware of the difficulties in applying this new knowledge in practice and at the same time provided them with options about how to implement changes.

The action research process and consequences resulted in the development of a research aware workforce. Moreover, the co-researchers decided that they would like to hold portfolio building meetings twice or three times a year to discuss the latest research, to include any relevant findings in their portfolio, and to focus on more conditions. They found it a useful training tool, reaffirming their knowledge of over-the-counter ingredients. The portfolio collation and the plans to continue the co-researching process constituted a continuation of the action research process and demonstration that the pharmacy team had embraced this approach to research-based practice development.

Olsen, L., & Wagner, L. (2000). From vision to reality: how to actualize the vision of discharging patients from a hospital, with an increased focus on prevention. International Nursing Review, 47*(3), 142-156. doi:10.1046/j.1466-7657.2000.00024.x*

The aims of the Preventive Discharge project were to: (1) incorporate interdisciplinary and intersectoral collaboration into the development of preventive discharge, (2) develop, test and implement preventive discharge within a clinic of a Danish model hospital for preventive care, and (3) evaluate the process.

There was weekly contact between the clinic personnel and the project researchers. These encounters partly allowed new ideas to be shared on improving the tools and work procedures, and partly allowed comments, ideas and critique on the process itself to be collected. Between meetings, the researchers acted as messengers amongst the personnel, the Working Group and the Steering Committee. During this process, they developed the idea of involving the personnel in a Task Force, which would overtake further work in relation to the project. The formal and traditional leadership hierarchy was put to one side and all partners waited in anticipation of what would happen and when the next step would be taken. Dialogue, meetings and expectations led to agreement by the professional groups to identify a representative from their respective disciplines to participate in the Task Force. The researchers acted as leaders at meetings of the Task Force as well as an institutional memory for the group. The Task Force held intensive meetings, away from the daily practice of the clinic (substitute personnel covered the workload of the Task Force members). Meetings led to a critical revision of all elements within the project and emanated in new and concrete suggestions made for changes to procedures, competency sharing and interventions.

One of the challenges was to constantly create frameworks for interdisciplinary work, which built transparent trust, acceptance and a common timespan for overcoming any resistance to collaboration 'across' and 'top-down' within the health-care sector. This work clearly showed difficulties caused by rapidly shifting external demands and internal reorganization, change of personnel and the lack of experience in working with process-orientated, qualitative research and development. Using broadly defined terms (e.g., for 'discharge' and 'prevention') helped participants to find some common ground, despite their different backgrounds and mandates.

The demand for openness and co-operation supported the advancement of the project but simultaneously almost brought the project to a halt owing to resistance among the personnel, especially the nurses. In some cases, the project was perceived as having the function of a 'lightning rod' for conflicts. There were difficulties to overcome as participants shifted from the 'open space' of project meetings where relationships warmed up, to the practicalities of everyday work where the tendency was to 'stiffen' again. Working through dialogue within and around the project took time and brought about dilemmas in relation to reaching specific goals with a given time-frame and reaching the goal within the multidisciplinary practice of the clinic.

Orford, J., Templeton, L., Copello, A., Velleman, R., Ibanga, A., & Binnie, C. (2009). Increasing the involvement of family members in alcohol and drug treatment services: The results of an action research project in two specialist agencies. Drugs: Education, Prevention & Policy, 16(5), 379-408. doi:https://doi.org/10.1080/09687630802258553

This organisational change study sought to work with two specialist treatment teams to establish organisational standards for the engagement of families, e.g. that concerned family members are offered services in their own right, and that an attempt is made to involve a family member in all treatment episodes. The methods of evaluation were chosen in discussion with the participating teams.

The two service teams had felt very positive about being selected and about the idea of becoming 'demonstration sites' for family work, and had believed there was a lot for them to gain. As a first step, it had been agreed at the first joint training event that each person would begin by identifying one case with which some family work would be attempted. In practice this mostly proved much more difficult than expected. The first several months of the project was therefore a period marked by considerable frustration. One important issue that undoubtedly affected the project early on involved confusion about the project's objectives. It emerged that there existed two versions. One, mostly held by the researchers, and one held by many members of both Teams A and B. In fact, between Team A and the researchers there was a definite misunderstanding because the team's understanding of the objective was causing them considerable frustration. This misunderstanding was clarified and, although the tension between those two versions of the project objectives remained a talking point throughout the project, there was from then on greater understanding on both sides. The frustration of the early months gave way to feelings of achievement and a greater understanding of the proposed practice changes. They had found the individual interviews that a member of the research team had carried out with each of them to have been very helpful, helping them to realize how much positive work they were actually doing. For some team members there had been a complete turn-around from 'resistance' to 'excitement' about the proposed practice changes.

We believe the project achieved significant change in the desired direction in a period of just over two years: practice changes were achieved, and there was more joint work with colleagues. There was also evidence at both teams that the changes that had occurred were part of a process that would not now easily be reversed. On the contrary, they were part of an ongoing process that now had increased momentum within the teams and their wider organisations. An alternative conclusion, however is that the changes themselves are unlikely to be sustainable due to a variety of contextual factors. Change was difficult. Team members worked through a lengthy period of uncertainty and frustration, and it was only with persistence from all concerned that change started to occur after about a year. Perhaps the biggest lesson, however, was about clarity of objectives. The research team failed for a long time to make the proposed practice change objective clear.

Ottmann, G., Laragy, C., Allen, J., & Feldman, P. (2011). Coproduction in practice: Participatory action research to develop a model of community aged care. Systemic Practice and Action Research, 24(5), 413-427.

The research formed the first phase of a larger project designed to develop, implement, and evaluate a consumer-directed care model for older people with complex care needs in Melbourne, Australia. The method underpinning the model development was chosen collaboratively with representatives of all stakeholder organisations. From the outset, representatives agreed that the project needed to involve the entire spectrum of stakeholders. Importantly, older people using the services and their carers were central to the project and were involved directly in its development. Three working groups were set up: a service User and Carer Group (UCG), a service Provider Group, and an Advisory Group comprised of industry and government representatives. The aim of the UCG was to identify major gaps and shortcomings in the way care services were provided and collaborate with the researchers in developing a flexible model of community aged care designed to meet the needs and preferences of older people. The aims of the Provider Group and the Advisory Group were to identify model implementation issues and consider solutions to systemic and legislative barriers. The UCG met on 15 occasions, these being monthly meetings plus additional meetings as required. The other two working groups met when their input was required by the UCG and the researchers. The information obtained from these meetings informed the development of a draft service model.

It took approximately three months (3 meetings) for group participants to build sufficient trust to express their views and opinions. After reassurances from the researchers that all information was confidential and would not affect their relationships with their case managers, participants increasingly expressed their views. Useful discussions and successful outcomes were dependent upon the context and purpose of the meeting and the UCG role needed to be clearly explained at the beginning of each meeting.

UCG members identified gaps and weaknesses in services provision and guided the development of the service model from a consumer perspective. They codetermined meeting agendas and undertook research tasks (e.g., reviewing tools and documents to ensure their appropriateness for older people). Insights from the UCG members shaped the project and provided the basis for most discussions with the other two working groups. To ensure the needs and preferences of the consumer group would not be overrun by administrative and/or operational issues, researchers needed to take on the role of consumer advocate with other stakeholders. Restricted mobility and health problems were major barriers to group members' active participation and were overcome by telephone links to meetings, briefing members in writing regarding previous sessions if unable to attend and reimbursing them for costs incurred. The majority of the group's participants were committed to the project and invested a great deal of time and effort to ensure positive outcomes.

The team lobbied, unsuccessfully, the tier of government responsible for funding guidelines for more flexibility. Indeed, the opportunity to create a greater alignment between all stakeholders' expectations using a collaborative approach was missed because the key players declined to engage in an ongoing discussion. This communication breakdown led to a retreat to predefined positions and blame shifting between parties. UCG participants gained confidence and know-how over the course of

their involvement in the group which enabled them to express their needs and preferences more clearly.

Spalding, N. J. (2004). Using vignettes to assist reflection within an action research study on a preoperative education programme. British Journal of Occupational Therapy, 67*(9), 388-395.*

The aim of the action research was to evaluate and improve their preoperative education programme. The presenters1 were concerned that their lack of time and research experience could hinder the action research. Consequently, it was agreed that the author would coordinate the research and collect data, but that the process would be taken forward through the collaboration of all the presenters.

Three planning meetings took place by the presenters and the author prior to the data collection. At the first meeting, the research idea (to improve their programme) was clarified. It was agreed that the author would observe programmes as a means of data collection and these data would be sent to the presenters approximately one week in advance of team meetings. This was to try to ensure that the discussion in the meetings was more informed and to make good use of the presenters' limited time. The author was nominated by the presenters to chair all the meetings. In the team meetings, the data would be discussed and would be used to plan changes and to evaluate improvements.

In order that the presenters had control over their programme changes, they chose which data they wanted to discuss once they were at the meeting. They compiled a list of the issues that they wanted to address in turn. The presenters used the data that they had chosen to plan the changes that they believed would improve the programme. These changes were subsequently implemented and evaluated at the next programme. At the following meeting, the presenters reflected on their changes using all the data collected. These evaluations also brought about new insights from which the presenters realised new plans for change. A host of practice changes were determined by the presenters who volunteered to take responsibility for making the changes. The initial changes were about wanting to improve the programme organisation and they affected the way in which subsequent programmes were delivered.

In the author's opinion, her observational data were a valuable part of the process as the presenters could then reflect upon these observations and make decisions about future programmes. Team meetings facilitated reflection by providing a legitimate time for purposeful reflection, which might not have occurred otherwise. At no time during the research did the author perceive any reluctance by the presenters to make changes. They always appeared very eager to make the changes that they had decided upon as soon as possible, because they saw them as improvements to their service. In the team meetings, the presenters appeared confident and forthcoming in their reflections. The author also suggests that the presenters were empowered through gaining more knowledge from the data

[1] *Note: 'presenters' is the term used to refer to the health professional research partners.*

regarding preoperative education. At the end of the action research, the presenters believed that they had improved their programme in a number of ways.

Senesac, P. M. (2004). The Roy Adaptation Model: an action research approach to the implementation of a pain management organizational change project. (Ph.D.), Boston College, William F. Connell Graduate School of Nursing.

The purpose of this study was to conduct an action research intervention based on a nursing conceptual framework (The Roy Adaptation Model for Administration – RAMA). Nurse and the research collaborated in creating organizational change and in developing an understanding of the process of change. The organizational change project was the implementation of a Pain Resource Nurse (PRN) role in a community hospital. The steering committee selected three goals: to increase the satisfaction with pain management reported by patients on the Press Ganey survey; to increase the documentation of pain management in nursing records; and to increase the number of consultations provided by PRNs to staff nurses.

At the 10th meeting the steering committee presented an update report to the Director of Patient Care Services, the Director of Patient Care Clinical Resources, and the person responsible for leading the Hospital certification effort. The report was well received, and the dialogue gratifying for all, followed by an exchange of very warm and enthusiastic emails from participants and guests.

One Steering committee member felt their accomplishments were due to the structure of the meetings (the researcher focussed the nurses during the meetings and helped them to stay on track toward meeting their goals). Another commented on the contribution the group experience made in the developmental process toward active participation by staff nurses, and ultimately, shared governance (he felt the group "developed some kind of cadence or rhythm to the meetings" that he thought and hoped would be sustained. He said: "I think we've got some pretty interested people, and I think they like coming to meetings. They feel as if they're active participants. So, I think what actually happened is that it helped with the esprit de corps."

Siebens, K., Miljoen, H., Geest, S. D., Drew, B., & Vrints, C. (2012). Development and implementation of a critical pathway for patients with chest pain through action research. Eur J Cardiovasc Nurs. *doi:10.1016/j.ejcnurse.2011.05.004*

To develop and implement a critical pathway for patients with chest pain, a nurse was appointed as the coordinator of the group of health care professionals involved. Team meetings were organized every 3 weeks. Prior to each meeting an agenda with specific goals was drafted and distributed to all the different parties. At the end of all meetings action plans were produced and recorded and were subsequently used as a basis for monitoring progress at further meetings. Very soon in the process it became clear that not all members needed to be at every meeting. Therefore, the group decided on the involvement and invitation of members of the working group according to the stage of the process and the agenda of the different meetings (e.g., the development of the triage flowchart guidelines was too specialized for the whole team, thus, an intradisciplinary working group consisting of 6 cardiologists was instated and organized separate meetings apart from the mainstream efforts).

Based on results of initial work, several meetings with the interdisciplinary group were organized to identify problems and bottlenecks and possible actions to solve those problems. In this stage, the main difficulty was that a plethora of practical problems were put forward by different stakeholders and they expected them to be resolved soon. A structured and systematic approach in resolving these problems was therefore not always possible, but the final goal, to optimize the practice, was kept in mind while undertaking all the different actions. The coordinator facilitated this process and guided the group in researching its own practice.

It was a time consuming and sometimes difficult process since several people were involved and different visions existed. Using the principles of action research resulted in a democratic decision-making process and social dialogue, and in the end, consensus was reached. The group succeeded in adapting the best evidence in the practice of the service into a triage flowchart, treatment protocols and standing orders. Specifically, the critical pathway for chest pain patients was drafted, the triage flowchart was printed on a small pocket size chart so that the healthcare workers could always take it along when assessing patients, a detailed treatment protocol was proposed, and a document was integrated into the patient record that allowed physicians and nurses to work together within the same critical pathway document. Other major results of the pathway development process were CPU and hospital standing orders for the ED nurses, revision of physician and nursing medical records and the development of admission and discharge brochures for patients and their family admitted to the CPU. Certain problems could not be solved, due to financial or organizational boundaries of the work environment. Sometimes this was very frustrating and discouraging for people.

Through the establishment of dedicated multidisciplinary subgroups to get certain parts of the pathway going, a feeling of ownership of the different involved parties was reached. Special attention was paid to the involvement of the physicians since they are key players in the process. In the end, however, the ultimate goal was reached: the development and implementation of a comprehensible and workable chest pain pathway.

Smith, S., Dewar, B., Pullin, S., & Tocher, R. (2010). Relationship centred outcomes focused on compassionate care for older people within in-patient care settings. **International Journal of Older People Nursing, 5*(2), 128-136. doi:10.1111/j.1748-3743.2010.00224.x***

The aims of this study were: to understand how staff provide compassionate care; to identify and articulate what compassionate care looks like in practice and; to develop metrics and a practice development process that enables other in-patient units to enhance compassionate care.

Staff were encouraged to work alongside Senior Nurses to collect data. An inductive and collaborative analysis process was undertaken. The Senior Nurses (n= 4) and Lead Nurse from the LCCP met weekly. The Senior Nurses fed back data as they were generated in order that the process was transparent and to ensure participants had the opportunity to understand what was happening and engage in continuous discussion of the data. This took the form of reading the data, discussing its meaning, how it was fed back to staff, their responses and potential action projects that arose. As data generation progressed and increased amounts of data were heard by the group, common issues were identified, and key themes developed. The themes relate to processes that appear necessary for compassionate care to take place. These activities enabled opportunities for practice development to take place in near 'real time'. Timely and engaging feedback was an important activity, staff were constantly responding to multiple demands, therefore, feedback of data required to be both respectful and sympathetic to this.

The action project that emerged was to work with a family to develop an information brochure for families staying overnight in the hospital. Staff outcomes were gaining experience in working alongside family to co-create the service, enhanced understanding of the experiences of patients and relatives led to direct changes in individual and team practices. Outcomes for patients included enhanced quality of time spent with family and opening up conversations between families and staff. Outcomes for families involved enhanced access to relevant information and the opportunity to make sense of their situation.

***Smith, S. E. (1995)*. Dancing with conflict: public health nurses in participatory action-research. *(Ph.D.), University of Calgary (Canada). Doi:* http://dx.doi.org/10.5072/PRISM/16699**

A group of 12 nurses met 13 times for 2.5-3h, over a period of 8 months. They specified 2 areas of interest and investigated one in more detail. Mid-way through the process they implemented a first action and with a definite change in roles, planned and facilitated the remaining sessions. On a continuous basis the group reflected on its progress and learning and planned their next steps. The 13th session was a critical analysis of the effects of the group dialogues. Throughout the project, the researcher was responsible for systematically organizing, summarizing and returning information to the group. The nurses took on various roles with accompanying tasks at different points during the project: full participant; interviewee; resource person; session planner, organizer and facilitator; and interpreter of data. Following the project, individuals, sub-groups and the PHNs as a group became implementers of specific actions.

By the end of phase 2, most participants indicated that they felt better about themselves, supported each other more and were incorporating some of their leaning into their work in the community, but also believed that they would not be able to challenge the system in any substantial way. For some PHNs their increased level of comfort and trust with each other had extended into their work place, so that they could “bounce ideas off” of each other and have “more give and take.” For some, the process led to a greater confidence and in turn greater clarity about what everyone needs.

Facilitating factors included a process based on the group’s needs and starting points, a flexible structure, time for reflection, and patient attention to process.

The incorporation of new people made a significant difference to levels of trust between members (a need to start from square one to build a new relationship). The presence of management affected the safety and structure of the sessions, causing a ‘tightening up’. Absences of group members also had an adverse effect on trust and safety; the level of commitment of these individuals was questioned.

Tolson, D., Irene, S., Booth, J., Kelly, T. B., & James, L. (2006). Constructing a new approach to developing evidence-based practice with nurses and older people.* Worldviews on Evidence-Based Nursing, 3*(2), 62-72. doi: https://doi.org/10.1111/j.1741-6787.2006.00052.x

The long-term, and ongoing, aim of this participatory research project is to collaborate with practitioners and older people to develop a sustainable approach to enable the attainment of evidence-based nursing care for older people across the spectrum of care environments within Scotland. The initial concern was to lay foundations and articulate a shared vision. Cross-group consensus was achieved quickly within the first 2 study days using nominal group techniques. The agreed-upon project-development agenda was to identify selected aspects of nursing care and to develop guidance to describe "best nursing practice" and how to recognize it. Not unexpectedly, identifying key elements of gerontological nursing was complex. Much debate surrounded the concept of person-centeredness and while some doubts were expressed, this term was incorporated into their first version. Second draft suggestions were placed within an open access area of the college and all participants were asked to contribute feedback. The college members also requested that the new draft be placed on the public Website to seek critical feedback from interested parties. The second version of the definition encompassed the concept of relationship-centered care together with modified principles (e.g., commitment to relationship centered care, value of reciprocity; see http://www.geronurse.com).

Subsequently, to develop a procedural model for the evidence-based descriptions of best practice, the core group, in collaboration with project staff and steering group, drafted an intial model. Older people within the demonstration site were involved on an individual basis and through patient/resident groups, and participated in local steering groups. Feedback from demonstration site staff, and case studies summarizing the influence on practice and care experiences were prepared by nurses, care assistants, and older people. All of these sources contributed to the refinement of the statement before an external consultation exercise. Final statements were published by National Health Service Quality Improvement Scotland (http://www.nhshealthquality.org). Attempts to explore the model's conceptual aspects were abandoned as the consensus view was that this was "academic meddling."

Displays of mutual aid (support, shared problem solving) and group cohesion increased over time. Interestingly, even when a member could not attend a session, reading the archive allowed them to feel that they had participated and resume without disruption. There was no evidence that the group regressed throughout the year or lost productivity when new members joined, which was unexpected. Overall, it was concluded that in becoming a member of an effective group that the practitioner engaged in a transformational learning process. After two years, it was agreed that the work was beyond the scope of one group and a new community of practice was recruited to work alongside the core group. Over time, additional communities were formed, each with a specific and defined purpose. Older people were involved to varying degrees in the work of all groups, and an extension project included resources for 21 older people and family carers to form their own community. Preliminary analysis suggests that the older people who participated in the resources development have integrated computer use into their daily lives and valued the process used in the project. The

mixture of nurses from all areas of gerontological practice, academics and teachers together with the contributions of the expert advisor and the experiences from the demonstration site, resulted in a diverse evidence base from which the guidance was developed. Thus, this approach enabled the definition of ‘evidence’ to be broadened and the current dominant hierarchies challenged.

Wallis, M., & Tyson, S. (2003). Improving the nursing management of patients in a hematology/oncology day unit: An action research project. Cancer Nursing, 26(1), 75-83.

The project aimed to improve the time efficiency of chemotherapy protocol administration in a HODU [hematology-oncology day unit] in Southeast Queensland. In addition, it aimed to improve patient satisfaction with care, patient knowledge of the illness process and how to respond appropriately to the effects or side effects of treatment, and patient reports of symptom management. The staff of the HODU decided both to investigate the extent of these problems and to pose solutions. Their first step was to contact the clinical nursing research unit of the hospital and to obtain advice on research design. Later, the clinicians sought out collaboration with an experienced researcher to facilitate the project. The staff requested that the Chair of Clinical Nursing Research facilitate the process and take responsibility for parts of the project that required specialized research knowledge and skills. In turn, the clinicians would take responsibility for examining their practice, developing possible alternative actions, and collecting data. Both the clinicians and the researcher took equal responsibility for moving the process forward, developing data collection instruments, examining the results of ongoing data collection, reflecting on findings, and theorizing possible new approaches to care or actions.

The action research process began with a number of meetings between members of the team, in which the nature and scope of the problems with practice were explored and critically examined. A multidisciplinary team decided to develop a chemotherapy protocol manual (based on analysis of the data related to timing of the administration protocols and reflection on these results) that would explain all the requirements of each protocol and provide more accurate estimations of the time required to administer the protocol and care for the patient. In addition, the documentation used by the staff of the HODU was redesigned to allow for communication between all members of the multidisciplinary team. After the manual and new forms had been used for some time, the nurses thought that there were still inefficiencies in the system. Consequently, further phases of the action research project were designed to improve the patient appointment booking and staff allocation systems. The appointment sheets were redesigned, staff were nominated to be the primary caregiver for specific patients, a specific review clinic was set up for patients who were only going to see the medical staff (and not having a treatment), all the charts were relocated to medical records, and a computer-based chart requesting system for both the review clinic and the treatment clinic was implemented. Staff meetings were held in which the implications of this change in patient allocation for patient education were discussed.

In phase 3 of this study, the nurses had more time to spend with the patients educating them about their illness and the services available to them. This slightly improved patient knowledge regarding actions to take for problems they experienced at home. Consequently, as this research project came to an end, the staff were in the process of devising better patient education materials.

Waterman, H., & Grabham, J. (1995). Ophthalmic theatre nursing. Part 2: frameworks for practice. British Journal of Theatre Nursing, 5*(3)*, 5.

This action research project promoted posturing face down following macular hole surgery and aimed to enhance patient outcomes. HW first explored with staff who might be involved in the project whether the subject was a priority for them as well as their managers. There was clear acknowledgement that the care of these patients was an important issue, and some expressed interest in becoming involved in an action research project. None of the participants or co-researchers had any experience of action research. They appeared to trust HW as she had been a ward sister in the hospital and had recently carried out research in its inpatient areas. This led to a good rapport and relatively easy communication between group members. The group agreed to investigate the views and understanding of nurses and consultants about the care that was provided.

The group undertook a fact-finding exercise (literature review, visit to an ophthalmic centre that was at the forefront of care, investigation of the availability, cost and funding for posturing equipment, interviews with inpatient nurses and medical consultants) and results were presented at two workshops and possible courses of action were discussed. Subsequently, the action research group determined objectives and met monthly to consider how to achieve them. Summaries of the meetings were always circulated by HW in the form of a letter to all members. Members of the group were also kept in touch with progress through informal meetings with each other and HW. At the start of each meeting, participants identified topics for the agenda, which depended on the stage of the project.

The action research group analysed the data together. This gave opportunities to reflect, and discuss points and possible avenues for service or practice development, and helped gel the group. Data had a direct impact on staff serving to broaden understanding of patients' views on equipment and preoperative education, and it helped monitor the changes that were being put into place. The findings were fed into the action research group at regular meetings and reported at local and international conferences. The results informed nursing care. The action group decided to undertake a pilot randomized controlled trial (RCT) to examine the effects of two posturing regimes on patient outcomes for patients having macular hole surgery. The findings were presented to VR medical consultants and nurses at local and international conferences. This led to a more uniform approach to posturing regimes at the hospital.

One important issue that this action research project highlighted was the framing of the project from the needs of patients and not from anticipated or premature solutions identified by staff. It appeared that during the process of analysis, when the group heard what the patients had to say, this had a galvanising effect on the actions that were taken. We would argue that, once the project had reconfigured its interests to those of patients, more detailed and lasting suggestions were implemented.

The action research group was not insular in that it integrated closely with hospital management systems, for example, senior nurse and general management meetings. Some people who attended the action research meetings also attended these other meetings, and so ideas and actions from one influenced the other. This 'spreading and enveloping' of understanding of issues from all perspectives became vital in the acceptance and inauguration of change.

Compared with conventional research, action research has enabled us to go beyond a description of what it is like to carry out posturing, the causes of non-posturing and suggested methods to promote it. We have moved on to 'trying out' or 'experimenting' with different or new practices. With determination, commitment, and collaboration with patients and other healthcare professionals, we have learnt that we can reverse what was below-standard care to that which we feel able to present at conferences and publish.

Williams, A., Selwyn, P. A., McCorkle, R., Molde, S., Liberti, L., & Katz, D. L. (2005). Application of community-based participatory research methods to a study of complementary medicine interventions at end of life. Complementary Health Practice Review, 10(2), 91-104. doi:10.1177/1533210105279443

Described here are the methods of a randomized controlled trial of metta meditation and massage for adults with AIDS at the end of life. The collaborating partners in the development and implementation include the Integrative Medicine Center (IMC) at Griffin Hospital; the Yale Prevention Research Center (PRC); Leeway, Inc.; Yale University School of Nursing; and the Albert Einstein College of Medicine. Prior to the development of the study protocol, the IMC and Leeway were exploring ways to apply the integrative care model to the benefit of patients at Leeway. Once all collaborators were committed to the project, protocol development proceeded through a process of consensus.

The Leeway Ethics Committee, composed of staff and community members, highlighted the potential for residents' concerns about confidentiality and heightened sensitivity to subtle coercion to participate, which residents might experience. With the active involvement of the Leeway staff, community members, and the academic research team, the protocol was written to address social, structural, and environmental concerns of the study population. As such, it was tailored to the needs and circumstances of the study population and the site but did not sacrifice methodological rigor. At the suggestion of the Leeway staff, the meditation instructor briefly met individually with each subject at the end of Week 1 of the intervention to inquire about the subject's facility with adopting the meditation practice and to answer any questions.

The clinical staff from Leeway was responsible for communicating information about the study, recruitment, clinical screening, attaining informed consent, and coordinating the scheduling for the interventions and the outcome assessments. The Leeway staff helped allay any inherent suspicions the residents may have had of the academic researchers. Two senior Leeway nursing staff members, known to and trusted by the residents, were responsible for inviting residents to participate in the study and for obtaining informed consent, resulting in 97% participation by eligible residents. Prior to initiating screening and enrollment procedures, the Leeway nursing staff gave a presentation describing the study to the resident community. In this informal setting, without researchers present, the residents were comfortable, asking pointed questions about their role, responsibilities, and rights as study participants. The Leeway nursing staff took the time to personally introduce the PRC and IMC staff to each participant at enrollment, tacitly endorsing their involvement with the residents. Finally, the Leeway nursing staff guided the PRC and IMC staff in being attentive to noncoercion during interactions with participants.

The truly collaborative and active partnership between the community-based organization and the academic research center has been constructive. Establishing trust has been essential. Many factors have contributed to the project's success, starting with a research question that addressed an issue of interest to the community based organization as well as the academic researchers.

With mutual respect for complementary skills, many challenges have been creatively met. Flexibility built into the protocol as a result of the consensus-based development process accommodated most

challenges. Among the most difficult challenge was the disaffection of Leeway residents either deemed ineligible for the protocol or assigned to the control condition. All of the collaborating partners met to discuss how best to address the inequity and betrayal felt among those excluded from the intervention. After extensive deliberation examining the implications of each option, it became clear that addressing the residents' perceived needs would involve a compromise of the randomized controlled methodology or a need for additional funding support. The former was not acceptable to the research partners, and the latter option was pursued unsuccessfully. During dialogue unrelated to the study, Leeway residents requested access to a computer workstation for recreational and educational purposes. In a gesture of goodwill, and as an expression of gratitude from the academic researchers to the nonparticipants, a desktop computer was provided to the common area at Leeway.

Type 2- Random Sparks

ANDREWS, S., LEA, E., HAINES, T., NITZ, J., HARALAMBOUS, B., MOORE, K., ET AL. (2012) 72

BARKER, S. B., & BARKER, R. T. (1994) 73

BELLMAN, L. M. (1996) 74

BERINGER, A. J., & FLETCHER, M. E. (2011) 75

CLEMENSEN, J., LARSEN, S. B., KYNG, M., & KIRKEVOLD, M. (2007) 77

DAY, J., HIGGINS, I., & KOCH, T. (2008) 79

DEWAR, B., & MACKAY, R. (2010) 80

PINYOKHAM, N., FONGKAEW, W., CHANPRASIT, C., & SIMPSON, T. (2007) 82

ROBINSON, A., & MILLER, M. (1996) 84

VALLENGA, D., GRYPDONCK, M. H. F., TAN, F. I. Y., LENDEMEIJER, B. H. G. M., & BOON, P. A. J. M. (2008) 85

WHITEHEAD, D., KEAST, J., MONTGOMERY, V., & HAYMAN, S. (2004) 87

WILLIAMS, A. S. (2009) 89

WIMPENNY, K., FORSYTH, K., JONES, C., EVANS, E., & COLLEY, J. (2006) 91

Andrews, S., Lea, E., Haines, T., Nitz, J., Haralambous, B., Moore, K., et al. (2012). Reducing staff isolation and developing evidence-informed practice in the aged care environment through an action research approach to falls prevention. Advances in Nursing Science, 35(1), 3-13. doi:10.1097/ANS.0b013e3182433b27

In two Resident Aged Care Facilities (RACF) in Australia, 6 nurses, 3 domestic and maintenance services staff, an extended care assistant, a physiotherapy assistant, and a lifestyle and leisure officer formed a Falls Action Research Group (FARG) to improve falls prevention practice in their facilities. Working with academic partners, and critically reflecting on data collected in a preliminary investigation, the FARG agreed on a number of areas for action and developed and implemented 2 falls prevention practice interventions. The opportunity for FARG members to meet with their colleagues, from within their own facility and those from another RACF, fostered the development of new understandings about their respective workplaces and the conditions that shaped their practice. As such, the FARG members became more familiar with the contributions their colleagues, from other occupational groups, made to resident care. Another outcome was an improved understanding among staff of the rationale behind certain tasks. Participation also resulted in benefits outside the FARG meetings and the action research falls prevention activities. For example, the FARG members reported that they were increasingly being accessed as falls resource people by staff members who were not part of the group. Moreover, learning occurred in areas unrelated to falls prevention; FARG members indicated there had been lots of spin-offs. FARG members' collaborative working challenged the traditional hierarchical relationships between staff and opened up an opportunity to work in partnership, thus building capacity and empowering staff. Following this initial project, the falls group continued to meet and plans were made for ongoing falls prevention activities.

Barker, S. B., & Barker, R. T. (1994). Managing change in an interdisciplinary inpatient unit: An action research approach. Journal of Mental Health Administration, 21(1), 80-91.

In 1989 a three-year study began in a substance abuse inpatient unit in a large university teaching hospital in the UK, to generate a description of the substance abuse inpatient program, define and prioritize target areas for change, implement and evaluate change efforts, and provide an opportunity for staff participation and input into the change process. The nurse, medical, and unit directors, and other key staff members (e.g., admitting nurse) formed the team bringing nursing, medical, and psychosocial staff members' perspectives to meetings. Other staff members volunteered to form various subcommittees that developed and implemented changes (e.g., revision of criteria and procedures for monitoring patient progress in treatment, provision of written policies addressing major issues). All action followed a developmental process in which committees circulated drafts for staff feedback, thereby insuring that staff members were informed and invited to participate in all change efforts. The unit director's role was that of facilitator, providing encouragement, process monitoring, and feedback. The director relied heavily on group facilitation skills to achieve consensus among staff members. However, this consensus seeking did not occur overnight and actually involved several months of discussions. Through the group process, opinions were voiced resulting in all staff members supporting clinically sound changes that were consistent with the unit goals and philosophy. Changes were assessed by surveys and results were provided to the inservice staff to plan and implement adjustments and, then, re-assess. Administrators' support was readily forthcoming by including some in the action research process and by keeping others informed through the distribution of survey forms and committee and evaluation reports.

The use of the action research model employing staff participation increased the effectiveness of this multidisciplinary inpatient unit. Benefits include: a) an observed increase in staff morale, b) improved staff relations (e.g., good-natured teasing and humor compared to sarcasm and blaming readily observed in meetings prior to the project), c) lower staff turnover, d) more open intradisciplinary and interdisciplinary communication (e.g., staff members now openly support each other, ask for assistance from staff members in other disciplines, and collaborate on problem solving), e) new skills (e.g., team problem identification, decision making, cooperation, leadership), and f) staff appear willing to take more risks in making suggestions, confronting issues, and encouraging and supporting others.

Bellman, L. M. (1996). *Changing nursing practice through reflection on the Roper, Logan and Tierney model: the enhancement approach to action research*. Journal of Advanced Nursing, 24(1), 129-138. doi:10.1046/j.1365-2648.1996.15318.x

In 1996, 12 nurse co-researchers worked with the university partner to change nursing practice through reflection on the Roper, Logan and Tierney (RLT) model. Over 15 months, they sought to: 1) determine how nurses believe they apply the concepts of the model in practice; 2) identify problems associated with the clinical application of the model's concepts; 3) investigate the process and outcomes from reflection on practice; 4) identify patients' perceptions of their care; and 5) assess how innovation based on the model of care is perceived by surgical nurses and patients. Ward meetings were arranged on a continuous basis throughout the study to enable group reflection on practice, innovation identification, collaborative action planning, and patient and multidisciplinary team feedback. To enable identification of changes in practice relating to patients' psychological needs, the co-researchers agreed to discuss documented critical incidents at the group meetings. Collaborative participation was established with some members of the multidisciplinary team. The co-researchers' perceptions of the proposed initiatives were shared at the meetings. Positive reinforcement by patients provided the impetus for change throughout the study. Co- researchers experienced 1) personal enhancement (increased knowledge base, confidence, written and verbal communication skills, and patient teaching skills; a sense of achievement; and motivation to undertake further research); 2) patient centred enhancement (greater insight into the meaning of nursing practice); 3) group enhancement (greater team cohesion); and 4) empowerment (questioned the status quo, provided achievable goals, felt able to make changes). By facilitating and engaging practitioners in a self-determination strategy, their ability to challenge structured processes of social control and decision making, and change the status quo, was enhanced.

***Beringer, A. J., & Fletcher, M. E. (2011). Developing practice and staff: enabling improvement in care delivery through participatory action research.* Journal of child health care: for professionals working with children in the hospital and community, 15*(1), 59-70*. *doi:*10.1177/1367493510395639**

The overall aim of the programme was to improve coordination of specific aspects of children's inpatient care in 7 clinical wards. Two wards did not engage in the project, but the other 5 formed work groups of staff (comprised of nurses for the most part, though one group included administrators, a play therapist, and a doctor) that identified and prioritised aspects of coordinating care they wanted to improve. This made the project locally relevant, yet the issues identified were consistent with those of concern to the wider hospital Trust. Over a period of 17 months, the 5 work groups met with the University partner (facilitator) between 3 and 28 times, generally in the clinical ward areas (e.g., nurses' office) or, occasionally, at the University. These group sessions were supportive and open, with the facilitator ensuring all contributions were equally valued. Academic mentorship was arranged in order to provide advice and support through reflective supervision sessions.

The wards which fully achieved their aims were characterized by having a strong, identifiable, clinical nurse leader. This was the case even when the leader was a member of staff who was 'acting up'. The single most important indicator of full achievement of outcomes was that the work group members developed mutually supportive and trusting relationships between themselves and with the facilitator. Where these relationships did not develop this impeded achievement. Reasons for the failure of relationship development may have been related to the fear that involvement in the programme would expose weaknesses perceived by potential group members. Examples found during this programme included a lack of confidence in computer skills, in writing for publication and in giving presentations. Previous contact between staff and facilitator was found to be unrelated to the level of success of the work groups in that two groups who fully achieved their aims had not had prior contact, whereas one ward group who had been involved in the original research only partially met their aims. Contextual factors (e.g., management changes, ward mergers and closures) slowed or impeded project work on some of the wards.

The insight gained through this study was that activities the groups engaged in, as part of the PAR process, were all ways in which tacit knowledge could be brought to consciousness, allowing it to be accessed, expressed and shared. The tools used were flexible, to allow unpredicted (and unpredictable) insights to emerge, and also to promote equality of knowledge, which promoted the idea that different types of knowledge, and ways of knowing (here with respect to care coordination) are valid and valuable. An unexpected benefit was the staff's development of a new range of skills that they have been able to apply to other areas of their practice (e.g., data collection and analysis, practical ways to engage with colleagues in different departments to bring about changes in practice, process mapping, preparation and presentation of findings to a range of audiences). In the words of one of work group: "nurses are often asked to help with data collection for research, but many find it difficult to see the potential benefits. In contrast, using this 'partnership' approach to research gave us ownership of the process and, because we chose what to focus on, we could see its direct relation and relevance to our practice." This ward team now embraces change and feels confident it can make

a difference in the quality of care delivery to the children and their families. Note that this development of skills was coincidental to the programme process but central to the empowerment of the staff engaged in the group work.

Clemensen, J., Larsen, S. B., Kyng, M., & Kirkevold, M. (2007). Participatory design in health sciences: Using cooperative experimental methods in developing health services and computer technology. Qualitative Health Research, 17(1), 122-130. doi:10.1177/1049732306293664

The authors discuss their experience in conducting a PD [participatory design] research project intended to develop a technological and a related organizational intervention. Specifically, the aim of this study was to investigate whether video consultations in the home can support a viable alternative to visits to the hospital outpatient clinic for patients with diabetic foot ulcers. A number of workshops were conducted with the project group. They initially focused on identifying the problems involved in providing adequate treatment and care of persons with leg ulcers. In the beginning, the participants expressed prejudices toward each other; for example, some of the hospital nurses expressed concerns about visiting nurses' not taking diabetic foot ulcers seriously enough. During the workshops, however, the group reached a consensus as to how to understand problems with the current course of treatment. The intervention was iteratively developed and refined. During the pilot tests, a firmer structure for the consultation gradually evolved. A particular constraint from their daily work was the awareness that new initiatives could not be implemented within the existing budget. The emancipation from these constraints in the workshops, combined with carefully addressing the needs of the participants, such as food and drinks, made the participants feel great enthusiasm about the project.

Overall, the social and professional interaction between the researchers has created more nuanced and, consequently, better results. We involved representatives from several "user" groups in our study, including different professionals as well as "laypeople" (patients and relatives). This strengthened the diversity of the experience and knowledge base of the study. However, the diversity also added to the complexity of the research process. The patients and relatives, in particular, hinted about these issues in their interviews.

Clinicians, patients, and relatives all expressed enjoyment about the influence they had on the development of new solutions and better ways of handling the treatment and care of foot ulcers. As the health care participants (nurses and physicians) were dealing with problems from their daily work in a new context that did not have the normal time and resource constraints, they were able to review their work in a more creative and critical way. The participants expressed how they had enjoyed being heard and taken seriously. In fact, they felt that they had a lot of influence on the process and thereby on the results from the research. The participants described how the workshops and the other activities in the project had helped them obtain a renewed perception of their work and situation. They expressed that they could use this in their daily life to be more positive and creative in their way of thinking. More important, the participants also suggested that they had already benefited from the project in their present work situation. Visiting nurses emphasized that participating in the project had increased their professional skills, by receiving information and instructions from the hospital. The patients and their relatives had a slightly more critical attitude toward the process. They also said that they had enjoyed being part of the creative process and felt ownership of the products; however, they did not show the same degree of devotion or enthusiasm.

The patients had difficulties in understanding the reason for some of the activities, especially at the beginning.

We were very surprised to learn how participation had actually changed some of the participants' ways of thinking in general and how they used these new perspectives in their lives. The patients saw the possibilities for new ways of organizing the course of treatment in the future, thereby providing them with a better quality of life. For the professionals, the new ways of organizing the course of treatment affected their job satisfaction because of a more suitable collaboration between the involved clinicians, leading to a more coherent course of treatment. In our study, we experienced how playful and creative interaction in a group of participants released important knowledge in all the participants, enabling them to combine their opinions in a productive process to solve a significant clinical problem.

Day, J., Higgins, I., & Koch, T. (2008). Delirium and older people: What are the constraints to best practice in acute care? International Journal of Older People Nursing, 3(3), 170-177. doi:10.1111/j.1748-3743.2008.00115.x

This action research project was designed to improve the care of older people at risk of delirium. University partners were invited to conduct this study in an acute medical ward where delirium had been identified as a clinical practice problem by clinicians and the nursing unit manager. Thirteen weekly PAR group meetings were held between March and July 2007 and facilitated by the university partner whose role was as the overall project coordinator. At the first meeting, participant clinicians were encouraged to talk about collaborative group processes, group sensitivities and expectations. Later 'group norms' were agreed including having a voice, respecting others who speak, listening even when interests and values were in competition, to use of non-judgmental language, and that conversation within the group meetings be strictly confidential. These values have been sustained beyond the life of the project. It was important to meet regularly to keep the interest and energy of the group up. Academic staff provided clinicians with summary notes of weekly discussions. This was invaluable as it helped to reflect on discussions and ignited new thoughts and deliberations.

In the initial group sessions, each participant was invited to tell a story about a patient experiencing delirium. Using the stories and the literature, clinicians reflected on the issues and explored possibilities for action that could be implemented. The story telling approach encouraged clinicians to interact with each other by sharing ideas and stimulating new insights into the topic. The group took action to make the staff 'delirium aware' and developed a delirium alert protocol. Because the staff saw value in this research project they were able to engage other staff to also participate and follow through with the implementation of the protocol. When the research team returned to the ward 9 months after the study commenced there was a sense of calm amongst clinicians and in the ward environment. There was evidence that practice had changed. Staff claimed ownership of the protocol, its attributes were user friendliness, accessible language, clinical relevance and most importantly, no formal documentation had been required as 'staff hate additional paper work'. Champions had been self-selected from PAR participants and fellow staff members were educated about the protocol.

Although PAR group members found it challenging to meet their commitments to their patients and other staff since they were 'off the ward' when attending PAR group meetings, they found the gains from participating in the project exceeded their expectations. Their understanding of delirium improved; their practice is now guided by the evidence; they understand and appreciate the real and practical ways a research project can lead to better patient care and nursing practice, and some want to be involved in more research projects; they now have greater confidence in identifying patients at risk and those already in delirium; the nursing care they give has changed; they gained new insight into what they could do to change their practice; they feel good about themselves and what they achieved; and finally, the project gave staff members a 'platform' for demonstrating their skills and knowledge so that they learned a lot from each other and this is ongoing. Notably, education was mutual; the research team became familiar with ward practice.

Dewar, B., & Mackay, R. (2010). Appreciating and developing compassionate care in an acute hospital setting caring for older people. **International Journal of Older People Nursing, 5*(4)*, *299-308. doi:*[10.1111/j.1748-3743.2010.00251.x](10.1111/j.1748-3743.2010.00251.x)**

This work is part of the Leadership in Compassionate Care Programme which is using an action research approach to embed compassionate care in practice and education. This study actively involved older people, staff and relatives in identifying strategies to promote compassionate care in acute hospital settings for older people. The discussions were led initially by the lead author but later by other staff. Facilitation supported discussion and debate, but also made staff more confident and prompted them to initiate and lead conversations. Staff were asked to invite patients and families to take part in interviews to share their stories of what it is like to be in hospital. If the patient had given permission, the story was shared with staff members and discussion was facilitated to articulate key learning and actions. The stories were linked with other evidence and put into the context of the culture so that meaningful learning and action can be facilitated.

Data were continually fed back to participants through various means including reading quotes or observation excerpts at staff handover and displaying emergent analysis in key areas in the ward for comment and critical discussion. Data analysis used a participative approach (initial description of the data; crystallizing the core messages in data extracts; considering these in relation to all other data; reflecting these back to participants; and creative synthesis and corroboration of the themes). Positive-care practices were identified from the stories of patients and families and were summarised into meaningful statements. These statements were matched with images that helped communicate them to staff. The images were displayed in digital photo frames that were placed in areas where staff could meet and discuss the statements. Statements were adapted in light of discussions and actions agreed.

Finding out what mattered to individuals, through their stories, helped to challenge existing practice. Staff questioned and subsequently changed their practice, showing a degree of humility and recognizing that the 'staff' do not always know what is best. The changes have not only focused on practical solutions; the process has also provided a platform for discussing some of the more complex cultural aspects that contribute to the delivery of compassionate care. Staff exhibited an increased awareness of the importance of the need to create opportunities where patients and their families are routinely asked to share how they are feeling about the experience of being in hospital. Staff now questioned how patients felt about their care and became more comfortable about sharing them their own thoughts, enabling them to explore issues that previously would have been glossed over. They both supported and challenged each other to consider new ways of communicating that became a legitimate and important part of the way in which staff interacted; they now worked together more collaboratively. Hearing the positive, special and often invisible aspects of practice, has had a significant influence on staff and has enabled them to more clearly articulate to others the compassionate caring acts that they carry out and value. The project also clearly enhanced opportunities for collaboration between patients and families. 'Working together to shape the way things are done' made people feel involved and empowered but also meant taking risks. This required an environment that: encouraged people to be open and honest; acknowledged the constraints under

which they operated; and helped them to find potential solutions. With such support staff, patients and families were better able to accept compromises based on a full understanding of the situation

The extent to which staff felt comfortable to challenge practice depended largely on the support they received from colleagues and senior staff, which increased as the study progressed. Thus, as staff and patients shared their true feelings a more responsive, connected and stronger relationship ensued. Developing person and relational knowledge involved people making connections, engaging emotionally and reflecting on the insights gained to better understand the experience of the 'other'. These processes caused uncertainties and had the potential to create tensions but also led to opportunities to challenge existing practice and to forge more responsive relationships between all groups.

Pinyokham, N., Fongkaew, W., Chanprasit, C., & Simpson, T. (2007). Development of a program for enhancing nurses' capacity to wean patients from mechanical ventilation. Thai Journal of Nursing Research, 11*(4)*, 281-293.

The aim of this study is to develop a program for enhancing nurses' capacity to wean patients from mechanical ventilations. A core-working group of 9 CCNs [critical care nurses] was thus empowered to plan and to act in creating social change through a program for enhancing the capacity of the nursing staff in weaning a patient off a ventilator. The researcher arranged a workshop for the core working group and administrators on weaning problems, possible nurses' roles in solving these problems, and a plan to solve the problems effectively. The final consensus was established in the workshop: they agreed that problems should be solved by increasing their knowledge about weaning and the necessary knowledge for weaning patients effectively for all CCNs. After attending the workshop, the core working group made a commitment to work on developing and implementing the program with the researcher's facilitation and support from their administrators.

During the first group meeting, facilitated by the researcher, the core working group reviewed conclusions from the workshop, elected a chairperson and shared responsibilities to facilitate the program developing process. They planned for group meetings on an alternate weekly basis to develop a tentative program and discuss their ideas about the program's contents. As a facilitator, the researcher's activities in this step were empowering by educating, reassuring, supporting, encouraging, helping and providing resources for developing the program. Some core working group members felt unconfident about taking role as program developers, complaining that they had heavy workload and difficulty in reading the materials written in English. They also had limited spare time and felt exhausted after work. Thus the researcher took a 1.5 month break to seek a solution. This break allowed the participants of the core working group to reflect about their participation in the research project as well. After the following meeting discussion, they were willing to continue their role as program developers. However, the core working group asked the researcher to confirm their understanding of the reading materials, and divided into three subgroups with the aim of sharing responsibilities to prepare content of the program based on their expertise. During this step, the researcher visited the members of the core working group regularly to encourage and appraise the program's progression. With the core working group's collaboration, a tentative program was formulated. Strategies for program implementation were designed to enable all CCNs to attend. The program was reviewed and refined based on the participants' suggestions. The final draft of the program was prepared for presentation to all the stakeholders.

The PAR process and the researcher's facilitation empowered the core working group to develop an educational program with full participation of almost members of the organization throughout the program development process. The core working group enhanced their research experience and learned how to develop their capacity for planning and decision-making concerning weaning through seeking knowledge independently. They became conscious that consistent self-development is necessary for enhancing their capacity and the development of nursing practice. The administrators agreed that the program benefited their units in term of increasing nurses' awareness, knowledge for improving practice, and showing more self-confidence when providing care for patients. Finally, the

other participating CCNs gained knowledge, improved their clinical skills, and gained confidence for actively participating in decisions related to weaning.

***Robinson, A., & Miller, M. (1996). Making information accessible: developing plain English discharge instructions*. Journal of Advanced Nursing, 24*(3)*, 528-535. doi:10.1046/j.1365-2648.1996.22113.x**

The study began when a ward nurse had concerns about the large numbers of telephone queries from parents. She discussed these concerns with her nursing colleagues, many of whom believed that several of the discharge education practices on the ward were problematic and that something had to be done. These nurses formed a participatory action research group to explore and address the issues relating to discharge education on the ward.

Over a period of 6 months, to identify problematic areas in discharge education on the ward, the group members began by sharing accounts of their discharge planning and education experiences and documenting the frequency and content of telephone queries from parents. Based on the Analysis of the telephone queries data, the group decided to focus their research on the discharge education and planning processes. To gain insights into these processes, the group members developed a questionnaire which they administered to nursing staff on the ward. Findings led the group to decide to focus on the consistency of information given to parents. To develop a home care discharge instruction sheet, the group members worked iteratively, analysing and critiquing its topic, purpose, and effectiveness, as well as the language used (jargon vs. plan language) and its purpose and effectiveness.

Through their research work, a group of clinical nurses developed a discharge instruction sheet that consisted of a set of instructions that were clear, simple and precise, and devoid of jargon. Through participation in the project, all group members gained new and valuable insights into the ways that nurses accept assumptions about the appropriate language to use with patients, and the importance of recognizing technical jargon for what it is — an often obscuring and alienating discourse. Unfortunately, funding cuts, which also resulted in ward closures, meant that the study could not continue.

Vallenga, D., Grypdonck, M. H. F., Tan, F. I. Y., Lendemeijer, B. H. G. M., & Boon, P. A. J. M. (2008). Improving decision-making in caring for people with epilepsy and intellectual disability: An action research project. Journal of Advanced Nursing, 61(3), 261-272. doi:10.1111/j.1365-2648.2007.04477.x

This study aimed to improve decision making concerning risks in people with epilepsy and intellectual disability and empower participants to sustain this improvement. The primary researcher informed the management about the methodology of action research, and management proposed units for participation, implicitly agreeing with the goals and chosen method. The primary researcher facilitated the change process. Two teams in two units participated. They decided that there should be dissatisfaction about cooperation, actions or goals, they should immediately inform each other and look for a solution; however, this situation did not occur. The desire for transparency in decision-making and communication became apparent. They decided to make improvements in work methods related to risk-management the goal of the whole project. Within the action cycles, this analysis process was carried out together with the nursing staff by using the computer program MAXQDA. The findings of each part of the study were discussed in the action research group meetings and, on the basis of this reflection, new goals were set. Actions and goals were closely related to problems encountered in daily practice and to different aspects of decision-making. This sometimes led to goals which were broader in focus than only decision making.

The first few action research cycles contributed to nursing staff becoming more fully aware of the phases in risk management they were more able to adjust their behaviour towards risks and clients, leading in turn to more client-oriented care. In the final few cycles, the teams worked toward improving practices and implement guidelines. At this point, the researcher's support became less essential. At the end of the last action cycle the teams evaluated the project. Caregiving had become more client-centred, and decisions were now made after a more systematic analysis of the clients' situation. Increasingly decisions about risk were made not only in reaction to incidents but that the clients' broader situations were included in the analysis. Nursing staff increasingly sought measures suited to a client's wishes in the situation at hand and ways of reducing the restrictive aspects of protection.

Six months after the last action research meeting decisions were no longer made by staff acting alone, but care was taken to involve other staff members. The limited possibilities for sharing and discussing viewpoints while at work led to the development of a written system to discuss choices and proposals. A methodical cycle to team meetings was being applied, which led to more effective and goal-centred meetings. The guidelines were being used. They had been discussed with representatives and included in the care plan; they had become a context for caregiving in daily practice and had clarified the limits of responsibility of those involved. Furthermore, the improved internal communication was attributed to the action research project.

Analysing client problems as a team, seeking improvements and sharing decision-making had resulted in teams which were less task-oriented and more coherent in observing and evaluating client problems. Nursing assistants felt that their viewpoints were now heard on a structural basis and felt an increased autonomy. Team reflection on decisions made also stimulated their personal reflection

and increased objectivity. Reflection had become a more integral part of the job, participants had gained insight in analysis and evaluation of the risks to which clients were exposed.

Whitehead, D., Keast, J., Montgomery, V., & Hayman, S. (2004). A preventative health education programme for osteoporosis. Journal of Advanced Nursing, 47(1), 15-24. doi:10.1111/j.1365-2648.2004.03058.x

This paper reports an action research project with one hospital osteoporosis service. The aim of the project was to assess the current service, identify areas where change was needed, and evaluate the implementation of such changes. Qualitative data were generated primarily through regular PAR group meetings involving all research participants. The intention of the groups was to establish a mechanism for opinion formation on the issue of organizational programme reform. The PAR group sessions were supplemented with unstructured personal reflective diary accounts from each participant. The PAR group meetings were conducted every 1– 2 months. Each session lasted between 60 and 90 minutes. Detailed real-time notes were taken throughout each group session. Open-ended questions and probes elicited further discussion and elaboration as required. The PAR group meeting sessions enabled the group to review progress, identify emerging issues and agree plans of action. Each new group session started with a review of the notes of the previous session. The participatory nature of the research meant that each member of the group took it in turn to take the key role of moderator.

General feedback from both ward staff and clients in the designated settings proved to be overwhelmingly positive. All clients and staff who commented unanimously supported the need for a preventative osteoporosis programme and reported that they had benefited from the information and services that it provided. Research participants were encouraged and motivated by the positive response. This, in turn, prompted further activity, such as setting up specific referral processes for clients wanting more information from the Health Information Centre. The level of information uptake in this study was encouraging. In both the care of older people and orthopaedic clinical settings, all 300 information packs initially produced for each area were utilized.

One of the main findings of this study did not directly relate to the issue of osteoporosis prevention, but to the collaborative nature of the study. It proved to be a liberating and enlightening process for the research participants, despite any initial reservations. The empowering action research framework meant that the research team were able to explore their power relationships with each other. The difficulties that may be faced by the action researcher, such as organizational political tension, lack of managerial authority, unequal power relationships and disempowered participants were evident to varying degrees, but not insurmountable, in this study. Each participant had the ability and authority to challenge existing structures without too much threat and possessed a degree of autonomy to implement effective service reform. The quickly established common aim and purpose of the research group within an action research framework, i.e. desire to widen the scope of the osteoporosis services to incorporate health-promoting preventative activity, helped to promote unity between the participants working towards this chosen end.

A preventative health promotion programme has been set up that has achieved a significant number of outcomes. Its impact is still ongoing. Despite the fact that the study has been completed, one of the participants is still actively involved in the day-to-day running and activities of the osteoporosis service and is seeking to extend the range of preventative services on offer. The final reflections of group participants uncovered a number of positive outcomes with regard to the study and its

processes. All participants were pleased with the level of organizational change that had been achieved, which surpassed their initial expectations. The service had seen a number of beneficial changes over the lifetime of the study. All participants indicated their enthusiasm for action research as a supportive framework and all expressed their willingness to use it again. The only negative outcome was that a few of the participants were disappointed with the level of feedback from, interaction with and change by clinical staff. A further study is planned that will measure and evaluate the direct impact of changes in the osteoporosis service as it has continued to expand. This time the study will be aimed at the clinical staff and client interface and will probably also adopt a PAR design.

***Williams, A. S. (2009). Making Diabetes Education Accessible For People With Visual Impairment*. The Diabetes educator, 34*(4)*. *doi:*10.1177/0145721709335005**

The purpose of this project was to make the patient education materials and programs of the Diabetes Association of Greater Cleveland (DAGC), a voluntary local diabetes organization, accessible to people with visual impairment. The process was to hold regular meetings of the Planning Group, comprised of the five AAG [action and advisory group] members, the four DAGC [diabetes centre] staff members, and myself. The Planning Group met at the beginning of the project, and at least monthly throughout the implementation phase of nine months. The purpose of these meetings was for the Planning Group members to identify specific actions they could take to increase the accessibility of DAGC's programs, develop plans to implement those specific actions, and, when possible, implement the plans. Each meeting and the time that followed it represented one of Stringer's "Look—Think—Act" cycles. At times, the entire Planning Group, or parts of the group, met outside of the monthly meetings to work together or to gather information. Meeting notes were provided to the participants in their preferred format (large print or audio) and were read aloud at the beginning and end of each meeting. Finally, as an acknowledgement of the value of the VIP's expertise, they were paid for the time they spent on this project. Evaluation of progress was a continuous feature of the Planning Group meetings.

The PAR process provided a setting for extended direct contact and cooperation between the four DAGC staff members and the five PVID. This contact enhanced both the products of this project and the process of discovering ways to meet the needs of PVID. All PVID and DAGC participants reported profound learning and transformation of their relationship with each other. AAG members also found being paid was not just a monetary benefit but that it boosted their self-esteem and made them feel valuable. AAG members mentioned that participation gave them an opportunity both to communicate their own experiences of living with diabetes and visual impairment, to be heard by a group of professionals, and to develop a trusting relationship with those professionals, increased their skills for expressing themselves, and learned more about diabetes. An unexpected result of this project was that three of the AAG members were encouraged by this project to find ways to use their talents in other situations (help other people who are experiencing new vision loss, advocating for the staff of a rehabilitation facility to have an in-service presentation on modern diabetes car, try a similar educational experience with a different organization). For DAGC staff members, advantages of working on this project included earning about visual impairment and the opportunity to network with a new group of organizations.

AAG members stated that they had felt anger and suspicion of the DAGC staff members at the beginning of the project, but that those feelings changed as the DAGC staff members demonstrated their willingness to listen and change. The major disadvantage of the PAR process for DAGC staff was the amount of staff time spent in meetings (about three hours a month for 10 months for two or three staff members).

From my perspective as project facilitator, the strengths of using the PAR process clustered around the following themes: 1. Authenticity of the participation of the AAG members. 2. Communication by the AAG members of aspects of their experience that were unknown to the sighted participants. 3. Increased linkages between DAGC and organizations concerned with low vision and blindness. 4. Encouragement of increased assertiveness and advocacy by the AAG members. 5. Program innovations

that provide an opportunity for systemic change focused on including disabilities in diabetes education standards.

Wimpenny, K., Forsyth, K., Jones, C., Evans, E., & Colley, J. (2006). Group reflective supervision: Thinking with theory to develop practice. British Journal of Occupational Therapy, 69(9), 423-428.

To support the development of the skills and tools needed to integrate a conceptual practice model, three teams each met with the occupational therapy academic for group reflective supervision for 2 hours once every 4 weeks for 12 months. These sessions focused on addressing barriers to adopting MOHO, re-examining practice in the light of theoretical constructs of MOHO and considering and piloting a range of MOHO assessment tools.

During the group reflective sessions, the occupational therapists offloaded negative experiences, sharing their frustrations and difficulties regarding wider service issues and the barriers to implementing MOHO. They expressed their feelings of confusion (often surfacing as anger and resentment). There were differences of opinion and opposing cliques, and group members struggled for control. Moreover, disjuncture, or the 'troublesomeness' of new learning, occurred when therapists were challenged to reflect on their identities, considering who they were and what they knew. Even those therapists who appeared positive about the venture at the outset experienced personal and environmental barriers, which impacted their intentions to act. Another source of disjuncture was the discontinuity between what therapists were learning about MOHO within monthly group supervision and their practice within their teams. The process also had the effect of disrupting therapists' working lives.

The facilitator encouraged individual expression, helped people listen to each other, and gave and received negative and positive feedback. Negotiation was required to appreciate one another's perspective. Meeting with members individually, outside of group sessions, and identifying with them was important. In time, the monthly group sessions came to be viewed as an important means of support. A commonality of purpose emerged and colleagues came to appreciate that a more open and trusted sharing of one another's practice offered an improved means of developing confidence in their own professional judgements. Peer relationships, nurtured within the group collective, enabled opportunity for participants to examine their professional identity with one another. It was also evident that observing others engage led to personal decisions to act. A final indicator of the value of peer relationships was that a number of therapists began meeting on their own outside monthly sessions.

The reflection-action cycles provided participants the opportunity to become increasingly aware of practise constraints. MOHO came to be viewed as a means of problem solving practice dilemmas, mobilising and informing their decision making. Therapists needed to exercise autonomy to modify and adapt MOHO knowledge to meet their human and practice needs. Being active learners who could explore and examine their practice critically was key to this process. As therapists began to practise differently, they saw the impact of their change upon others (e.g., service users and multidisciplinary colleagues). Implementing MOHO theory into practice became an ongoing, self-reinforcing process

Although, the inquiry was successful, it was fraught with challenge. This did not mean that participants were not keen to advance their practice; but, it was not a comfortable or tidy process and it would take time for participants to see the results of their efforts. It was helpful to have a

facilitator who was not affected by in-house politics, not immersed in day-to-day practice issues, and who was a resource. The facilitator needed to be sufficiently pragmatic whilst reflexive throughout in being able to adjust and adapt, to neither take the lead nor sit back. She realised that regardless of her enthusiasm to invite all the therapists to view themselves as co-researchers of the study, this aspect of the process would need time and commitment to come to fruition. An important responsibility of the facilitator was achieving the right balance between incorporating and imposing knowledge. It was also important that the facilitator did not push participants to 'learn too fast'. Often, therapists would become discouraged or reluctant for very real reasons. Joint dissemination of the research findings has occurred via national conference and publication; further joint publications are planned.

Type 3 : Replication

BELLMAN, L., BYWOOD, C., & DALE, S. (2003) 94

BONIFACE, G., FEDDEN, T., HURST, H., MASON, M., PHELPS, C., REAGON, C., & WAYGOOD, S. (2008) 95

BOTHE, J., & DONOGHUE, J. (2009) 97

CHENOWETH, L., & KILSTOFF, K. (1998) 98

DENGLER, K. A., WILSON, V., REDSHAW, S., & SCARFE, G. (2012) 99

GREGOROWSKI, A., BRENNAN, E., CHAPMAN, S., GIBSON, F., KHAIR, K., MAY, L., & LINDSAY-WATERS, A. (2012) 100

HARRISON, A., & ZOHHADI, S. (2005) 101

KO, A., LEONTINI, R., NGIAN, V., HUGHES, I., CLEMSON, L., & CHAN, D. (2013) 102

PROCTOR, K., PERLESZ, A., MOLONEY, B., MCILWAINE, F., & O'NEILL, I. (2008) 103

PUOANE, T., SANDERS, D., ASHWORTH, A., CHOPRA, M., STRASSER, S., & MCCOY, D. (2004) 104

SORENSEN, E. W., & HAUGBOLLE, L. S. (2008) 106

***Bellman, L., Bywood, C., & Dale, S. (2003). Advancing working and learning through critical action research: creativity and constraints.* Nurse Education in Practice, 3*(4)*, *186-194. doi:*10.1016/S1471-5953(02)00114-2**

Five registered nurses participated in a study to explore the process of enabling nurse-led accredited change and development in clinical practice in the hospital ward recovery area where they worked. Their collaborative learning and working resulted in the initiation of three patient-focused projects: pre-operative fasting times in the older trauma patient; post-operative patient-controlled analgesia; inadvertent peri-operative hypothermia. Open communication enabled and empowered the front-line co-researchers to directly influence the development of the study. They felt able to share their ideas, the gaps in their knowledge, and recognised the importance of time for thinking and reflecting on nursing research and practice. Each of the co-researchers demonstrated ongoing positive and painful enlightenment through their own personal development and participation. Also the change process and potential impact on the quality of care for patients is not only to be seen within the recovery area but has been recognised both within the operating theatre and the surgical areas that are collaborating with the projects. The senior manager of the acute unit revealed: "I think it's [the whole study] got huge potential...The culture is changing. There are more people looking to become involved."

Boniface, G., Fedden, T., Hurst, H., Mason, M., Phelps, C., Reagon, C., & Waygood, S. (2008). Using theory to underpin an integrated occupational therapy service through the Canadian model of occupational performance. **British Journal of Occupational Therapy, 71*(12), 531-539.***

The aim of this study was to embed the theoretical tenets of the Canadian Model of Occupational Performance and its structures in a way that was appropriate to, and would be used by, all staff within an integrated health and social care setting. Initially enthusiasts were called upon to work in the localities and join a short-term steering group. This small group of self-selecting members of the service and the university lecturer, soon grew to include representatives from all geographical areas and services within the trust (n = 16-20). It was non-hierarchical and disseminated the notes of its meetings to the whole service through individual 'champions' of the model's implementation. Despite the attempt to include all staff in the action phases of the research (led by their own representative on the action research steering group), the steering group was a large group and, not all the members were present at all the reflection cycles, but they were crucial to the research's action phases

The steering group recognised their supervisory and influential role, but did not want staff to feel the model was being imposed upon them. The group discussed what resources were required to assist staff confidence and keep the momentum going. Given staff criticisms of communication and dissemination of up to date information the steering group recognised the need to engage both occupational therapists and the managers in the different organisations, and that management and senior occupational therapists needed to support and help maintain enthusiasm for the model within their teams. Thus issues, concerns and good practice were brought to the steering group meetings, and over time tools and materials were developed to help staff in the sharing of good work and solutions to issues and concerns.

The collaborative way of carrying out the research ensured the workability of the action. For instance, the steering group member who had previously experienced the dilution of the model's theory by its paperwork being implemented too early influenced the steering group to delay paperwork implementation. At the same time, other staff members were clamouring for its creation. The result following was that staff in their own settings began to create their own paperwork, which was then brought back to the steering group for further reflection and consequent action. The assessment and planning paperwork that has evolved through this process is now almost countywide, applicable to most areas, firmly embedded in the theory of the model. Another example concerns training. Champions in the acute hospitals produced a training package on the model's theory and use and the steering group became aware that other areas were keen to use this or a similar package and recognised that the training package was a good way of a team working together to strengthen understanding of the model, share how it could be practically adopted in individual clinical areas and address any issues or concerns. The steering group realised this sharing of knowledge would not have happened if the information had not been taken to a group steering the implementation of the model.

The steering group was critical in guiding the model's implementation, in sustaining motivation and energy across the service, and for communicating information across a wide staff group on an ongoing basis. Many heated discussions occurred and all members of the group found that their thinking about occupational therapy practice developed and changed. The group has continued to have an important

role in making decisions and recognising when staff needed re-energising. The process so far has taken 4 years and is ongoing.

***Bothe, J., & Donoghue, J. (2009). Using action research to develop a model of patient-centred day care*. Practice Development in Health Care, 8*(3)*, *152-160*. doi: 10.1002/pdh.290**

At a public hospital in New South Wales, Australia, day surgery nurses had become disenfranchised with nursing and disengaged in the patient relationship. This study sought to engage the nurses as a research team and make changes, take risks and be creative in revising the management of patients throughout their day surgery journey, and specifically, to develop a model of care that was safe, effective and patient centred for day surgery patients undergoing LC and UHR. The permanent staff of the day surgery unit, including the nurse unit manager, four full-time and five part-time registered nurses, participated in the research. At various stages of the research, senior nurse mangers, surgeons, anaesthetists, operating theatre staff and pharmacists were also involved.

The research leader worked with the research team over one year. The process of negotiation with the day surgery unit nurses to participate as a research team and initiate change was complex. Negotiation was also required to ensure that surgeons, anaesthetists, senior nurses, other wards and departments were aware and agreed to the changes proposed by the research team. The changes to practice were achieved through fortnightly meetings that enabled the team to talk to each other to identify practice areas that required improvement. The meetings gave the group the opportunity openly to discuss implemented changes in a supportive and structured environment. While the process needed to be able to let nurses develop over time, it also needed flexibility to be able to change clinical focus if urgent issues arose or issues were obviously causing staff anxiety. Collective decision making and consensus about the organization of the work became essential to the research team in their quest for improved patient management. Additionally, it was important to the research team to relay information to the next stage of care through accurate documentation.

As a research team, they learnt to discuss their understandings and the principles of quality nursing care, to think critically about their work, and to develop problem-solving skills in relation to the structure of day surgery patients' journey. The team members increased their understanding of the importance of timely, accurate and consistent patient education. In addition, understandings were enhanced between the research team and other departments, wards and Medical Officers through improved communication. The team aligned their values into a set of criteria – namely, patient safety, effectiveness of care and patient centredness – that were used within the group to evaluate all decisions related to changes in patient care. Proposed new changes were evaluated using the agreed principles of care for each stage of the patient's journey. As the research team became more confident to identify problems and be fully involved in decision making regarding the management of 'their' patients, they became responsible for those decisions and for the evaluation of the interventions they implemented.

As a result of the opportunity to communicate openly with others, in addition to the team's ability to think and discuss their work critically, their practice became more effective, safer for patients and patient centred. These changes were apparent to others, and provided a model of enablement that is now used elsewhere in the organization.

Chenoweth, L., & Kilstoff, K. (1998). Facilitating positive changes in community dementia management through participatory action research.* International journal of nursing practice, *4(3), 175-188. DOI: 10.1046/j.1440-172X.1998.00063.x

In a multi-cultural dementia daycare centre, staff, family carers, and academics undertook a study to create new ways of managing clients with dementia through a simple therapy program and to evaluate the process of implementing this program. The study was designed to respond to the emerging needs of the organizational partners and those of the center patients and their carers. Trust among all partners built slowly through ongoing dialogue and friendly interactions. Partners met regularly to support one another in the task and to reflect on common difficulties in providing day-care programs. Meetings were held to feed back the academic researchers' perceptions of the growth that had taken place throughout the research period. A written report of the results of the therapy program were presented to the center and made available to those who requested a copy.

Family carer support groups decided to continue meeting at the day care centre and to seek ways of contributing to the day care program in the future. Other centers in the area have asked the academic researchers to facilitate PRO studies with them, paving the way for ongoing change and empowerment for more groups of people involved in dementia care.

Dengler, K. A., Wilson, V., Redshaw, S., & Scarfe, G. (2012). Appreciation of a Child's Journey: Implementation of a Cardiac Action Research Project. Nurs Res Pract, 2012, 145030. doi:10.1155/2012/145030

At a Children's Hospital in Australia, the cardiac ward identified that a bead program could improve the experiences of cardiac patients and their families. Following presentation of the concept to staff by the cardiac ward nursing unit manager and cardiology clinical nurse consultant the program was discussed and investigated via literature search and communication with the developers of an existing bead program. A project team was established and met on a monthly basis to discuss project progress and issues arising, and to evaluate the process and its evolution with staff.

Evaluation was continuous throughout the program development phase, feedback from all relevant stakeholders taken into consideration, and changes were integrated as the project progressed. To ensure that stakeholders including clinical staff and families were involved in the project from the beginning, and that the program was responsive to the needs of staff and families, consultation took place with nursing and research staff, families, and representatives of the hospital units where children with cardiac conditions reside. Ongoing consultation between the project team and other units that were involved in the care of cardiac children and their families was also an important aspect. A Heart Beads resource folder was created containing the printed materials from education sessions, distribution forms, consents, and a journal for frontline ward staff to record candid comments and feedback from children, families, and other staff members relating to the program. Regular emails were sent to staff to ensure that they were informed about new developments and program feedback. Staff were also encouraged to use this mechanism to provide feedback and reflections to the project team.

The continual reflection and evaluation involved in the three-year project allowed relevant changes to be made and materials and processes refined, resulting in a more effective and sustainable program and enabled the project team and staff on the ward to maintain momentum. Evidence emerged through staff team building exercises that the Heart Beads program is seen as an emblem of the common goals of the staff from the different units. The program has united the cardiac teams from preadmission clinic staff to the cardiac ward staff, allowed for clarification and strengthening of the values of the cardiac team, and has enabled staff to engage more positively with the children and their families, which in turn facilitates enhanced therapeutic relationships. Details of the Heart Beads program, particularly relevant outcomes, have been communicated throughout the hospital and at local, national, and International conferences. The positive experience of Heart Beads families has attracted the interest of a cardiac support group, and the program is now being implemented nationwide with the vision that all Australian children hospitalised with cardiac conditions can benefit from Heart Beads.

The Heart Beads program began as a small project in the cardiac ward and continues to grow in popularity and significance. Throughout the development, implementation, and evaluation process, the Heart Beads program encountered numerous setbacks and challenges, many of which were overcome using the action research approach, as well as witnessing inspiring results and enthusiasm.

Gregorowski, A., Brennan, E., Chapman, S., Gibson, F., Khair, K., May, L., & Lindsay-Waters, A. (2012). An action research study to explore the nature of the nurse consultant role in the care of children and young people.* J Clin Nurs. *doi:10.1111/j.1365-2702.2012.04140.x

The aims of this study were to explore and analyse the nature of the nurse consultant role when working with CYP within a large tertiary referral paediatric hospital in a city in the UK, and to provide nurse consultants with support within the organisation as they embraced these new roles between January 2005 and January 2007. The study was facilitated by a nursing research fellow employed within the hospital and designed by all of the nurse consultants working with the research fellow and steering group. The primary method of data collection was through 12 monthly action research meetings, in which nurse consultants worked collaboratively as co-researchers with the research fellow to explore their emerging roles. Meetings were negotiated at the outset so that the length, frequency and timing were agreed by all and arranged around clinical work commitments. The agenda was collectively set by the nurse consultants at the start of each meeting. The facilitator ensured that turn taking took place. Each meeting lasted for two hours. Not all nurse consultants attended all meetings. The nurse consultants and research fellow attended one day of thematic analysis training. The nurse consultants met with the research fellow on a regular basis to analyse the data together. In this way, a number of themes were collaboratively identified and divided into subthemes through paired and group work. Once themes were identified, the nurse consultants agreed to work in pairs on the individual themes. Each took a theme to work on as the primary researcher and another to work on as the secondary researcher. The group then came together to organise the themes and subthemes into a framework.

While the research role seemed the hardest to put in place at the outset, by the time this study was completed, three of the five nurse consultants were undertaking PhDs, with two involved in predoctoral research in preparation for PhD registration. The research role for the nurse consultants required new skills and sufficient attention and space to flourish. Nurse consultants in this study initially underwent a dip in their clinical confidence as they sought to manage all role aspects. As their unique contribution to clinical care became clearer, their confidence increased and they developed the drive to undertake research.

The action research supported and sustained the nurse consultants in their roles, giving them the confidence to challenge boundaries in their work with CYP and families, and with other professionals in their specialties. The actions that came out of action research meetings included nurse consultants requesting to be part of committees and work groups to influence key decisions about CYP and families. This involvement made the roles highly visible while CYP's needs were kept firmly on corporate and strategic agenda. The outcome was a positive difference to the lives of the CYP and families treated in each service.

The nurse consultants in this study continue to meet as a group on a bi-monthly basis, using e-mail contact between meetings to support and advise one another. The next step for these nurse consultants is to further develop the research aspects of their roles. Their influence continues to extend beyond the organisation to influence national and international healthcare agendas.

***Harrison, A., & Zohhadi, S. (2005). Professional influences on the provision of mental health care for older people within a general hospital ward.* Journal of Psychiatric and Mental Health Nursing, 12*(4), 472-480. doi:*10.1111/j.1365-2850.2005.00868.x**

The aim of this study was to improve the care of older people with mental health problems in the general hospital. To ensure that the focus of the action and development cycles was directly related to the needs of the participants, it was necessary to undertake the exploratory work presented here. A professional working relationship with the nursing manager of the Older People's Unit was developed through working with her on previous projects. She was the essential link between the research team and the research participants.

It was through collaborative reflecting sessions that the implementation of practice changes progressed. Through discussion and critical reflection participants communicated their ideas and also their fears and insecurities regarding their job roles and responsibilities, and care capabilities. Ideas for changes to make emerged and were implemented. The aims of the project and the dependence upon collaboration between the participants required structured interaction between all of us.

Issues of engagement, ownership, and sustainability all impacted upon the research process and influenced the final outcomes. The changes to practice were not all fully implemented during the formal life of the project but have nevertheless provided a foundation for further practice development work with the staff. For many of us, our input has continued beyond these stages. The ward staff remains engaged with taking forward their amendments to practice, and we, as practitioners and "researchers", continue to support these changes by the ongoing facilitation of practice development meetings. Thus, collaborative reflection sessions have become an ongoing feature of work with the older people's unit and, through this process, we are able to continue to address many of the issues identified. Notably, the nurse participants described a change of thinking and a willingness to consider how they might address some of the problems associated with caring for older people with mental health needs. This study highlights the labor-intensive and complex nature of action research.

***Ko, A., Leontini, R., Ngian, V., Hughes, I., Clemson, L., & Chan, D. (2013). Quality and safety of prescribing practices in aged care and rehabilitation units in an Australian hospital*. Asian Journal of Gerontology & Geriatrics, 8*(2), 68-77*.**

This two year study aimed to assess an approach to change in medication management. The role of the researcher was to actively draw the clinical staff into the project, and to participate with them in the process of ongoing observation, reflection, learning, and action. This role was integral to engendering trust and understanding among the different professionals, and for teaching them and learning from them effective ways of using the findings to develop improvements in practice. The nursing unit manager was involved in recruiting co-researchers for the MAR [medication action research] group (health professionals from the hospital and from 4 aged care and rehabilitation units); coordinating various aspects of the project and actions taken; and conducting fieldwork.

Each member of the MAR group was allowed to comment, reflect on, and discuss their own views and practices, as well as those of other colleagues. They met on a monthly basis to revise and to build on emerging principles for safe medication management. Emerging data and findings were made accessible to participants and were adopted for making decisions about subsequent interventions, thereby improving teamwork. The MAR group presented a progress report to front-line clinicians and other staff in the aged care units to remind staff about the study and how it was progressing. Staff and the MAR group agreed to trial the use of a communication book in each unit to standardise the transfer of information among staff. This book became part of the hospitals' new procedures. Key participants from the MAR group emerged as the 'local champions'. They were willing to invest time and energy into the study and positioned themselves as able to assist with the implementation of interventions. As the study and action plan evolved, the network of participating health professionals expanded, as other staff in the hospital took part in the AR project. This extended network provided multiple opportunities to both researchers and participants for sharing information and implementing change. Following the implementation of the AR project, staff noted: "we were more willing to talk with each other about problems and finding solutions together."

Participation in the inquiry by health professionals was important as it fostered cooperation among clinicians and shared decision-making at different levels of interaction. Doctors, pharmacists, and nurses had an opportunity to work closely together on the common goal of implementing change to medication management. In the process, team members also gained a deeper understanding of effective and safe prescribing practices. By working closely with staff within the system, the researcher was also experienced in, exposed to, and made aware of the needs, expectations, and limitations experienced by staff within their workplace and roles. The feedback and participation by clinicians in the research helped the investigator to reflect, modify, and adapt the project accordingly. It is through the commitment and enthusiasm of the 'local champions' that an AR study on safe medication practice remained sustainable in the hospital setting.

Proctor, K., Perlesz, A., Moloney, B., McIlwaine, F., & O'Neill, I. (2008). Exploring theatre of the oppressed in family therapy clinical work and supervision. Counselling & Psychotherapy Research, 8(1), 43-52. doi:10.1080/14733140801889139

The aim of the study was to explore the application of Theatre of the Oppressed (TOTO) in clinical work with families and in clinical supervision. Research question: 'How would the involvement of family therapists in TOTO improvisation and performance contribute to ways of dealing with oppression in their clinical and supervisory practice?' Following 6 sessions of TOTO training with the BT [Breaking Through -- project name] facilitator, twelve volunteer family therapists formed a cooperative inquiry research group and met for another eight two-hour sessions, over a period of around 10 months sessions, with the BT facilitator. Group participants each kept a journal that served to mark their experience and learning, bringing this journal to each session and sharing their experiences. At each session the group members engaged in action techniques to explore and reflect on experiences of oppression that might lead to useful insights for their work. Group discussions were recorded via audiotape in each session, then transcribed and analysed between sessions. Descriptions of group activities were also noted down and analysed by a BT project worker/ family therapist.

In the group sessions, the TOTO group explored non- verbal representations or images of 'what oppression means to us'. In small groups, the images of each individual group member were woven together to create a group-devised piece. Each small group then performed these short pieces to the other groups. When one of the images evoked strong responses in the audience group members, it was replayed, giving members of the audience the opportunity to stop the action, replace characters in the image, and have a go as spect-actors at doing something differently These sessions generated further discussions around power, invisibility, gender, having a voice, and 'normality' that went beyond individual/ family dynamics and spoke to the importance of family therapists understanding their own dominant cultures and the assumptions that inform their therapeutic practices. TOTO workshops have also been incorporated into the Bouverie Centre's annual 'closed days', a two-day in-service workshop for the staff group as a whole to explore difficult issues that have arisen in the agency throughout the year.

We found that the cooperative inquiry research process was really useful in terms of examining our practice more rigorously and sharing ideas around diverse applications of TOTO. TOTO offers therapists and trainers a process whereby they can model their own agency, provide an opportunity for their clients and students to 'have a go', encourage those with whom they are working to imagine and enact different solutions, express emotions within a containing but creative process, and most importantly, despite the gravity of the challenges sometimes faced, have some shared fun along the way. Growth and development in counselling and supervision is maximised within challenging but positive and supportive environments. The cooperative inquiry action research process provided just such a context.

Puoane, T., Sanders, D., Ashworth, A., Chopra, M., Strasser, S., & McCoy, D. (2004). Improving the hospital management of malnourished children by participatory research. International Journal for Quality in Health Care, 16*(1), 31-40*. *doi:*10.1093/intqhc/mzh002

This study sought to improve the clinical management of severely malnourished children in rural hospitals in South Africa. The task of the hospital nutrition team (1 doctor and 2 nurses from each hospital) was to investigate the reasons for the high mortality among severely malnourished children during treatment and to suggest actions. After completion of the data analysis, the hospital nutrition team and facilitators met for 2 days in August 1998 to review the research findings and formulate a plan for improving the management of severe malnutrition. Findings stimulated a very productive discussion among the participants concerning lack of knowledge, motivation, and availability of resources.

The team expressed appreciation of the research process, and nursing staff stated that their involvement in the process had already led to modification of some of their practices (e.g., improvement in the frequency of weighing of children, the pharmacist had been contacted to ensure a reliable supply of medicines, and more space to accommodate malnourished children was provided). They felt ready to improve the quality of care further. They drew upon the WHO guidelines to develop a specific action plan for each hospital and identified practices needing improvement and constraints. To sustain the enthusiasm generated during the 2 days, and to provide support to the staff, in October 1998 a second 2-day workshop was held to review progress in the implementation and to provide further training. The managers for provincial and district maternal and child health and nutrition also attended. Staff from both hospitals shared their achievements. They reported several changes in practices and also improved resources. A treatment protocol was agreed upon and a time line was formed for the issues that needed action at the hospitals, and at the regional and provincial levels. A checklist of performance indicators to monitor the implementation was developed and a nurse from each hospital developed a monitoring schedule. Regular oral feedback was given to the ward staff. The monitoring process identified areas for further training, which facilitators provided.

In the process of exploring changes to be introduced, availability of resources, and difficulties to be dealt with in implementing improved care. Involving the team in all stages of the research encouraged them to reflect on their own practices, to plan and implement changes for better quality of care, and to develop a protocol to suit their needs and available resources. This experience strengthened their self-confidence to change the situation themselves, despite working in difficult circumstances, and to motivate and train other staff, leading to a commitment to improved performance. The process also built the research skills of the team, created ownership of the project and helped develop awareness among others who were indirectly involved in the care of malnourished children of the need to introduce changes.

The external facilitators encouraged critical thinking and offered alternative ways of viewing the situation, provided technical expertise and training, and offered support and encouragement to the paediatric staff, listening to issues raised and helping them to explore the implications of introducing changes. Another important factor was the involvement of relevant hospital, provincial, and district management personnel who were able to make policy changes and undertake various negotiations with

the district health manager, for instance. Their involvement was also important for sustaining quality of care. Following the experience in these two hospitals, the process has been scaled-up within the province and staff at a further 23 hospitals have been trained.

Sorensen, E. W., & Haugbolle, L. S. (2008). Using an action research process in pharmacy practice research-A cooperative project between university and internship pharmacies. Research in Social and Administrative Pharmacy, 4(4), 384-401. doi:10.1016/j.sapharm.2007.10.005

The overall aim of the three-year Pharmacy-University (PU) study was to contribute to the quality development of pharmacy practice and pharmacy practice research in the area of pharmaceutical care services. The existing structure used as the basis for the study was the University's ''pharmacy internship program,''. A steering group and various ad hoc working groups (interview group, questionnaire group, process group, hospital group, and dissemination group) were set up. Members of the working groups were also members of the steering group. Once the working groups were organized, the steering group's meetings could be limited to twice a year.

The pharmacy students were the local leaders of the study in their respective internship pharmacies. They introduced the study, collected data, and presented the results to staff. Accordingly, pharmacy staff was invited to take action in the pharmacy on the basis of new knowledge presented to them by the pharmacy students. All activities were subject to evaluation and subsequent reflection in the steering group and among participants at the annual preceptor days. Reflection gave rise to adjustments, changes, and concluding some parts of the project.

The basic tenet of the project was the learning aspect for all parties involved. The steering group considered the preceptors' practical knowledge and researchers' theoretical knowledge as equal prerequisites for being able to carry out the project. The learning was linked to how closely the participants were involved in the project. Because of their commitment to the study, the researchers and practitioners in the steering group were more actively participatory than the internship pharmacies and pharmacy interns.

The study helped to bring about genuine collaboration between the institutions in pharmacy practice research and development. Moreover, beyond the publication of traditional scientific articles, the study gave rise to numerous reports, oral presentations, a homepage, poster presentations, and so on for the benefit of all Danish pharmacies. Today all Danish pharmacies can request, free of charge, 5 different counseling models based on tools and results from the PU study, entitled ''The Pharmacy Package,'' from the study's homepage. Also, the PU study influenced the choice of the pharmacies' activities, and since 2004 all Danish pharmacy students have practiced clinical pharmacy during their 6-month internship period. Another change in knowledge came from the insight the study provided into effective learning methods for students during pharmacy internships, and new knowledge and experience acquired by the leaders from steering an action oriented study. Another consequence was that the participating internship pharmacies felt like part of the project and claimed ownership, but they did not feel obligated by project results.

The steering group gained experience in using the following principles: The AR cycle; AR project management; Focus on stakeholders in the study; Focus on study start-up Action, reflection, and learning processes; Wide use of existing structures. Members of the steering group were positive about the AR working form and would like to use it in future studies, though it was pointed out that a better structure of the steering group's work is needed; in other words, the AR form was considered

unstructured compared with other forms of management. The diversity in knowledge and skills in the steering group was a very important ingredient for the success of the study.

Type 4: Initiation

GALVIN, K., ANDREWES, C., JACKSON, D., CHEESMAN, S., FUDGE, T., FERRIS, R., & GRAHAM, I. (1999)....109

HEYNS, T. (2008)....111

LOFMAN, P., PIETILA, A. M., & HAGGMAN-LAITILA, A. (2007)....113

TAYLOR, B. (2001)....114

Galvin, K., Andrewes, C., Jackson, D., Cheesman, S., Fudge, T., Ferris, R., & Graham, I. (1999). Investigating and implementing change within the primary health care nursing team. Journal of Advanced Nursing, 30(1), 238-247. doi:10.1046/j.1365-2648.1999.01069.x

This study sought to, among other things, define new roles and responsibilities for members of a nursing team. A number of workshops with the nursing team examined the findings from the data, explored key areas of patient needs and ideas about how practice could be changed, and made suggestions about new roles and effective team working. Regular meetings facilitated by the researcher were implemented to develop ways for the team to support each other and feed-back to each other and the researchers regarding successes and problems. The project team kept reflective diaries and the nursing team were invited to write down their thoughts and feelings about the research. Issues which arose in the reflective diaries were discussed within the regular project team meetings and all team members were encouraged to share their views. This process facilitated collaborative working and provided an opportunity to clarify any confusion, air any tensions, and to agree a way forward. The team prioritized three areas for change based on themes from the data and their own discussions about practice development that were feasible within the study's resources. Drafts of protocols, assessment tools and procedures were agreed upon. Issues with implementation of the assessment were brought forth and dealt with. The changes progressed at different rates throughout the project period, with some problems due to time constraints and busy schedules. Also, some changes came about slowly and required many meetings with the researcher and staff. Delays in visible progress within the practice added to tensions and resulted in some conflict.

Collaboration was achieved, but considerable effort was required to maintain it. Team members were helped to identify the changes they wished to make, but some difficulties were experienced, particularly around issues of communication, understanding roles and competing demands. Group boundaries and responsibilities in the research were not clear. There were also confusions about the role of the researcher practitioner (insider) who was also a lecturer in primary health care with teaching commitments; her role in the research team did not meet expectations. At one point there was a breakdown in communication between the collaborative parties and tensions arose. For example, in developing a new service for leg ulcer management it became apparent that the nursing team expected more clinical involvement from the research team. However, the philosophy of the research approach required change to be managed from within the practice. As a compromise, and to facilitate progress, an additional member of the research team worked in the practice with patients in preparation for the proposed changes. Also, the team revisited group boundaries and re-negotiated responsibilities and timescales. Locating ownership of change with the team was sometimes difficult. At first the research team had a very active role and the Primary Health Care Team (all members of staff, not just nurses) a passive one. This reversed as the study developed and was facilitated by regular informal meetings with the team. The project team made feedback as unthreatening as possible by emphasizing the shared nature of problems but with hindsight more, regular feedback sessions were needed.

Team members and researchers (4 academic staff and a research practitioner) have provided positive feedback on the changes. Outcomes of the project include a plan for a responsive service to patient

need and flexible team-working. The new model of working as proposed in this study will need to be piloted and an evaluation is planned.

Heyns, T. (2008). A journey towards emancipatory practice development. *(D.Litt. et Phil.), University of South Africa (South Africa).*

The aim was to collaboratively plan a journey towards emancipatory practice development to address the emergency situation and enhance the possibility of creating a future for the nurse practitioners in the Accident & Emergency unit. A Practice Development Group (PDG) was negotiated with the practice leaders (the unit manager and clinical facilitator). These three individuals held eleven weekly discussions to collaboratively determine and reach consensus on their roles, ethical responsibilities, values, shared vision, purpose of the action research for practitioners, and possible activities. The researcher provided summaries of fieldwork to the practice leaders prior to discussions. The PDG reflected on a way forward, used mind maps and set criteria to evaluate the worth of the project. They decided not to include middle and top management (outsiders) but insisted that the practice leaders and nurse practitioners (insiders) resolve the situation themselves.

The PDG used findings to plan actions that could potentially resolve the challenges and change the toxic environment to an enabling environment. The nurse practitioners were included in the planning and monitoring of the actions and reflecting on and re-planning the actions when applicable. The unit manager involved them in managerial tasks and teamwork, fostered motivation by gaining their trust, supported them by having an open-door policy and giving them the opportunity to consult her if they needed assistance, and valued their input. Nurse practitioners' innovations and hard work were acknowledged and celebrated throughout, and the unit manager noted that this motivated them and indicated that their successes should be celebrated more often.

Examples of enlightenment include the nurse practitioners who became aware of the gaps in their own professional knowledge and skills, and the consequences of this on patient care. Emancipation of the unit manager and nurse practitioners could also be observed. Several unforeseen achievements occurred, such as new partnerships, including one between the two practice leaders who indicated that they did not realise how important it was to collaborate and work together at first, but at the end they regarded their partnership as the most valuable aspect gained during the project. The clinical facilitator also developed a partnership with two clinical facilitators in the critical care units. They held monthly meetings and worked together with the CPR champions training. Five professional nurse practitioners enrolled for the A&E programme, and the clinical facilitator and two nurse practitioners enrolled for master's degrees. Several spin-off initiatives that focus on patient-centred care also occurred.

The project demonstrated that supportive partnerships evolve over time. The supportive partnership of the PDG may have been preceded by mistrust and ignorance of each other's values and needs but evolved over time as personal and organisational relationships solidified. To the researcher, this lesson was possibly the most important of all the lessons learnt. Trusting people and believing in their abilities creates opportunities for empowerment and emancipation. However, a positive attitude, coaching and support were essential ingredients for the success of the project. The three PDG members recognised the potential benefits of working together, whether these were short-lived or durable and continuous. The practice leaders indicated valuing working as a group who supported and encouraged each other, and that this together with their shared vision and action plan motivated and encouraged them to implement the actions.

Lofman, P., Pietila, A. M., & Haggman-Laitila, A. (2007). Self-evaluation and peer review - An example of action research in promoting self-determination of patients with rheumatoid arthritis.* Journal of Clinical Nursing, 16*(3 A), 84-94. doi:10.1111/j.1365-2702.2006.01574.x

This two and half-year PAR study sought to develop of a nursing model and promote self-determination for orthopaedic patients with rheumatoid arthritis.
Peer review was held as focus groups. The head nurse of the ward had previously chosen the participants.
The evaluation instruments were developed in cooperation with the nursing staff. The researcher conducted these meetings.
The nurses described their attitudes as changing positively towards self-determination because the participants had focused more attention towards self-determination of the patient and promotion of patient participation. The nurses were attentive to patient opinions and had fulfilled their wishes more often than previously. They found work went more smoothly due to the rearrangement of nursing practice.
On the whole, there was an improvement in the development of patient-centred nursing. Autonomy, based on respect for patient ability to make choices and decisions, was an area where the nursing staff had succeeded well. This was especially noted through encouragement of shy patients to participate in the decision-making process. On the other hand, the collaboration within the nursing staff and the development of nursing practice needed as much improvement as improving information between nursing staff and patients.
Self-evaluation and peer review as evaluation methods helped the nursing staff to make the concept of selfdetermination more concrete. In being complementary to each other, self-evaluation and peer review gave the chance for nurses to consider self-determination from various aspects.
The nurses felt improvements had happened in many fields connected to patient self-determination. However, there is no guarantee of the maintenance of this development after this PAR study.

Taylor, B. (2001). *Identifying and transforming dysfunctional nurse-nurse relationships through reflective practice and action research*. International journal of nursing practice, 7(6), 406-413.

To facilitate reflective practice processes in RNs to raise critical awareness of practice problems they face, work systematically through problem-solving processes to uncover constraints against effective nursing care and improve the quality of care given by nurses. Nurses kept journals, as a method of gathering data, and met weekly for 1h to discuss clinical problems raised in them. Researchers described the kind of group dynamics that they hoped would be fostered so that trust, openness and confidentiality could be established and maintained. These included applying the general rules of professional confidentiality as practised already in work settings to the group, a respect for others' feelings, informing the group if an area being shared was confidential as sensitivities may vary, listening carefully, acknowledging the person speaking if only with attention and a nod of the head, refraining from criticism and unnecessary advice, and withholding the desire 'to fix things' except in cases where that was the explicit intention. The researcher acted as a group facilitator and as a guide in the research processes by writing minutes, preparing agendas and contributing as appropriate. Data analysis included an analysis of journal experiences by individual and group critical reflection, and problem-solving strategies. Group discussion also identified the specific nature and determinants of the problems, the most appropriate methods to investigate problems further and the most practical and useful plan of action. Descriptions of participant observation were analysed individually using a reflective analysis method and collectively by group discussion. In each action research cycle, the findings were pooled and discussed and the appropriate action was planned and taken.

Issues raised grouped into two main areas: professional relationships and professional identity. The two categories were discussed, and it was realized that they were related in that if the professional identity issues were remedied, professional relationships would most probably benefit also. The group decided to focus on professional relationships and to see if some of the other issues from professional identity are also addressed along the way.

Two stories were shared relating to the use of the action plan. The first story was about a situation in which the researcher felt reactive, recognized her feelings and turned it around so that it was no longer an issue for her. After group discussion, the researcher said that she might speak with the nurse to clarify her position. She felt that she had begun to establish a culture of positive strokes by her action in being self-reflective and nonreactive. The second story involved a researcher's use of many of the strategies in the action plan that turned out to be of little use given further events over time, which exacerbated nurse–nurse relationships. Researchers were aware of the situation and provided other perspectives on the same situation. As the perspectives unfolded, it became apparent that the situation was very complex and that it involved nurses' equal willingness and ability to communicate openly and directly. Thus, the first try of the action plan resulted in stories with different outcomes.

During the last meeting, researchers agreed that there was a need for ongoing connections so that the spirit engineered in the meetings could continue. This was deemed possible if researchers gathered for regular conversations and support, and that they would be coached to maintain their own group processes for any problems they might have. While they agreed that the processes had been helpful,

they also acknowledged the need for the role of an outside facilitator. Unfortunately, the group did not continue in a self-supporting fashion the following year, although positive feedback is still being received about the processes.

All 4 types of OPR

FAGERMOEN, M. S., & HAMILTON, G. (2002) 117

KHRESHEH, R., & BARCLAY, L. (2007) 118

Fagermoen, M. S., & Hamilton, G. (2002). **[Urological patients' perceptions of nurses' information before and after surgery](). Nordic Journal of Nursing Research & Clinical Studies / Vård i Norden, 22*(4), 38-43*.**

The purpose of this part of the project was to improve patient information before and at admission for trans-urethral resection and to explore the effect of the changes in the information practices as perceived by the patients. The highly supportive head-nurse selected nine enthusiastic nurses judged to have the necessary professional background and interest to work on the project. Two work-groups were formed, each group a mix of experience and expertise. The nurses identified the problem to be solved and were active participants in the process of change as equal partners with the researcher who had the role of facilitator who used a non-threatening, supportive, and accepting mentoring style and gave credit, guided and advised throughout. The researcher was responsible for the agenda and the minutes from project meetings. All met frequently to collectively discuss the work of each group. They developed a welcome brochure the use of which for all patients admitted to the ward is now regular practice, and other brochures that are sent patients when they receive their date for admission, also now regular practice. Indeed, admission of patients by one nurse each day is now a well-established practice with benefits for all patients, not only the trans-urethral resection -patients. Additionally, guidelines were necessary to ensure that all patients got a certain amount of information at discharge. To evaluate the changes, given no adequate instrument was found, the researchers worked with the nurses to develop an instrument, reaching consensus on topic, readability (literacy level), relevance, and ease of use for the patient. Guidelines for administering the data-collection were established and nurse was designated to do this. The hospital financed a course in SPSS for this nurse, who then was able to participate in developing codebooks and to carry out data-entry.

The pace of the study was slower than anticipated given a lower than usual admittance rate of trans-urethral resection patients. This affected the implementation that had been planned to coincide with the merger between the project ward and another urology clinic. The issue of ownership was an important concern. By the end of 2000 most nurses on the ward had not been part of the processes the year before. They received information informally by the nurses closely involved in the project and formally by the nursing professor who met with smaller groups of staff to inform and include them in the ongoing processes.

Positive outcome have resulted for patients and staff through the project. The new brochures improved the patient information, and patients valued the nurses' interaction and approach, and appreciated the correspondence between the information in the brochures and what went on while in the hospital. Re-designing the brochures benefited staff as well. Structuring the admission talk created a clearer, concise and consistent approach for imparting information. Moreover, as the discussions about the discharge talk evolved, the nurses recognised other areas that needed attention. They identified a need for standardisation of the nurses' talk with patients on admission and subsequently developed guidelines for this event.

Khresheh, R., & Barclay, L. (2007). Practice-research engagement (PRE): Jordanian experience in three Ministry of Health hospitals. Action Research, 5(2), 123-138.

Leaders in the Ministry of Health, hospital managers, and health professionals working in the maternity departments in three hospitals agreed that high quality clinical data for the care of mothers should be a priority and that the current poor records needed to be improved. The shared goals guided the researcher and practitioners in their joint work and created commitment for the considerable effort needed for the research to succeed. Research engagement groups were established at different levels of the health system and in the three hospitals, namely the 'National Steering Group', the 'Local Leadership Group' and 'Local Action Group'. The engagement between the support groups, practitioners and researcher was organized, managed and sustained by the field researcher, balancing the different values, goals, perspectives and capacities of the researcher and practitioners.

Discussions raised practitioners' awareness of the problem by presenting them with an analysis of their own baseline data. This helped to build motivation for change as they realized their own data was of poor quality. The content of the JCBR was renegotiated, tested and modified during meetings. Further discussions and negotiations resulted in agreement on the level and degree of commitment. The National Steering Group led the Implementation and Local Leadership Groups and Local Action Groups were involved in the implementation of the new JCBR. Jointly planned training sessions were conducted to enable practitioners to use it. The local director encouraged staff to become actively involved in the implementation process which helped increase staff's commitment and enabled continuous feedback.

Findings from the evaluation of the JCBR were fed back to the Local Action Groups and Local Leadership Groups and discussed in meetings, and their reflections together with the findings were reported to the National Steering Group in the Ministry of Health. The final results of the project were presented, by invitation, at a national public forum hosted by the National Steering Group. This inclusive process allowed the project to become the jointly owned work of the Ministry of Health and the researcher, and indicated the ultimate success of the practice-research engagement process.

The engagement between the researcher and the practitioners challenged current practices, identified effective improvements and developed a tool, the JCBR, which was based on both research and practice. The frequent interaction between the researcher and staff during the fieldwork and the co-operative relationship that shaped this interaction, created opportunities for the researchers and whole team to reflect, analyse and make change during the implementation process. This resulted in rapid problem solving and was used to keep staff informed and provide supportive feedback to them. Moreover, information collection and feedback to staff led to joint problem solving so that organizational development became not only a process of organizational improvement but also a process of mutual and liberating inquiry. Effective communication, clear and shared goals and establishment of joint involvement and shared ownership proved effective strategies that were adopted to enhance change and were successful in preventing resistance. Staff from all three hospitals have continued using the JCBR and are still collecting the statistical summary copies of the new record and sending them to the researcher for analysis. The first author has been invited to help in a new project for the Ministry of Health that aims to improve the quality of records in maternity care in all the hospitals in Jordan.

An unpredicted result was that the training sessions, focus groups and meetings helped medical and midwifery professionals, in the three hospitals come together for the first time interact positively, find common interests and begin to work in teams focused on this project's goal rather than as different status individuals. Moreover, relationships built between the research team and the action research groups have evolved into long-term collaborations on national and local levels, and have been sustained subsequent to the research being completed.
