# Supplementary material for: A systematic mixed studies review on Organizational Participatory Research: towards operational guidance
Source: BMC Health Serv Res. 2018 Dec 22;18:992. doi: 10.1186/s12913-018-3775-5 (PMC6421946; doi:10.1186/s12913-018-3775-5)
Supplement: Supplementary file 4 — Coding trees. This document includes screenshots of the coding trees with the number of OPR summaries (sources) per code and number of codes per summary (references). (PDF 199 kb) [file 12913_2018_3775_MOESM4_ESM.pdf]

Coding trees with number of OPR summaries (sources) per code and number of codes per summary (references)

Nodes

Name	Sources	Referenc
01. Work group composition (effect of)	19	27
01.1 Diverse membership (management, clinicians, service users, support staff) involvement is beneficial	17	23
01.2 Continuity of WG -- trust, commitment, productivity	4	4
02. Collective data analysis ...	12	18
Helps gel the WG	2	2
Leads to consensus and meaningful results	2	2
Provides opportunities to reflect & discuss & make evidence informed decisions for change	9	14
03. Learning of, Discussing resaerch findings leads to ...	16	31
...preparation, readiness for evidence informed change	14	21
- EB Discussing findigns validates perceptions or raises awareness	3	4
... actual evidence informed change	5	6

Nodes

Name	Sources	Referenc
04. facilitators of change	67	170
04.1 COLLABORATION is essential to achieving change	54	96
04.1.1 TEAM REFLECTION contributes to achieving objectives	9	11
04.1.2 Meetings provide a space for interaction, discussion & reflection which is crucial to achievemen	15	17
04.1.3 INTERACTION, DISCUSSION contributes to improved relationships, communication & collabora	34	51
04.2 Effective meeting faciliation	20	26
04.3 COMMUNICATION	19	26
08.6.1 Effective (open, 2-way, transparent, ongoing, right language) communication WITHIN the WG is	12	15
08.6.2 OUTSIDE the WG	8	11
04.4 Doing the work overTIME is beneficial	7	8
04.5 Good working relationships, partnership, leads to change	6	8
04.6 COMMITMENT helps achieve objectives	6	6
06. Org and individual's changes in KAB resulting from the OPR lead to Extra benefits	21	31
Individual KAB changes resulting from the OPR lead to Extra benefits	13	15
Changes in attitudes leads to Extra benefits	5	5
Changes in behaviours (actions, practices) lead to Extra benefits	4	5
Changes in knowledge leads to Extra benefits	5	5
Org changes resulting from the OPR lead to Extra benefits	12	16
